# *Mycobacterium tuberculosis* diversity and macrophage heterogeneity dictate phagosomal acidification

**DOI:** 10.1128/spectrum.03762-25

**Published:** 2026-07-20

**Authors:** Matteo Chiacchiaretta, Rita Sorrentino, Nadia Bresciani, Alessandra Aiello, Valentina Vanini, Samuel Zambrano, Davide Mazza, Federica Cugnata, Daniela Maria Cirillo, Alessandra Agresti, Delia Goletti, Paolo Miotto

**Affiliations:** 1 Emerging Bacterial Pathogens Unit, IRCCS San Raffaele Scientific Institute, Milano, Italy; 2 Translational Research Unit, National Institute for Infectious Diseases-IRCCS L. Spallanzani, Rome, Italy; 3 Unità Operativa Semplice (UOS) Professioni Sanitarie Tecniche, National Institute for Infectious Diseases-IRCCS L. Spallanzani, Rome, Italy; 4 Vita-Salute San Raffaele University 18985, Milan, Italy; 5 Division of Genetics and Cell Biology, IRCCS San Raffaele Scientific Institute, Milan, Italy; 6 Experimental Imaging Center, IRCCS Ospedale San Raffaele 9372, Milan, Italy; 7 University Center for Statistics in the Biomedical Sciences (CUSSB), Vita-Salute San Raffaele University 18985, Milan, Italy; University of Hawaii at Manoa, Honolulu, Hawaii, USA

**Keywords:** phagosome acidification, autophagy, apoptosis, single-cell, live confocal microscopy

## Abstract

Therapies counteracting pathogen-induced immune modulation of the host responses represent a promising improvement for tuberculosis treatments. Despite the documented role of host immune heterogeneity and bacterial genotypic background in determining infection outcomes, their interplay remains largely uncharacterized. We investigated the *Mycobacterium tuberculosis* (MTB)-human macrophage interaction, considering both macrophage phenotypic variability and MTB genetic diversity. Using single-cell techniques, we show how diverse MTB lineages fine-tune phagosome acidification differently based on macrophage phenotype (M1, M2), revealing very heterogeneous host-pathogen interactions. Our findings underscore the multiplicity of outcomes due to the combinatorial interplay between MTB lineages and macrophage phenotypes, which may have implications for the proper design of host-directed therapies.

**IMPORTANCE:**

The intricate interplay among host, pathogen, and environmental factors significantly contributes to susceptibility and clinical presentation of tuberculosis. These multifaceted mechanisms are often overlooked in tuberculosis studies, hindering our comprehensive understanding of infection progression and impeding the development of effective host-directed therapies, which offer benefits such as reduced drug resistance emergence and heightened host compatibility. Contrary to the prevailing hypothesis, the results observed in this study demonstrate the nuanced response of different *M. tuberculosis* lineages within distinct macrophage phenotypes in terms of phagosome acidification. The findings of our investigation delineate a crucial yet under-characterized aspect of tuberculosis pathogenesis, emphasizing how the interactions between different *M. tuberculosis* lineages and macrophage phenotypes could significantly influence the efficacy of host-directed therapies, particularly those targeting phagolysosomal maturation. More broadly, recognizing the impact of both *M. tuberculosis* and macrophage heterogeneity is paramount in the development of effective strategies to combat tuberculosis.

## INTRODUCTION

While significant progress has been made in understanding the pathogenesis of tuberculosis (TB), we still need to reach the disease elimination goal. *Mycobacterium tuberculosis* (MTB) resides within macrophages, which are crucial for granuloma formation and serve as a survival niche for the bacteria (1). Within macrophages, MTB encounters various stresses and evolved strategies to cope with them (1). Among others, MTB has the capacity to inhibit phagosome maturation and block acidification of the compartment, modulate apoptosis, and inhibit reactive oxygen species production (2).

MTB has been considered a highly clonal organism, and the laboratory strain H37Rv is largely used as a reference for research studies. However, the MTBC consists of lineages that differ in transmission capacity, proliferation in the host tissues, ability to evade host immunity, and propensity to acquire drug resistance (3, 4). More broadly, the distribution of lineages also reflects the reciprocal host-pathogen selective pressure leading to co-evolution and adaptation (5). The MTB complex (MTBC) comprises multiple phylogenetic lineages, with current classifications recognizing up to 10 human-adapted lineages (6, 7). These lineages are often grouped into two main phylogenetic clades: ancient (e.g. L1, L5, L6, L7) and modern (e.g., L2, L3, L4) clades. Ancient lineages are geographically restricted and less virulent compared to modern ones and are referred to as specialist strains (SPEC) (8). Modern lineages are most virulent and geographically widespread, and for this reason, are referred to as generalist strains (GEN) (8). The modern lineages 2 (Beijing), 3 (CAS/Delhi), and 4 (Haarlem, H37Rv) bear a large genomic deletion known as the MTB-specific deletion 1 region (TbD1) and are associated with major TB epidemics and higher transmission rates compared to Lineage 1 (L1) (Indo-oceanic) and L5 and L6 (*M. africanum*) (9). Modern MTB strains, in general, induce an early inflammatory response, which is weaker than those produced by ancient ones (4, 9).

Despite this microbe-centric classification, pathogenicity and virulence are nowadays recognized as functions of the host’s susceptibility and resistance (10). In this context, host heterogeneity at various levels should be taken into consideration. Besides genetic variability among individuals, human alveolar macrophages display substantial functional plasticity and have been reported to show mixed activation states, encompassing features classically associated with both M1- and M2-like macrophages (11, 12). However, despite extensive work on macrophage activation and polarization in TB (13), direct experimental comparisons of defined macrophage polarization states across diverse MTB lineages in controlled infection models remain limited.

In this study, we explore the MTB-human macrophage interactions at early time points post-infection (p.i.), considering both macrophage phenotypic variability and MTB genetic diversity. Single-cell approaches allowed a deep characterization of the heterogeneous host-pathogen interactions. Even small numbers of MTB bacilli can initiate infection *in vivo* (14), and at low multiplicities of infection *in vitro*, only a subset of phagocytes becomes infected, while others remain unexposed. These heterogeneous dynamics create distinct populations of infected and bystander cells, making single-cell analyses uniquely suited to interrogate processes, such as phagosomal acidification with high precision (15, 16). We evaluated the inhibition of acidification in pathogen-containing compartments, autophagy, apoptosis, and necrosis in classically (M1-like) or alternatively (M2-like) activated THP-1-derived macrophage-like cells infected by different MTB lineages, as well as the survival rates of both macrophages and bacteria.

Our study indicates that infection outcomes are shaped by the interplay between MTB lineage diversity and macrophage activation states, with phagosomal acidification emerging as one important component of this interaction. In addition, our data point to broader remodeling of lysosomal pathways, potentially reflecting differences in macrophage activation status and endolysosomal system dynamics.

These findings highlight that MTB lineage diversity contributes to differential engagement of host pathways. In this context, variation in phagosomal acidification responses may be particularly relevant for host-directed therapies, as interventions targeting endolysosomal pathways and phagolysosome maturation—such as metformin, imatinib, or pravastatin— could exhibit strain-dependent efficacy. Reliance solely on laboratory reference strains may, therefore, limit the translational relevance of such approaches. Collectively, this study provides a framework for incorporating both host and pathogen heterogeneity into host-pathogen interaction studies and for refining strategies to evaluate host-directed therapies.

## MATERIALS AND METHODS

### *M. tuberculosis* strains

Two clinical isolates representative of ancient (L1, L6) and modern lineages (L2, L4) and previously characterized were kindly provided by Prof. Stefan Niemann (Research Center Borstel, Borstel, Germany) (17). *M. tuberculosis* H37Rv NCTC 7416, *M. tuberculosis* H37Ra NCTC 7417, and *M. bovis* BCG NCTC 5692 were also included. Strains were classified and grouped according to the definitions provided in Stucki et al., related to the ecological niche width of MTB strains (Table 1) (8), resulting in ≥3 independent biological replicates per group, ensuring both single-cell resolution and robust biological replication. This grouping has been referred to as “geographical distribution and host adaptation” (geo-host adaptation) throughout the manuscript. Additional classifications are also provided for simplified comparison with other studies. All mycobacterial strains were engineered to express mCherry (Ex/Em 587-610 nm) from a low copy number plasmid. Briefly, bacteria were cultured in liquid 7H9 medium supplemented with Albumin Dextrose Catalase Oleic Acid (OADC) (Becton Dickinson, New Jersey, USA) and 0.05% Tween 80 (Sigma Aldrich, St. Louis, MO, USA) until OD_600_ reached 0.4–0.6. Bacteria were centrifuged and washed twice with 10% glycerol to eliminate salt and contaminants, achieve the electro-competence, and then resuspended in 10% glycerol for frozen storage. Competent cells were electroporated (2,500 V, 25 μF, 200 ohms) with 2.5 μg of salt-free plasmid and selected on 50 μg/mL hygromycin (Roche, Basel, Switzerland) 7H10 agar plates (Becton Dickinson, New Jersey, USA) (18). For macrophage infection, the strains were thawed on selective Middlebrook 7H10 agar medium supplemented with 10% OADC supplement and 50 μg/mL hygromycin. A single colony was picked and used for inoculating Middlebrook 7H9 liquid medium supplemented with 10% Middlebrook OADC supplement, 0.05% Tween 80, and 50 μg/mL hygromycin, and incubated at 37°C until OD_600_ reached ~0.3. Bacterial suspensions were centrifuged at 17,000 × *g* for 5 min, washed with sterile PBS, and resuspended in complete RPMI (cRPMI) medium without antibiotics, de-clumped by 10 passages through a 25-gauge needle, and diluted in cRPMI immediately before the infection.

**TABLE 1:** Laboratory and clinical MTB strains considered in this study and classification used for the analysis of the results^*e*^

Strain^*a*^	Lineage nomenclature	Grouping geo-host adaptation^*b*^	Lineage type	Virulence	Laboratory adaptation	Assay				
						Phagolysosomal acidification	Autophagy^*c*^	Apoptosis*^c^*	Macrophage survival	Mycobacterial survival
*M. africanum* 10,514/01^*d*^	6 (Africanum)	Specialist (SPEC)	Ancient	Less	Clinical	y	y	y	y	y
*M. africanum* 5,468/02^*d*^	6 (Africanum)	SPEC	Ancient	Less	Clinical	y	y	y	y	y
*M. bovis* BCG	Bovis	Other (OTH)	Animal	Less	Laboratory	y	y	n	y	n
*M. tuberculosis* 12,594/02^*d*^	2 (Beijing)	Generalist (GEN)	Modern	More	Clinical	y	y	y	y	y
*M. tuberculosis* 1,934/03^*d*^	2 (Beijing)	GEN	Modern	More	Clinical	y	y	y	y	y
*M. tuberculosis* CDC1551^*d*^	4	Intermediate (INT)	Modern	More	Clinical	y	n	n	y	n
*M. tuberculosis* 1,797/03^*d*^	1	SPEC	Ancient	Less	Clinical	y	n	n	n	n
*M. tuberculosis* 4,850/03^*d*^	1	SPEC	Ancient	Less	Clinical	y	n	n	n	n
*M. tuberculosis* H37Ra NCTC 7417	4	OTH	Modern	Less	Laboratory	y	y	n	y	y
*M. tuberculosis* H37Rv NCTC 7416	4	GEN	Modern	More	Laboratory	y	y	y	y	y

^*a*^ L1, L6, L2, and L4 clinical isolates from (17).

^*b*^ Grouping according to (8).

^*c*^ Provided as Files S4 and S5File S4 and S5.

^*d*^ Strains from (17).

^*e*^ Assay performed: y (yes); n (no).

For comparative infection experiments, we selected two representative strains each from L2, L3, and L6. Each of these strains was tested in at least two independent biological replicates, defined as separate experiments using independently grown bacterial cultures. In parallel, reference strains, including H37Rv, H37Ra, BCG, and CDC1551, were included as controls and tested across at least three independent experiments using separate cultures. As these strains are genetically identical across replicates, they are considered biological replicates of the same strain.

### Cells preparation and infection

Macrophage-like cells were derived from THP-1 as previously described (19). THP-1 cells were used for infection experiments at passages 7 to 14 to ensure consistent differentiation and functional responses. Briefly, THP-1 cells (ATCC TIB-202) were differentiated in cRPMI (with 2 mM L-glutamine, 10 mM HEPES, 100 nM non-essential amino acids, 1 mM sodium pyruvate, Sigma Aldrich, Missouri, USA) supplemented with 10% FBS (fetal bovine serum, Euroclone, Italy), antibiotics (100 U/mL penicillin G and 100 U/mL streptomycin sulfate, Sigma Aldrich, Missouri, USA), and 100 nM of phorbol 12-myristate 13-acetate (PMA, Sigma Aldrich, Missouri, USA) for 3 days in Eppendorf Cell Imaging 96-well plates (55,000 cells/well) with 170 µm thick glass bottoms (Eppendorf, Germany). Type I collagen (Sigma Aldrich, Missouri, USA) was used for improving cell adhesion on bottom glass surface, mimicking more physiological conditions, and improving both morphology and experimental consistency.

Given the lack of standardized protocols for recapitulating alveolar macrophages using *in vitro* cell lines, we adopted an experimental strategy based on two well-defined polarization conditions representing opposite extremes of the macrophage activation spectrum (hereafter referred to as M1-like and M2-like states). These conditions were used as simplified and reproducible reference states rather than as discrete or exhaustive representations of macrophage phenotypes. For simplicity, the terms M1 and M2 are used throughout the manuscript to denote these M1-like and M2-like activation states. Three days after PMA removal, macrophage-like cells were polarized in cRPMI medium without antibiotics for 16 h with either lipopolysaccharide (50 ng/mL, Sigma-Aldrich, Missouri, USA) to induce an M1-like phenotype [M(LPS)] or human IL-4 (20 ng/mL, *E. coli*-derived human IL-4 protein, R&D Systems, Minnesota, USA) to induce an M2a-like phenotype [M(IL-4)] prior to the infection (19–21). Effective polarization of cells was evaluated by checking morphology and the expression of specific markers for M1 and M2 (TNFα, IL-1β, CD80, IL-6, CD206) (Fig. S1). THP-1-derived macrophages were infected with mycobacteria at a theoretical multiplicity of infection (MOI) of 10:1 for achieving robust and efficient infection, ensuring that most target cells are exposed to bacteria. Infection was carried out by centrifugation (300 × *g* for 5 min, room temperature), followed by 2 h of incubation at 37°C and 5% CO_2_. After this 2-hour exposure period, extracellular mycobacteria were removed by washing. Macrophage-like cells were then washed twice, and fresh cRPMI without antibiotics was added. This washing step was applied to all infection conditions unless otherwise stated. cRPMI without phenol red was used in case the infection was set up for imaging readouts.

### Live imaging microscopy

All experiments were carried out using a Leica TCS SP5 confocal microscope equipped with an incubator chamber, allowing stable maintenance of cells at 37°C in 5% CO_2_. Images were acquired as 16-bit, 1024 × 1024 resolution, TIFF files using a 63×, 1.40 NA immersion oil objective.

#### Phagosome acidification, live imaging

To assess acidification dynamics in infected macrophages, we employed LysoSensor staining combined with confocal microscopy and *z-*stack acquisition. Intracellular acidification was quantified using a size-filtered ROI mask to identify LysoSensor-positive structures, thereby excluding background signal and capturing a defined range of acidic compartments (File S1). For clarity, these LysoSensor-positive structures are hereafter referred to as acidified compartments, encompassing large lysosomes as well as phagolysosomes. Throughout the manuscript, references to reduced or absent acidification reflect LysoSensor-based detection of acidic compartments and do not distinguish between impaired phagosomal acidification and potential cytosolic localization of MTB. To specifically assess phagosomal acidification, we performed colocalization analysis between LysoSensor fluorescence and internalized bacteria, allowing us to distinguish pathogen-associated acidified compartments from the broader lysosomal population (see File S1 for details). M1 and M2 macrophages were stained with Hoechst 33,342 NucBlue Live ReadyProbes Reagent (Ex/Em 360–460 nm, Thermo Fisher, Massachusetts, USA) for nuclei, and with LysoSensor Green DND-189 (Ex/Em 443–505 nm, pK_a_ 5.2, Thermo Fisher) for monitoring the dynamics of acidified compartments and phagosome acidification according to the manufacturer’s instructions. Imaging experiments were performed at 6 and 24 h p.i. For each strain, six images (two positions for each well out of three wells per strain) were acquired as *z-*stacks (0.89 µm × seven stacks) in at least two independent experiments. All images have been acquired and analyzed using the same settings, allowing quantitative comparison of LysoSensor intensities (File S2). Biological and technical replicates are detailed in File S2.

Induction of autophagosomes, apoptosis, and necrosis in live imaging is provided in File S4 and S5, respectively.

### Data analysis

Images were processed for single-cell analysis using Fiji software (22). Stacks have been analyzed by applying the maximum intensity projection method. For each infected well, at least five cells with internalized mycobacteria and five without internalized (unless not available) were considered for each acquisition. Infected wells were compared to “uninfected controls” (CTRL), defined as samples unexposed to mycobacterial infection. Effective cell infection was visually inspected using orthogonal views. Single cells were manually segmented (File S1).

All images have been acquired and analyzed using the settings reported in File S1. The Mann–Whitney test was performed to compare two independent groups, while, in the presence of more than two independent groups, the Kruskal–Wallis test followed by post hoc analysis using Dunn’s test was used. In the presence of dependent observations, linear/logistic mixed-effects models (23, 24), linear quantile mixed models (25), or zero-inflated mixed-effects Poisson models (26) were performed (File S1). Inferential techniques were applied in the presence of adequate sample sizes (*n* ≥ 5). For all analyses, the significance threshold was set at 0.05. Analyses were performed using R statistical software v.4.4.3 (27). Full statistics reports are available upon request.

### Macrophage survival

THP-1 cells were plated and differentiated in 96-well plates (55,000 cells/well) as described above (M1 or M2) and *in vitro* infected with MTB at a multiplicity of infection (MOI) of 5:1, selected to ensure efficient macrophage infection while minimizing excessive bacterial burden and infection-associated cytotoxicity. After 2 h of exposure, extracellular bacteria were removed by washing twice, and cells were cultured in fresh medium without antibiotics until the indicated time points. MTB strains used for this experiment include the laboratory strains H37Rv, H37Ra, *M. bovis* BCG NCTC 5692, CDC1551, and the clinical strains 2× L2 and 2× L6 (Table 1). After 48 h and 5 days p.i., cell viability was assessed using the crystal violet assay, as previously described (28) (File S1). In this context, macrophage viability was operationally defined as the persistence of adherent cells over time, measured by crystal violet staining, reflecting cumulative macrophage loss during infection rather than absolute live/dead cell counts. Data were expressed as the percentage of adherent cells relative to the uninfected controls.

### Intramacrophage MTB survival

For mycobacterial survival, macrophages were infected as described above, and after the removal of extracellular bacteria by washing, cells were lysed with sterile water at the indicated time points. Supernatants and cell lysates were serially diluted, and each dilution was plated on 7H10 agar plate to count MTB CFUs present at 4 h and 48 h p.i. After 3 weeks of incubation, colonies were counted. CFUs/mL obtained at 4 h p.i., representing macrophage-associated bacteria (entry), were normalized to CFUs obtained from the inoculum, while CFUs obtained at 24 and 48 h p.i. were normalized to 4 h p.i. to assess intracellular survival. Analyses were performed using Prism (GraphPad) software, version 9.1.

## RESULTS

The activity of acidifying drugs, when used jointly with antibiotics, impairs MTB survival (29). Phagosome acidifying mechanisms are cell-autonomous and therefore strictly dependent on the phenotype of both the host and the infecting MTB strain, as well as their interactions. Therefore, we aimed to assess the ability of different strains to limit or evade acidification-associated responses in both M1 and M2 macrophages. LysoSensor Green stains acidic compartments broadly; therefore, we refer to them as “acidified compartments” throughout. To identify pathogen-containing acidified compartments, we used colocalization between LysoSensor signal and internalized bacteria. This colocalization does not necessarily prove canonical phagolysosome identity, nor does it discriminate between bacteria residing in non-acidified compartments and those that may have accessed the cytosol (see Materials and Methods and Discussion).

Findings are described using the grouping generalists/specialists, as this classification encompasses different features, such as phylogeny, geographical distribution, and host adaptation. Phagosome acidification results for ungrouped samples are reported in File S2.

### Strain- and polarization-dependent phagosomal acidification reveals heterogeneous macrophage responses to MTB

**Fig 1:**
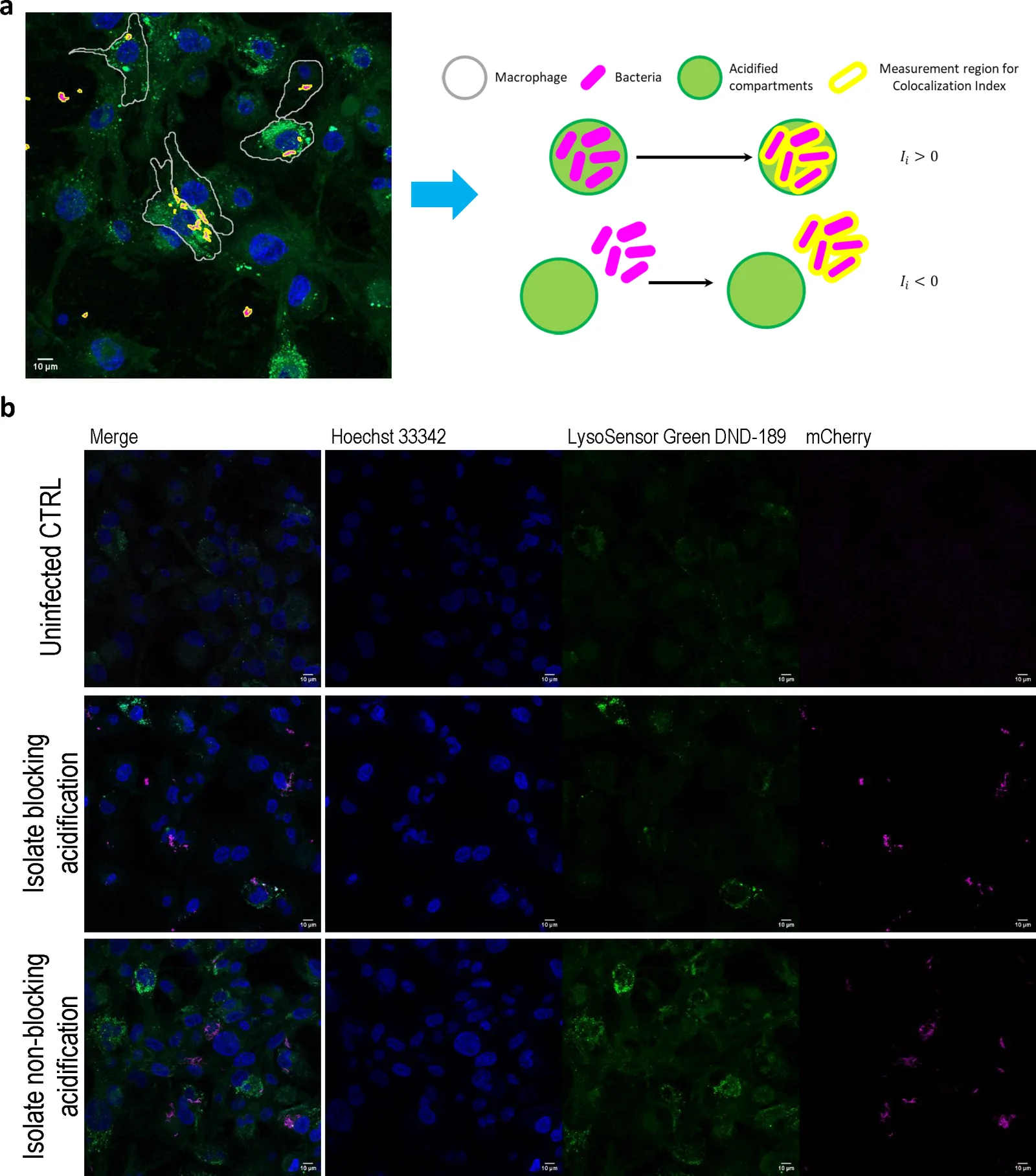
Phagosomal acidification in macrophages. (**a**) Schematic representation of the elements considered for the analysis (see section “Images and data analysis - Phagolysosomal acidification” in File S1 or details). (**b**) Representative confocal images illustrating the experimental readout: uninfected control macrophages (no exposure to mycobacteria, top), macrophages infected with an isolate associated with reduced LysoSensor-positive signal (middle), and macrophages infected with an isolate associated with preserved LysoSensor-positive signal (bottom). Live-cell fluorescence imaging was performed after staining with LysoSensor (green), which reports acidified intracellular compartments, and Hoechst (blue) for nuclei. Bacteria are shown in magenta (mCherry). The images shown are illustrative examples of the phenotypic categories used for the quantitative analyses presented in Fig. 2 to 4. Representative images for all strains, cell types, and time points analyzed are provided in File S7. Scale bar: 10 µm.

LysoSensor Green was used to quantify the abundance and intensity of acidified compartments in THP-1-derived M1 and M2 macrophages infected with generalist (GEN), intermediate (INT), specialist (SPEC), and other (OTH) strains (Table 1) at 6 and 24 h p.i. (Fig. 1). To specifically assess phagosomal acidification, LysoSensor fluorescence was analyzed in conjunction with intracellular bacterial localization. A total of 7,384 single cells (4,098 at 6 h p.i.; 3,286 at 24 h p.i.) are considered for the analysis (on average, for each strain tested, M1: 178 ± 67 uninfected cells + 172 ± 44 infected cells; M2: 180 ± 65 uninfected cells + 174 ± 46 infected cells) (File S2).

**Fig 2:**
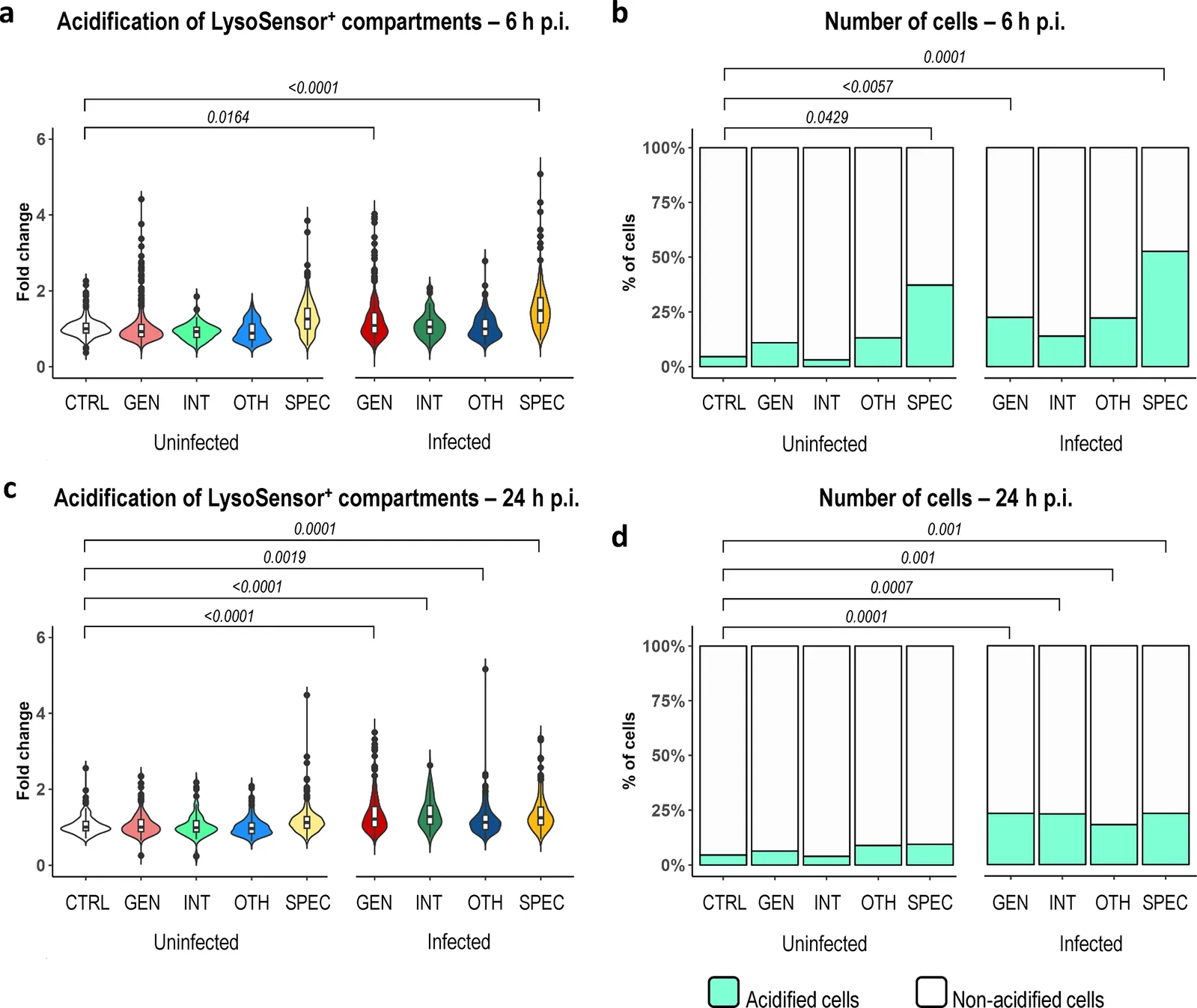
Phagosomal acidification in M1 cells. Phagosomal acidification at 6 h p.i. (**a**) and at 24 h p.i. (**c**). Fold changes are visualized as a violin plot to show the distribution, with a superimposed whisker plot (box plot) representing the median, interquartile range, and potential outliers. Data are normalized to M1 THP-1 macrophage uninfected controls, and strains are grouped as previously reported in Table 1. Linear mixed-effects models followed by post hoc analysis. Percentage of cells showing phagosomal acidification at 6 h p.i. (**b**) and at 24 h p.i. (**d**) is also reported. Cells were classified as acidified when their per-cell phagosomal acidification metric, derived from LysoSensor-positive compartment signal, exceeded the upper control limit (mean + 1.96 SD) defined from uninfected THP-1 macrophage controls (see File S1 for details). Logistic mixed-effects models followed by post hoc analysis. GEN: Generalist strains *M. tuberculosis* 12,594/02 (L2) (17), *M. tuberculosis* 1934/03 (L2) (17), *M. tuberculosis* H37Rv NCTC 7416 (L4); INT: Intermediate strain *M. tuberculosis* CDC1551 (17); OTH: Other strains *M. tuberculosis* H37Ra NCTC 7417, *M. bovis* BCG; SPEC: Specialist strains *M. africanum* 10,514/01 (L6) (17), *M. africanum* 5468/02 (L6) (17), *M. tuberculosis* 1797/03 (L1) (17), *M. tuberculosis* 4850/03 (L1) (17). *P*-values: comparison between uninfected controls and each category. Uninfected CTRL, no exposure to mycobacteria; uninfected GEN/INT/OTH/SPEC, exposure to mycobacteria but no uptake observed; infected, intracellular mycobacteria observed. Statistical annotations shown in the panels reflect these analyses; only comparisons relevant to the primary findings are discussed in the Results section, while additional comparisons are provided for completeness. Secondary analysis comparing M1 vs M2 is available in File S3. Representative raw confocal images corresponding to the conditions analyzed are provided in the File S7 to illustrate variability underlying the quantitative analyses.

**Fig 3:**
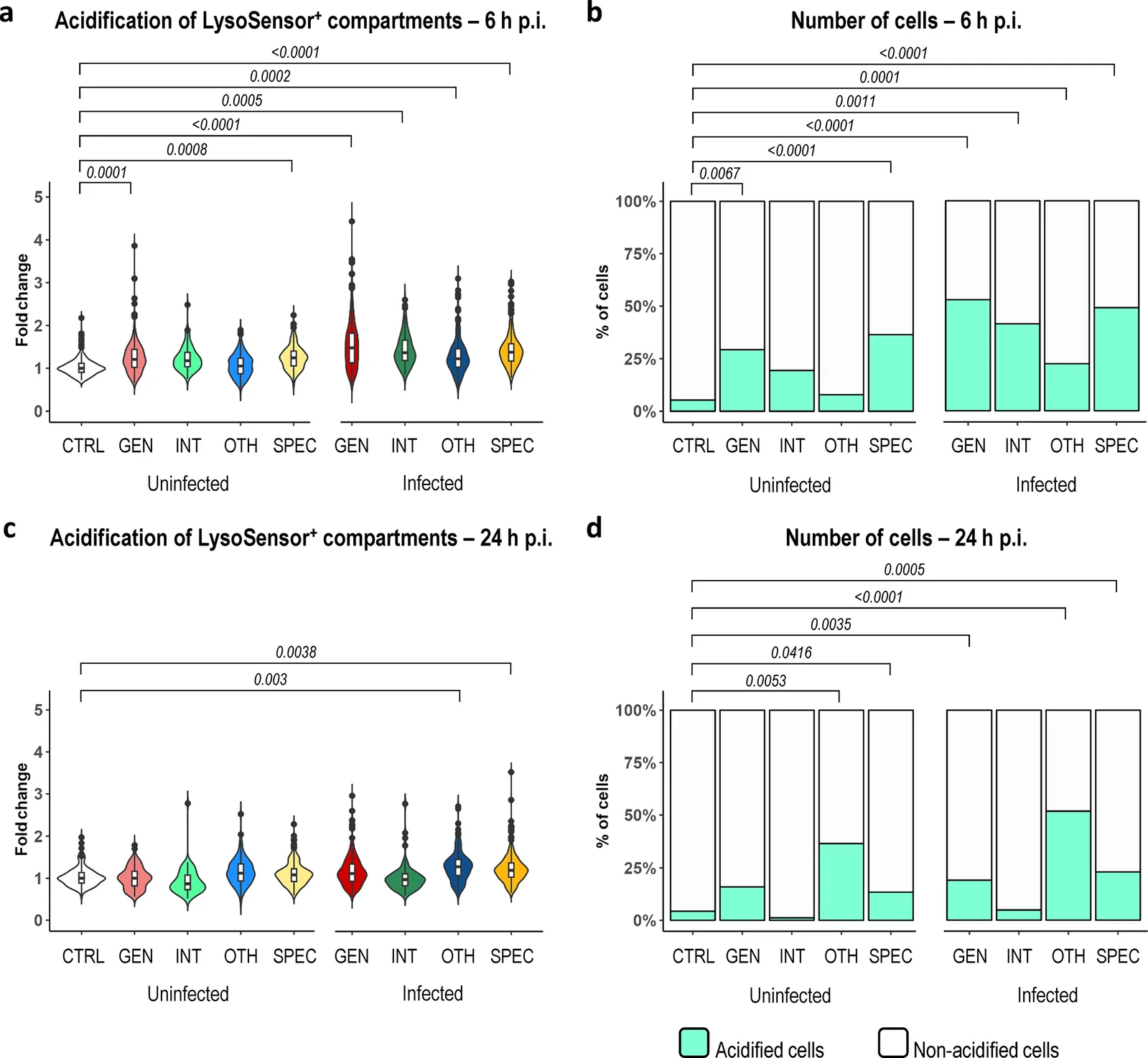
Phagosomal acidification in M2 cells. Phagosomal acidification at 6 h p.i. (**a**) and at 24 h p.i. (**c**). Fold changes are visualized as a violin plot to show the distribution, with a superimposed whisker plot (box plot) representing the median, interquartile range, and potential outliers. Data are normalized on M2 THP-1 macrophages uninfected controls (CTRL), and strains are grouped as previously reported in Table 1. Linear mixed-effects models followed by post hoc analysis. Percentage of cells showing phagosomal acidification at 6 h p.i. (**b**) and at 24 h p.i. (**d**) is also reported. Cells were classified as acidified when their per-cell phagosomal acidification metric, derived from LysoSensor-positive compartment signal, exceeded the upper control limit (mean + 1.96 SD) defined from uninfected THP-1 macrophage controls (see File S1 for details). Logistic mixed-effects models followed by post hoc analysis. GEN: Generalist strains *M. tuberculosis* 12,594/02 (L2) (17), *M. tuberculosis* 1934/03 (L2) (17), *M. tuberculosis* H37Rv NCTC 7416 (L4); INT: Intermediate strain *M. tuberculosis* CDC1551 (17); OTH: Other strains *M. tuberculosis* H37Ra NCTC 7417, *M. bovis* BCG; SPEC: Specialist strains *M. africanum* 10,514/01 (L6) (17), *M. africanum* 5468/02 (L6) (17), *M. tuberculosis* 1797/03 (L1) (17), *M. tuberculosis* 4850/03 (L1) (17). *P*-values: comparison between uninfected controls and each category. Uninfected CTRL, no exposure to mycobacteria; uninfected GEN/INT/OTH/SPEC, exposure to mycobacteria but no uptake observed; infected, intracellular mycobacteria observed. Statistical annotations shown in the panels reflect these analyses; only comparisons relevant to the primary findings are discussed in the Results section, while additional comparisons are provided for completeness. Secondary analysis comparing M1 vs M2 is available in File S3. Representative raw confocal images corresponding to the conditions analyzed are provided in the File S7 to illustrate variability underlying the quantitative analyses.

**Fig 4:**
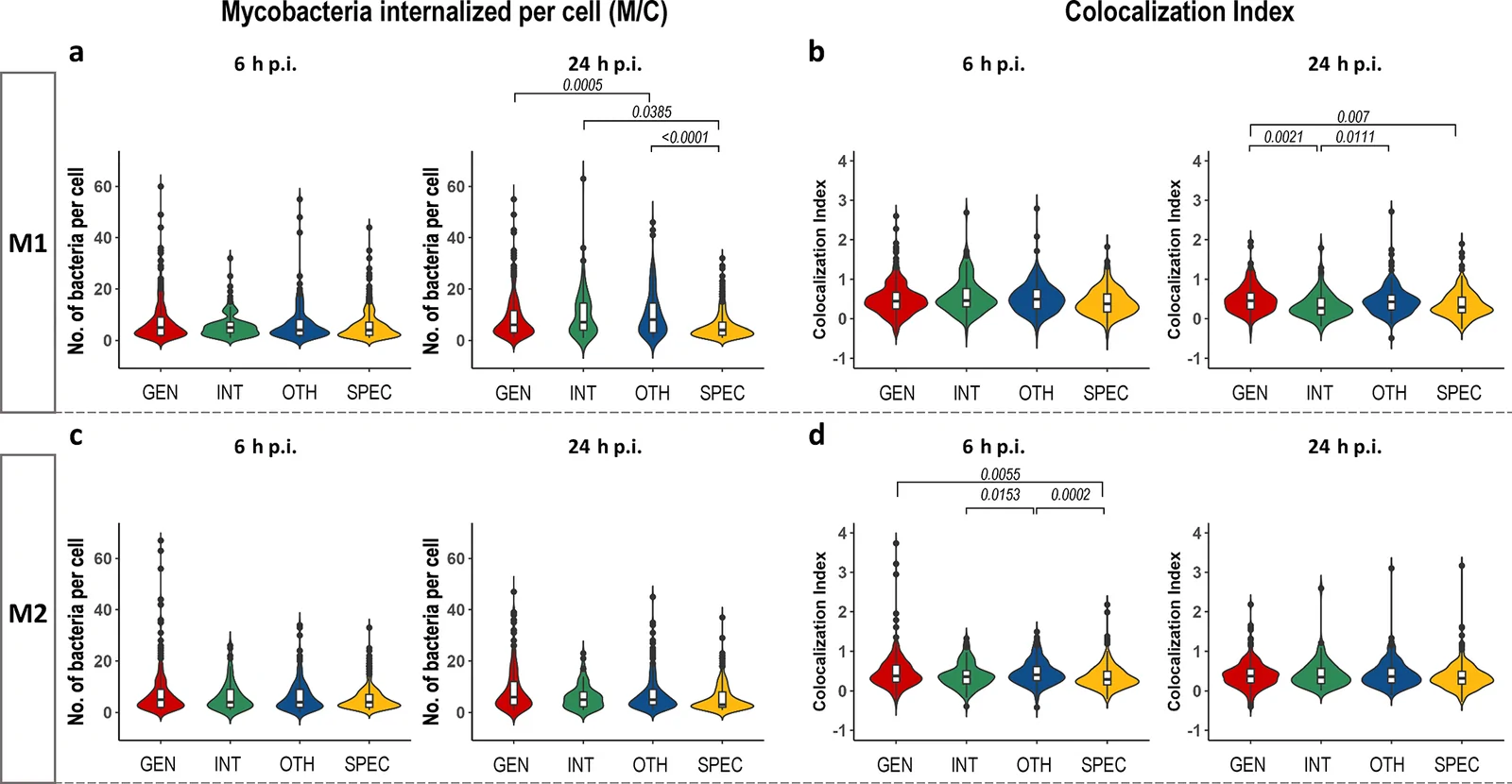
(**a**) Effective number of mycobacteria internalized per cell (M/C) at 6 h p.i. and at 24 h p.i. in M1 cells. (**b**) MTB colocalization with acidified compartments at 6 h p.i. and 24 h p.i. in M1 cells. (**c**) Effective number of mycobacteria internalized per cell (M/C) at 6 h p.i. and at 24 h p.i. in M2 cells. (**d**) MTB colocalization with acidified compartments at 6 h p.i. and 24 h p.i. in M2 cells. Linear mixed-effects models followed by post hoc analysis. Statistical annotations shown in the panels reflect these analyses; only comparisons relevant to the primary findings are discussed in the Results section, while additional comparisons are provided for completeness. GEN: Generalist strains *M. tuberculosis* 12,594/02 (L2) (17), *M. tuberculosis* 1934/03 (L2) (17), *M. tuberculosis* H37Rv NCTC 7416 (L4); INT: Intermediate strain *M. tuberculosis* CDC1551 (17); OTH: Other strains *M. tuberculosis* H37Ra NCTC 7417, *M. bovis* BCG; SPEC: Specialist strains *M. africanum* 10,514/01 (L6) (17), *M. africanum* 5468/02 (L6) (17), *M. tuberculosis* 1797/03 (L1) (17), *M. tuberculosis* 4850/03 (L1) (17). Secondary analysis comparing M1 vs M2 is available in File S3. Representative raw confocal images corresponding to the conditions analyzed are provided in the File S7 to illustrate variability underlying the quantitative analyses.

The bystander effect refers to a phenomenon in which uninfected cells exhibit measurable changes in response to signals or factors originating from nearby infected cells. In this study, bystander macrophages were operationally defined as uninfected cells present within the same microscopic field of view as infected macrophages within a given well. For each confocal image, bystander cells were selected among LysoSensor-stained, bacteria-negative macrophages imaged simultaneously with infected cells. No explicit distance-based threshold was applied. Thus, spatial proximity was defined by co-localization within the same imaged field rather than by direct cell–cell contact. This means that even though some cells are not directly infected by MTB, they still show similar features as the infected cells because of their proximity or response to the influence of infected cells. In M1 macrophages, GEN and SPEC strains do not show a reduction in LysoSensor-based acidification signal after 6 h p.i., with the latter inducing also a bystander effect (an increase in acidification of uninfected macrophages imaged within the same field of view), although this effect is not statistically significant (Fig. 2a). In contrast, the categories INT and OTH display a reduction of acidification levels in large LysoSensor-positive compartments, suggesting interference with host endolysosomal dynamics (Fig. 2a). On the contrary, the SPEC strains at 6 h p.i. show the least reduction in acidification-associated signals and induce the highest number of acidified macrophages (50% vs <25% of all the other categories) (Fig. 2b). At 24 h p.i., acidification levels are overall high, irrespective of the infecting MTB lineage (Fig. 2c). However, at the single-cell level, only a small macrophage fraction displays such acidification (<25%) (Fig. 2d). Analysis of the number of LysoSensor-positive compartments revealed a time-dependent shift in M1 macrophages infected with GEN versus SPEC strains. At 6 h p.i., GEN-infected macrophages displayed significantly fewer acidified compartments than SPEC, whereas at 24 h p.i., GEN-infected macrophages displayed significantly more acidified compartments, indicating a group-specific temporal modulation of phagosomal acidification (File S2). In M2 macrophages, none of the categories show a reduction in LysoSensor-positive compartments at 6 h p.i. (Fig. 3a), and the fraction of acidified macrophage ranges between 25% (OTH) and 50% (SPEC and GEN) (Fig. 3b). The SPEC strains induce a bystander effect in uninfected M2 macrophages (approximately 40% of the SPEC uninfected cells show acidification; Fig. 3a and b). As the time of infection progresses (24 h p.i.), GEN and INT strains are associated with a marked reduction in the acidification of compartments (Fig. 3C), and the fraction of acidified macrophages decreases below 25%, except for the OTH category (50%) (Fig. 3D). No significant differences were observed in the number of LysoSensor-positive compartments across the different MTB groups (File S2).

We then evaluated the replicative potential of the MTB strains by assessing their killing in M1 and M2 macrophages, expressed as the number of mycobacteria internalized per cell (M/C).

All the strains infect M1 macrophages with comparable efficiency, as M/C values at 6 h p.i. are not significantly different (Fig. 4a). MTB colocalization inside acidified compartments is similar among all the groups (Fig. 4b). Comparison of M/C values at 6 h p.i. vs 24 h p.i. within the same group highlights some differences in proliferation: GEN, INT, and OTH show increased M/C at 24 h p.i. (*P*-values 0.0139, 0.0011, and <0.0001, respectively), whereas SPEC strains display no significant differences between the time points considered (*P*-value 0.7748) (File S3). SPEC strains show the lowest M/C values compared to the other groups, suggesting an impaired proliferation or a higher killing (Fig. 4a). The MTB-phagosome colocalization index within acidified compartments is lower for SPEC and INT strains compared to the GEN strains (Fig. 4b).

In M2 macrophages, all the strains infect and survive with similar rates, even though GEN strains display a heavy-tail distribution due to cells with very high M/C values (Fig. 4c). At 6 h p.i., SPEC strains showed a significantly reduced colocalization with acidified compartments compared to GEN strains, whereas INT strains displayed an intermediate distribution that did not reach statistical significance relative to GEN (Fig. 4d). These differences were no longer detectable at 24 h p.i. (Fig. 4d). Comparison of M/C values at 6 h p.i. vs 24 h p.i. within the same group highlights differences in proliferation only for GEN and OTH (*P*-values 0.001 and 0.0442, respectively), whereas SPEC and INT strains display no significant differences between the time points considered (*P*-values 0.1501 and 0.6585, respectively) (File S3). No correlation between M/C and the colocalization index was found for the described time points (data not shown).

Together, these findings indicate that the ability of phagosome acidification to halt MTB growth is triggered by all the strains in M2 macrophages soon after infection and becomes unpaired in GEN- and INT-infected cells upon infection progression. Conversely, in M1 macrophages, complete phagosome acidification occurs later but with greater efficiency. This enhanced efficiency is observed across all the isolates examined. Nonetheless, the single-cell approach revealed the heterogeneity of the response in the macrophage population, indicating that only 25% of infected cells is still capable of eliciting phagosomal acidification and, possibly, protecting the cells. Furthermore, SPEC isolates induce a bystander effect in uninfected macrophages. As suggested by the analysis of the number of LysoSensor-positive compartments (File S2), the bystander increase in acidification in uninfected macrophages appears to reflect a true enhancement of phagosomal acidification per compartment rather than an increase in the number of compartments. It is worth mentioning that M1 and M2 macrophages show different acidification response timings to MTB infections, suggesting an intrinsic flexibility and adaptability of their protective mechanisms.

To further investigate the heterogeneity of macrophage responses, we applied linear quantile mixed models (LQMM) and logistic mixed-effects models to perform comparison between M1 and M2 (File S3). LQMM showed no significant differences in median acidification values between M1 and M2, indicating similar overall levels. In contrast, logistic mixed-effects models identified three significant differences: (i) the percentage of cells showing acidification, (ii) the colocalization index at 6 h p.i., and (iii) the number of mycobacteria per cell at 24 h p.i. These findings suggest that, while median acidification is comparable, the responses of individual cells differ in terms of the proportion of responders, timing, and infection outcome.

Group-specific analyses revealed that these differences are primarily driven by the GEN (non-infected and infected at 6 h p.i.) and the INT (infected at 24 h p.i.) groups, whereas other macrophage subgroups showed comparable responses (File S3).

### MTB infection induces limited autophagic maturation and polarization-dependent apoptotic responses

Although our primary focus was on acidification, we also explored additional host responses, including autophagy, apoptosis, and necrosis, which are provided as Files S4 and S5, respectively. Briefly, none of the strain groups induced effective autophagy: although autophagosome number increased, their size did not show a significant enlargement, suggesting impaired maturation and limited progression of the autophagic flux (File S4). This pattern was consistent in both M1 and M2 macrophages. Apoptosis was more pronounced in M1 macrophages, particularly in bystander non-infected cells exposed to GEN and OTH strains (~40%). A similar, though not statistically significant, trend was observed in infected cells. In M2 macrophages, only bystander cells exposed to OTH strains showed a modest, non-significant increase in apoptosis (~30%) (File S5).

### MTB strain virulence and macrophage polarization differentially impact macrophage viability over time

**Fig 5:**
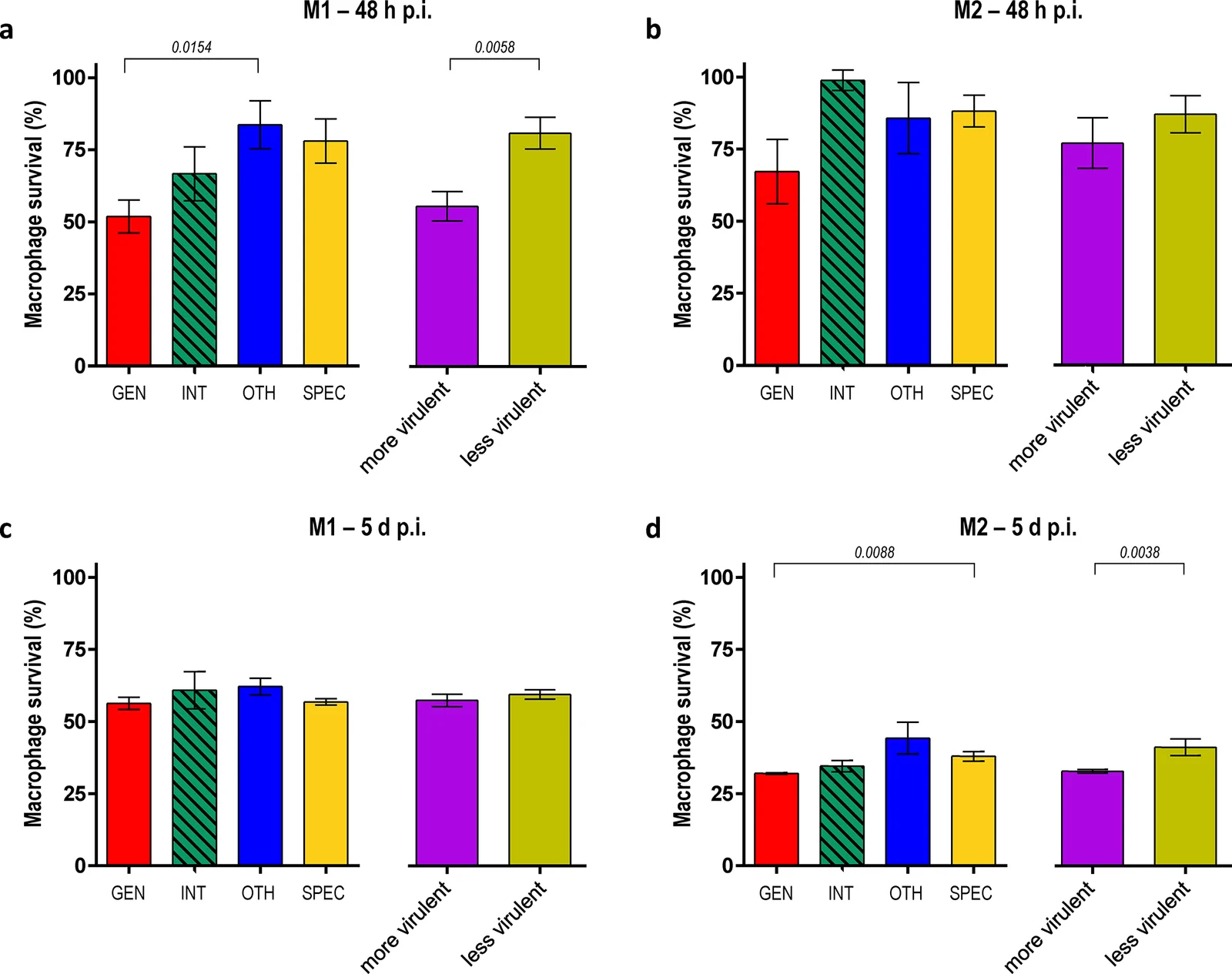
Relative macrophage viability at 48 h and 5 days post-infection. Relative macrophage viability at 48 h and 5 days post-infection, assessed by crystal violet staining. Percentage of adherent M1 macrophages at 48 h (**a**) and 5 days (**c**) post-infection and adherent M2 macrophages at 48 h (**b**) and 5 days (**d**) post-infection. Data (mean ± standard error of the mean) are expressed as the percentage of adherent cells relative to uninfected control macrophages, which were set to 100%. In this assay, macrophage viability is operationally defined as the persistence of adherent, structurally intact cells over time and reflects cumulative macrophage loss during infection rather than absolute live/dead cell counts. Strains are grouped as previously reported in Table 1. Kruskal–Wallis and Mann–Whitney tests. INT group is reported for comparison but excluded from the statistical model (*n* < 5, see Materials and Methods). GEN: Generalist strains *M. tuberculosis* 12,594/02 (L2) (17), *M. tuberculosis* 1934/03 (L2) (17), *M. tuberculosis* H37Rv NCTC 7416 (L4); INT: Intermediate strain *M. tuberculosis* CDC1551 (17); OTH: Other strains *M. tuberculosis* H37Ra NCTC 7417, *M. bovis* BCG; SPEC: Specialist strains *M. africanum* 10,514/01 (L6) (17), *M. africanum* 5468/02 (L6) (17), *M. tuberculosis* 1797/03 (L1) (17), *M. tuberculosis* 4850/03 (L1) (17).

To quantify the impact of MTB infections on macrophage survival, we compared cell viability after infection with that of uninfected cells. Figure 5a shows viability data at 48 h p.i., indicating that some strains decrease viability by 20%, whereas others decrease it by 50% in M1 cells. At the same time point, all the strains are less aggressive on M2 cells (Fig. 5b). As the infection progresses for 5 days, M1 cell viability for all the infected populations is 50% compared to the uninfected populations (Fig. 5c). Conversely, M2 macrophages show a dramatic viability decrease (80%) for all the infecting MTB categories (Fig. 5d).

By clustering the tested strains according to their virulence (see Table 1), it is noticeable that highly virulent strains reduce M1 cell viability by 50% within 48 h p.i., while low-virulence strains are less effective at killing M1 macrophages even at 5 days p.i. However, this difference in virulence appears mitigated in M2 cells. Highly virulent strains reduce M2 cell viability by 80% between 3 and 5 days, while low-virulence strains manage to kill nearly 70% of M2 cells by 5 days p.i.

### Clinically adapted MTB strains display enhanced intracellular replication, particularly in M2 macrophages

Colony-forming unit (CFU) assays were performed to quantify bacterial entry into M1 and M2 macrophages at 4 h p.i. and the intracellular bacterial burden at 48 h p.i. Macrophages were exposed to a constant number of MTB cells, followed by removal of extracellular bacteria by washing. At 4 h and 48 h, cells were lysed, and intracellular MTB was recovered for CFU enumeration. CFU values are reported as log CFU/mL, allowing direct comparison of intracellular bacterial loads between strains and across time points (File S6). Uptake relative to the initial inoculum, reflecting entry efficiency, is shown separately (File S6).

At 4 h p.i., intracellular CFU counts were consistently higher in M2 macrophages compared to M1 macrophages across all MTB strains (File S6). Among laboratory strains, H37Ra exhibited higher intracellular CFU levels than H37Rv, whereas H37Rv and L6 strains showed comparable intracellular burdens. In contrast, L2 strains displayed lower CFU counts at 4 h p.i., consistent with their reduced uptake relative to the inoculum (File S6).

**Fig 6:**
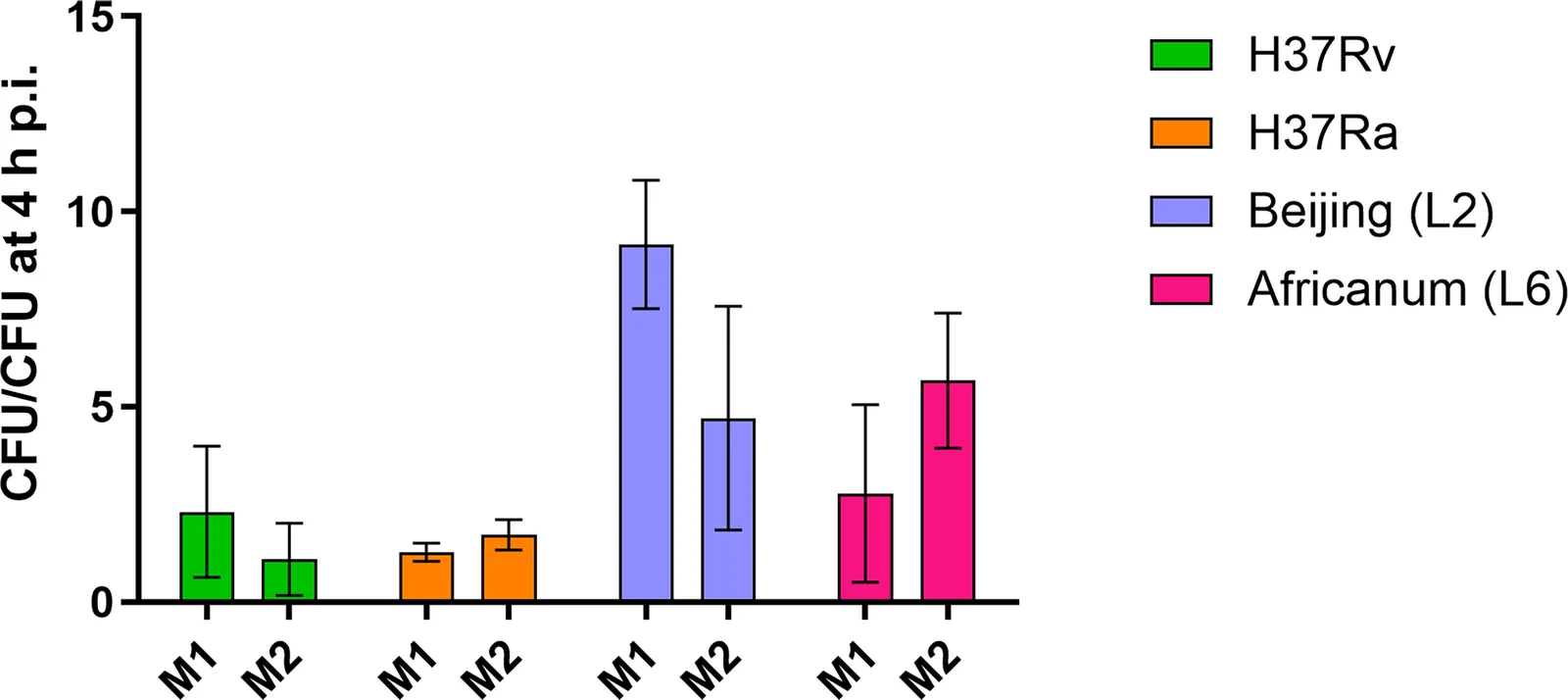
MTB intramacrophage survival at 48 h post-infection. CFU results at 48 h p.i. Data (mean ± standard error of the mean) are normalized on CFU at 4 h p.i. Beijing: GEN/L2 (*M. tuberculosis* 12,594/02 [17], *M. tuberculosis* 1934/03 [17]); Africanum: SPEC/L6 (*M. africanum* 10,514/01 [17], *M. africanum* 5468/02 [17]). Statistical model not applied (*n* < 5, see Materials and Methods).

At 48 h p.i., intracellular CFU counts increased for all strains in both macrophage phenotypes (Fig. 6). Notably, despite their lower initial uptake, L2 strains reached intracellular CFU levels comparable to other lineages at 48 h p.i., consistent with a higher intracellular replication capacity.

## DISCUSSION

Our single-cell–based study shows that both MTB lineages and macrophage differentiation status strongly influence the cell-autonomous defensive mechanisms deployed during MTB infection. To our knowledge, our study is among the first to integrate both host immunophenotype heterogeneity and *M. tuberculosis* genetic diversity within a single experimental setting. The use of single-cell–based analyses represents an additional novelty relevant to comprehend the heterogeneity of the host-pathogen interaction.

On a mechanistic level, our results contribute to a better understanding of MTB survival within macrophages, pathogenesis, and virulence. At a translational level, our findings provide a conceptual framework for considering how MTB genetic diversity and macrophage activation states may influence host pathways targeted by host-directed therapies (30). While the magnitude of differences in our single-cell data appears small, such shifts can still have significant biological implications. In single-cell studies, even subtle changes, such as early cellular transitions and rare subpopulation dynamics, may reflect downstream amplification effects. In this context, our study represents a starting point for exploring how macrophage and MTB heterogeneity could be integrated into the design and evaluation of host-directed therapeutic strategies, which will require validation in primary cells and *in vivo* models.

### MTB lineages calibrate phagosome acidification response unique to macrophage phenotypes

An important contribution of phagosomal acidification is highlighted by the fact that most permissive monocytes display lower lysosome content, reduced acidification, and decreased proteolytic activity, which enable MTB *in vivo* persistence (31, 32). Recently, Bedard et al. contributed to the field by describing how lysosomal failure is critical for MTB (H37Rv) survival and how drugs that restore lysosomal function can counteract MTB survival (33).

**Fig 7:**
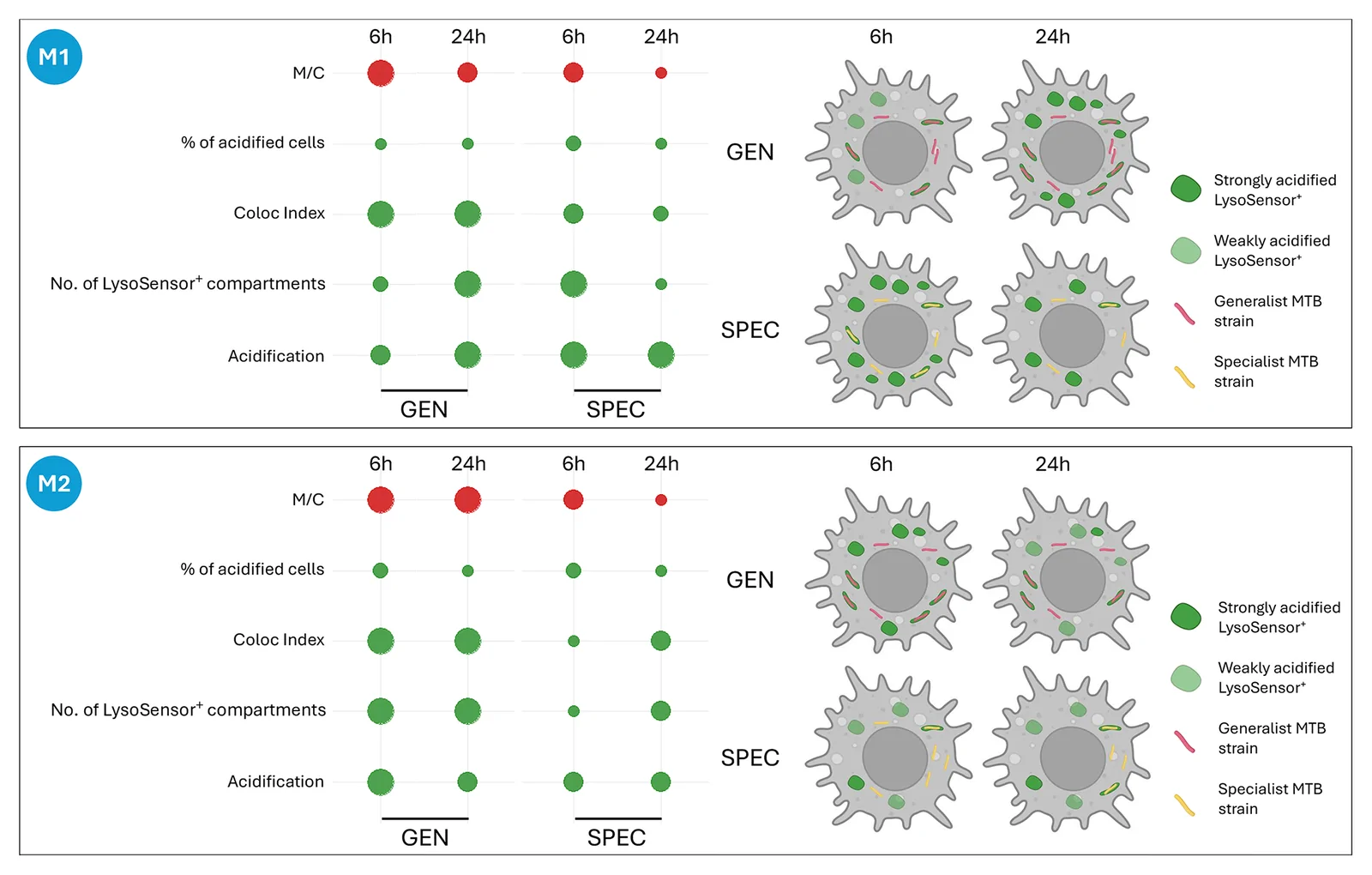
Temporal dynamics of phagosomal acidification in macrophages infected with MTB strains. Left: Bubble plots summarizing key variables at 6 h and 24 h post-infection (p.i.) across genetically diverse MTB strain categories (GEN, generalists; SPEC, specialists). Variables include the number of LysoSensor-positive compartments, acidification levels, colocalization of MTB with acidified compartments, percentage of responding cells, and bacillary load (M/C, mycobacteria per cell). Bubble size reflects relative magnitude. Right: Schematic representations of infected macrophages at 6 h and 24 h p.i., illustrating trends observed in the bubble plots, including differences in phagosomal acidification, bacterial colocalization with acidified vesicles, and bacillary burden between GEN and SPEC strains. Top panel: M1 macrophages. Bottom panel: M2 macrophages. GEN strains: *M. tuberculosis* 12,594/02 (L2), *M. tuberculosis* 1934/03 (L2), *M. tuberculosis* H37Rv NCTC 7416 (L4). SPEC strains: *M. africanum* 10,514/01 (L6), *M. africanum* 5468/02 (L6), *M. tuberculosis* 1797/03 (L1), *M. tuberculosis* 4850/03 (L1). Created in BioRender.

Although M1 macrophages are generally considered more antimicrobial than M2 macrophages, our single-cell analyses indicate that differences between macrophage phenotypes are primarily reflected in the timing, efficiency, and proportion of responding cells across multiple functional readouts—including phagosomal acidification, intracellular bacterial burden, and host cell survival—rather than in uniform population-wide effects. Our study provides evidence that different MTB lineages cope with phagosomal acidification in macrophages in lineage- and phenotype-specific ways, depending on the macrophage phenotype (illustrated in Fig. 7). In M1 macrophages, the higher level of acidification, together with the concomitant higher colocalization of modern, virulent GEN/INT isolates, suggests better tolerance to the acidic environment. This interpretation is further supported by our observation that, in M1 macrophages, GEN strains transiently limit early phagosomal acidification but ultimately sustain a larger pool of acidified compartments per cell, providing an expanded interface for host-pathogen interactions. In contrast, SPEC strains retain more acidified compartments early on but show no further increase, suggesting a distinct intracellular survival strategy. Consistent with this view, L2 strains evolved specific strategies to cope with acidification during intramacrophage infection (34–37). In contrast, L1, L5, and L6 (*M. africanum*) isolates harbor mutations that negatively affect the adaptive response to acidic pH and lysosomal delivery (38, 39). Despite recent findings showing no significant differences in mycobacterial survival of modern and L1 isolates in acidic pH *in vitro*, the transcriptional regulatory networks of ancient and modern lineages are clearly distinct, with crucial implications for stress responses *in vivo* (35).

Differently, the less robust M2 macrophage antimicrobial defenses are more readily permissive to modern, virulent GEN strains, which are associated with a pronounced reduction in detectable phagosomal acidification. Whereas ancient, less virulent SPEC strains seem not to affect phagosomal acidification itself, colocalization analysis at the time points considered suggests reduced association with acidified compartments, potentially reflecting alternative intracellular localization strategies (40). Consistent with this view, we observed no significant differences in the number of LysoSensor-positive compartments across MTB groups, indicating that phagosomal acidification per cell is comparable in this macrophage phenotype. It is well known that less virulent strains trigger an apoptotic bystander effect in uninfected M2 macrophages (41, 42). For the first time, we report a similar bystander effect, with increased acidification in M2 macrophages, underscoring the critical role of macrophage polarization in conditioning the microenvironment (43). Our observation that uninfected bystander macrophages show increased acidification suggests a functional amplification of host defense extending beyond directly infected cells. This response may be mediated by local signaling from infected macrophages, priming neighboring cells for more efficient antigen processing and antimicrobial activity. By enhancing the processing of secreted mycobacterial antigens and facilitating antigen presentation, such a mechanism could strengthen the overall capacity of the macrophage population to control infection. Furthermore, even if the infection prompts phagosomal acidification, the number of M1 or M2 macrophages triggering the signal remains relatively low.

### Macrophage phenotype is associated with differences in cell survival and MTB intracellular replication

We assessed the strains’ ability to enter and replicate in host cells, as well as kill macrophages, as an extreme measure of virulence (considered the “ability to cause damage”). It is important to note that bacterial replication and macrophage survival were assessed using population-level (bulk) measurements. While the crystal violet assay does not provide single-cell viability information, it offers a robust readout of cumulative macrophage loss during prolonged MTB infection and therefore complements the imaging-based single-cell analyses presented elsewhere in this study. Accordingly, these bulk readouts cannot be directly mapped onto the acidification state of individual cells observed by single-cell imaging. Instead, they reflect the integrated behavior of heterogeneous cellular subpopulations within the infected macrophage population. Compared to SPEC strains, GEN strains showed higher replicative potential in both M1 and M2 macrophages. Furthermore, M/C data trends also suggest an increased uptake of GEN strains in M2 macrophages. Survival rate strongly depends on the strain-specific virulence in M1 cells, whereas M2 cells are inherently more sensitive to the infection *per se* with no contribution of strain identity.

These findings are aligned with the microscopy observations for apoptosis, where GENs/more virulent strains induced higher cell death, mainly in M1 macrophages. Of note, M2 macrophages are more permissive to MTB growth compared to M1, an outcome partially due to the lower MTB intake by M1 macrophages in comparison with M2 and consistent with a reduced probability of mounting sustained restrictive responses rather than a complete inability to engage antimicrobial mechanisms. Based on these results, we may imagine that, in an *in vivo* scenario, the interplay between the specific MTB lineage and the immune environment, where all the other immune cells are included (i.e., T cells, natural killer cells, MAIT cells), as well as potential coinfection or comorbidities, may have a substantial impact on the immune response, containment of the mycobacteria, and the clinical outcome.

Suitable models of alveolar macrophages to dissect host-pathogen interactions in TB are currently missing, as commonly used models such as the Max Planck Institute mouse alveolar macrophages or human peripheral blood mononuclear cell-derived macrophages are poorly validated for this purpose (44, 45). We used PMA-differentiated THP-1 cells to reduce donor-to-donor variability inherent in primary cell models (46–48). In addition, by favoring a “pre-programming” rather than a “reprogramming” approach, we intentionally focused on defined macrophage activation states, while acknowledging that this strategy does not recapitulate the full complexity introduced by tissue niche-specific cues and macrophage ontogeny. Although macrophage phenotypes *in vivo* exist along a continuum, the use of standardized M1-like and M2-like conditions enabled controlled comparisons across MTB strains and macrophage activation extremes (49–52). Increasing the number of MTB isolates considered might be also needed. However, THP-1 derived macrophages do not fully recapitulate human alveolar macrophage physiology. Therefore, while THP-1 cells provide an internally consistent platform for comparing MTB lineages, extrapolation to alveolar macrophages *in vivo* should be made with caution and will require confirmation in primary human alveolar macrophages or *in vivo* models. Acidified compartments were identified using LysoSensor, which reports acidification of membrane-bound compartments but does not allow discrimination between bacteria residing in non-acidified phagosomes and those that may have accessed the cytosol. Cytosolic localization of MTB has been reported and may vary across strains, potentially contributing to the heterogeneous acidification patterns observed here. In this context, pharmacological modulation of vacuolar acidification (e.g., using V-ATPase inhibitors such as bafilomycin A1) could provide complementary functional validation of acidification-dependent effects but was not incorporated here due to the focus on live-cell imaging-based tracking of acidic compartments. Future studies incorporating organelle-specific markers (e.g., LAMP1), lysosomal biogenesis markers, and functional perturbation assays would provide additional resolution on the identity of MTB-containing compartments and help distinguish whether the observed changes reflect altered lysosomal number, compartmental acidification, or cytosolic access. Accordingly, our data reflect the net outcome of phagosomal acidification dynamics at the single-cell level rather than a definitive assessment of bacterial subcellular localization. While CYTO-ID staining provides an overview of autophagic vesicle accumulation, it does not allow mechanistic dissection of the autophagic process. As such, our data indicate no evidence of completed autophagic flux based on CYTO-ID, but further analyses (e.g., LC3/p62 turnover assays, with or without bafilomycin) would be required to define the underlying mechanisms. Finally, we did not test the effect of drugs, such as metformin, cysteamine, imatinib, or pravastatin (29).

In conclusion, our study underlines the contribution of the genetic diversity of MTBC strains to key aspects of TB pathogenesis in relation to defined host conditions. While acknowledging that *in vivo* infection dynamics evolve over time, our *in vitro* findings reinforce the role of macrophage polarization in shaping intracellular stress conditions encountered by MTB and remain an underestimated component of *in vitro/ex vivo* infection models. Alveolar macrophages (resembled by M2 polarization in this study based on previous evidence) (53, 54) are more permissive to infection, and MTB is subjected to less stress and fitness costs. On the other hand, in interstitial recruited macrophages (mirrored by M1 polarization in our study), MTB is challenged with less permissive conditions (55, 56). These results align with previous studies conducted in human models. We recognize that murine studies have recently shown a shift in MTB cellular reservoirs during chronic infection stages, with monocyte-derived macrophages becoming dominant (32, 57).

Whereas previous studies showed a slower mycobacterial replication rate in interstitial recruited macrophages compared to alveolar macrophages, our data provide evidence that this feature is further influenced by the mycobacterial lineage, which affects both the cell entry rate and intra-macrophage replication. Collectively, our data support the view that reliance solely on reference laboratory strains or static macrophage models may not fully recapitulate the dynamic complexity of TB pathogenesis.

The use of a single-cell analytical approach highlighted the heterogeneity of the macrophage response to *M. tuberculosis* infection. Although linear quantile mixed models (LQMM) showed no significant differences in median acidification values between M1 and M2, logistic mixed-effects models identified three significant differences: (i) the percentage of cells showing acidification, (ii) the colocalization index, and (iii) the number of mycobacteria per cell. These results indicate that, while the average acidification level is similar between M1 and M2, the responses of individual cells differ in terms of responder proportion, timing, and infection outcome. Group-specific analyses further revealed that these differences are primarily driven by the INT and GEN groups. These findings underscore the heterogeneity of macrophage responses and highlight the importance of considering both host cell type and infection status in evaluating MTB-macrophage interactions, further emphasizing the value of single-cell approaches in detecting subtle but biologically meaningful heterogeneity. While a general increase in phagosomal acidification may be observed, our data suggest that this increment is mainly due to a relatively small proportion of cells rather than being a population-average response and is aligned with previous similar findings (32, 58). Furthermore, the differences in acidification time could be due to fluctuations in the levels of MTB damaging (virulence) factors, as previously hypothesized (59). This highlights the need for single-cell methods capable of discriminating signaling responses driven by infection.

In recent years, host-directed therapy targeting the pathogen-induced immune modulation has emerged as a promising alternative (or at least adjunct) therapy, with several benefits over antibiotic treatment alone, including reduced emergence of drug resistance and increased host compatibility (60). Our findings might have implications for host-directed therapies, particularly those targeting phagolysosomal maturation (61). Therefore, it is critical to consider MTB and macrophage heterogeneity in exploring host-directed therapeutics. In addition, our findings might influence the effects of standard anti-tubercular drugs on phagosome maturation and autophagy (29, 30). Future studies are needed to identify the individual factors responsible for lineage-specific behaviors.

## Data Availability

The raw data supporting the findings of this study have been deposited in Zenodo under DOI: 10.5281/zenodo.19594616.

## SUPPLEMENTAL MATERIAL

Figure S1 legend spectrum.03762-25-s0001.docx Expression profiling of specific markers for M1 (IL-1β, IL-6, CD80, TNF-α) and M2 (CD206) after polarization stimuli. Gene expression is provided as fold change compared to M0 THP-1-derived macrophages (endogenous control: GAPDH).

Figure S1 spectrum.03762-25-s0002.tif Evaluation of specific markers for M1 and M2.

File S1 spectrum.03762-25-s0003.pdf Supplemental methods.

File S2 spectrum.03762-25-s0004.pdf Acidification.

File S3 spectrum.03762-25-s0005.pdf Mycobacteria per cell and M1 vs M2 comparison.

File S4 spectrum.03762-25-s0006.pdf Autophagy.

File S5 spectrum.03762-25-s0007.pdf Apoptosis and necrosis.

File S6 spectrum.03762-25-s0008.pdf Intramacrophage survival.

File S7 spectrum.03762-25-s0009.pdf Representative raw confocal images corresponding to the conditions analyzed.

ASM does not own the copyrights to Supplemental Material that may be linked to, or accessed through, an article. The authors have granted ASM a non-exclusive, world-wide license to publish the Supplemental Material files. Please contact the corresponding author directly for reuse.

## References

[1] Chandra P, Grigsby SJ, Philips JA. 2022. Immune evasion and provocation by *Mycobacterium tuberculosis*. Nat Rev Microbiol 20:750–766. doi:10.1038/s41579-022-00763-4 35879556 PMC9310001

[2] Huang L, Nazarova EV, Russell DG. 2019. Bacterial fitness within the host macrophage. Microbiol Spectr 7 10.1128/microbiolspec.bai-0001-2019 PMC6459685 30848232

[3] Barbier M, Wirth T. 2016. The evolutionary history, demography, and spread of the *Mycobacterium tuberculosis* complex. Microbiol Spectr 4. doi:10.1128/microbiolspec.TBTB2-0008-2016 27726798

[4] Romagnoli A, Petruccioli E, Palucci I, Camassa S, Carata E, Petrone L, Mariano S, Sali M, Dini L, Girardi E, Delogu G, Goletti D, Fimia GM. 2018. Clinical isolates of the modern *Mycobacterium tuberculosis* lineage 4 evade host defense in human macrophages through eluding IL-1β-induced autophagy. Cell Death Dis 9:624. doi:10.1038/s41419-018-0640-8 29795378 PMC5967325

[5] Gagneux S. 2018. Ecology and evolution of *Mycobacterium tuberculosi*s. Nat Rev Microbiol 16:202–213. doi:10.1038/nrmicro.2018.8 29456241

[6] Coscolla M, Gagneux S, Menardo F, Loiseau C, Ruiz-Rodriguez P, Borrell S, Otchere ID, Asante-Poku A, Asare P, Sánchez-Busó L, et al.. 2021. Phylogenomics of *Mycobacterium africanum* reveals a new lineage and a complex evolutionary history. Microb Genom 7. doi:10.1099/mgen.0.000477 PMC8208692 33555243

[7] Guyeux C, Senelle G, Le Meur A, Supply P, Gaudin C, Phelan JE, Clark TG, Rigouts L, de Jong B, Sola C, Refrégier G. 2024. Newly identified *Mycobacterium africanum* lineage 10, Central Africa. Emerg Infect Dis 30:560–563. doi:10.3201/eid3003.231466 38407162 PMC10902520

[8] Stucki D, Brites D, Jeljeli L, Coscolla M, Liu Q, Trauner A, Fenner L, Rutaihwa L, Borrell S, Luo T, et al.. 2016. *Mycobacterium tuberculosis* lineage 4 comprises globally distributed and geographically restricted sublineages. Nat Genet 48:1535–1543. doi:10.1038/ng.3704 27798628 PMC5238942

[9] Bottai D, Frigui W, Sayes F, Di Luca M, Spadoni D, Pawlik A, Zoppo M, Orgeur M, Khanna V, Hardy D, Mangenot S, Barbe V, Medigue C, Ma L, Bouchier C, Tavanti A, Larrouy-Maumus G, Brosch R. 2020. TbD1 deletion as a driver of the evolutionary success of modern epidemic *Mycobacterium tuberculosis* lineages. Nat Commun 11:684. doi:10.1038/s41467-020-14508-5 32019932 PMC7000671

[10] Casadevall A. 2017. The pathogenic potential of a microbe. mSphere 2:mSphere doi:10.1128/mSphere.00015-17 PMC5322344 28251180

[11] Möller M, Kinnear CJ, Orlova M, Kroon EE, van Helden PD, Schurr E, Hoal EG. 2018. Genetic Resistance to *Mycobacterium tuberculosis* infection and disease. Front Immunol 9:2219. doi:10.3389/fimmu.2018.02219 30319657 PMC6170664

[12] Rosain J, Kong XF, Martinez-Barricarte R, Oleaga-Quintas C, Ramirez-Alejo N, Markle J, Okada S, Boisson-Dupuis S, Casanova JL, Bustamante J. 2019. Mendelian susceptibility to mycobacterial disease: 2014-2018 update. Immunol Cell Biol 97:360–367. doi:10.1111/imcb.12210 30264912 PMC6438774

[13] Khan A, Singh VK, Hunter RL, Jagannath C. 2019. Macrophage heterogeneity and plasticity in tuberculosis. J Leukoc Biol 106:275–282. doi:10.1002/JLB.MR0318-095RR 30938876

[14] Donald PR, Diacon AH, Lange C, Demers A-M, von Groote-Bidlingmaier F, Nardell E. 2018. Droplets, dust and guinea pigs: an historical review of tuberculosis transmission research, 1878-1940. Int J Tuberc Lung Dis 22:972–982. doi:10.5588/ijtld.18.0173 30092861

[15] Capuano SV, Croix DA, Pawar S, Zinovik A, Myers A, Lin PL, Bissel S, Fuhrman C, Klein E, Flynn JL. 2003. Experimental *Mycobacterium tuberculosis* infection of cynomolgus macaques closely resembles the various manifestations of human *M. tuberculosis* infection. Infect Immun 71:5831–5844. doi:10.1128/IAI.71.10.5831-5844.2003 14500505 PMC201048

[16] Dinkele R, Gessner S, McKerry A, Leonard B, Seldon R, Koch AS, Morrow C, Gqada M, Kamariza M, Bertozzi CR, Smith B, McLoud C, Kamholz A, Bryden W, Call C, Kaplan G, Mizrahi V, Wood R, Warner DF. 2021. Capture and visualization of live *Mycobacterium tuberculosis* bacilli from tuberculosis patient bioaerosols. PLoS Pathog 17:e1009262. doi:10.1371/journal.ppat.1009262 33524021 PMC7877778

[17] Homolka S, Niemann S, Russell DG, Rohde KH. 2010. Functional Genetic Diversity among *Mycobacterium tuberculosis* complex clinical isolates: delineation of conserved core and lineage-specific transcriptomes during intracellular survival. PLoS Pathog 6:e1000988. doi:10.1371/journal.ppat.1000988 20628579 PMC2900310

[18] Maciag A, Dainese E, Rodriguez GM, Milano A, Provvedi R, Pasca MR, Smith I, Palù G, Riccardi G, Manganelli R. 2007. Global analysis of the *Mycobacterium tuberculosis* Zur (FurB) regulon. J Bacteriol 189:730–740. doi:10.1128/JB.01190-06 17098899 PMC1797298

[19] Genin M, Clement F, Fattaccioli A, Raes M, Michiels C. 2015. M1 and M2 macrophages derived from THP-1 cells differentially modulate the response of cancer cells to etoposide. BMC Cancer 15:577. doi:10.1186/s12885-015-1546-9 26253167 PMC4545815

[20] Mantovani A, Sica A, Sozzani S, Allavena P, Vecchi A, Locati M. 2004. The chemokine system in diverse forms of macrophage activation and polarization. Trends Immunol 25:677–686. doi:10.1016/j.it.2004.09.015 15530839

[21] Murray PJ, Allen JE, Biswas SK, Fisher EA, Gilroy DW, Goerdt S, Gordon S, Hamilton JA, Ivashkiv LB, Lawrence T, Locati M, Mantovani A, Martinez FO, Mege J-L, Mosser DM, Natoli G, Saeij JP, Schultze JL, Shirey KA, Sica A, Suttles J, Udalova I, van Ginderachter JA, Vogel SN, Wynn TA. 2014. Macrophage activation and polarization: nomenclature and experimental guidelines. Immunity 41:14–20. doi:10.1016/j.immuni.2014.06.008 25035950 PMC4123412

[22] Schindelin J, Arganda-Carreras I, Frise E, Kaynig V, Longair M, Pietzsch T, Preibisch S, Rueden C, Saalfeld S, Schmid B, Tinevez JY, White DJ, Hartenstein V, Eliceiri K, Tomancak P, Cardona A. 2012. Fiji: an open-source platform for biological-image analysis. Nat Methods 9:676–682. doi:10.1038/nmeth.2019 22743772 PMC3855844

[23] Pinheiro JC, Bates DM. 2000. Mixed-effects models in S and S-PLUS. 1st ed. Springer New York, NY.

[24] Laird NM, Ware JH. 1982. Random-effects models for longitudinal data. Biometrics 38:963–974. 7168798

[25] Geraci M. 2014. Linear Quantile Mixed Models: the lqmm Package for Laplace Quantile Regression. J Stat Soft 57:1–29. doi:10.18637/jss.v057.i13

[26] Brooks M, Kristensen K, Benthem K, Magnusson A, Berg C, Nielsen A, Skaug H, Mächler M, Bolker B. 2017. glmmTMB balances speed and flexibility among packages for zero-inflated generalized linear mixed modeling. R J 9:378. doi:10.32614/RJ-2017-066

[27] R Core Team. 2013. R: a language and environment for statistical computing. R Foundation for Statistical Computing Vienna, Austria.

[28] Feoktistova M, Geserick P, Leverkus M. 2016. Crystal violet assay for determining viability of cultured cells. Cold Spring Harb Protoc. 10.1101/pdb.prot087379 27037069

[29] Palucci I, Maulucci G, De Maio F, Sali M, Romagnoli A, Petrone L, Fimia GM, Sanguinetti M, Goletti D, De Spirito M, Piacentini M, Delogu G. 2019. Inhibition of transglutaminase 2 as a potential host-directed therapy against *Mycobacterium tuberculosis*. Front Immunol 10:3042. doi:10.3389/fimmu.2019.03042 32038614 PMC6992558

[30] Genestet C, Bernard-Barret F, Hodille E, Ginevra C, Ader F, Goutelle S, Lina G, Dumitrescu O, Lyon TB study group. 2018. Antituberculous drugs modulate bacterial phagolysosome avoidance and autophagy in *Mycobacterium tuberculosis*-infected macrophages. Tuberculosis (Edinb) 111:67–70. doi:10.1016/j.tube.2018.05.014 30029917

[31] Buter J, Cheng T-Y, Ghanem M, Grootemaat AE, Raman S, Feng X, Plantijn AR, Ennis T, Wang J, Cotton RN, et al.. 2019. *Mycobacterium tuberculosis* releases an antacid that remodels phagosomes. Nat Chem Biol 15:889–899. doi:10.1038/s41589-019-0336-0 31427817 PMC6896213

[32] Zheng W, Chang IC, Limberis J, Budzik JM, Zha BS, Howard Z, Chen L, Ernst JD. 2024. Mycobacterium tuberculosis resides in lysosome-poor monocyte-derived lung cells during chronic infection. PLoS Pathog 20:e1012205. doi:10.1371/journal.ppat.1012205 38701094 PMC11095722

[33] Bedard M, van der Niet S, Bernard EM, Babunovic G, Cheng T-Y, Aylan B, Grootemaat AE, Raman S, Botella L, Ishikawa E, O’Sullivan MP, O’Leary S, Mayfield JA, Buter J, Minnaard AJ, Fortune SM, Murphy LO, Ory DS, Keane J, Yamasaki S, Gutierrez MG, van der Wel N, Moody DB. 2023. A terpene nucleoside from M. tuberculosis induces lysosomal lipid storage in foamy macrophages. J Clin Invest 133:e161944. doi:10.1172/JCI161944 36757797 PMC10014106

[34] Domenech P, Zou J, Averback A, Syed N, Curtis D, Donato S, Reed MB. 2017. Unique regulation of the DosR regulon in the beijing lineage of *Mycobacterium tuberculosis*. J Bacteriol 199:e00696-16. doi:10.1128/JB.00696-16 27799329 PMC5198487

[35] Banaei-Esfahani A, Trauner A, Borrell S, Gygli SM, Rustad TR, Feldmann J, Gillet LC, Schubert OT, Sherman DR, Beisel C, Gagneux S, Aebersold R, Collins BC. 2020. Network analysis identifies regulators of lineage-specific phenotypes in *Mycobacterium tuberculosis*. bioRxiv10.1038/s41467-026-70539-4 PMC13172132 41872185

[36] Reichlen MJ, Leistikow RL, Scobey MS, Born SEM, Voskuil MI. 2017. Anaerobic *Mycobacterium tuberculosis* cell death stems from intracellular acidification mitigated by the DosR regulon. J Bacteriol 199:e00320-17. doi:10.1128/JB.00320-17 28874407 PMC5686587

[37] Butler RE, Cihlarova V, Stewart GR. 2010. Effective generation of reactive oxygen species in the mycobacterial phagosome requires K+ efflux from the bacterium. Cell Microbiol 12:1186–1193. doi:10.1111/j.1462-5822.2010.01463.x 20331644

[38] Malone KM, Gordon SV. 2017. *Mycobacterium tuberculosis* complex members adapted to wild and domestic animals. *In* Gagneux S (ed), Strain variation in the Mycobacterium tuberculosis complex: its role in biology, epidemiology and control. Springer International Publishing, Cham. 10.1007/978-3-319-64371-7_7 29116633

[39] Panchal V, Jatana N, Malik A, Taneja B, Pal R, Bhatt A, Besra GS, Thukral L, Chaudhary S, Rao V. 2019. A novel mutation alters the stability of PapA2 resulting in the complete abrogation of sulfolipids in clinical mycobacterial strains. FASEB Bioadv 1:306–319. doi:10.1096/fba.2018-00039 32123834 PMC6996325

[40] Canton J. 2014. Phagosome maturation in polarized macrophages. J Leukoc Biol 96:729–738. doi:10.1189/jlb.1MR0114-021R 24868088

[41] Wojtas B, Fijalkowska B, Wlodarczyk A, Schollenberger A, Niemialtowski M, Hamasur B, Pawlowski A, Krzyzowska M. 2011. Mannosylated lipoarabinomannan balances apoptosis and inflammatory state in mycobacteria-infected and uninfected bystander macrophages. Microb Pathog 51:9–21. doi:10.1016/j.micpath.2011.03.004 21440050

[42] Gupta S, Rodriguez GM. 2018. Mycobacterial extracellular vesicles and host pathogen interactions. Pathog Dis 76:fty031. doi:10.1093/femspd/fty031 29722822 PMC5930244

[43] Singh PP, LeMaire C, Tan JC, Zeng E, Schorey JS. 2011. Exosomes released from M. tuberculosis infected cells can suppress IFN-γ mediated activation of naïve macrophages. PLoS One 6:e18564. doi:10.1371/journal.pone.0018564 21533172 PMC3077381

[44] Wiens KE, Woyczynski LP, Ledesma JR, Ross JM, Zenteno-Cuevas R, Goodridge A, Ullah I, Mathema B, Djoba Siawaya JF, Biehl MH, Ray SE, Bhattacharjee NV, Henry NJ, Reiner RC Jr, Kyu HH, Murray CJL, Hay SI. 2018. Global variation in bacterial strains that cause tuberculosis disease: a systematic review and meta-analysis. BMC Med 16:196. doi:10.1186/s12916-018-1180-x 30373589 PMC6206891

[45] Reiling N, Homolka S, Kohl TA, Steinhäuser C, Kolbe K, Schütze S, Brandenburg J. 2018. Shaping the niche in macrophages: genetic diversity of the M. tuberculosis complex and its consequences for the infected host. Int J Med Microbiol 308:118–128. doi:10.1016/j.ijmm.2017.09.009 28969988

[46] Fejer G, Wegner MD, Györy I, Cohen I, Engelhard P, Voronov E, Manke T, Ruzsics Z, Dölken L, Prazeres da Costa O, Branzk N, Huber M, Prasse A, Schneider R, Apte RN, Galanos C, Freudenberg MA. 2013. Nontransformed, GM-CSF-dependent macrophage lines are a unique model to study tissue macrophage functions. Proc Natl Acad Sci USA 110:E2191–8. doi:10.1073/pnas.1302877110 23708119 PMC3683787

[47] Pahari S, Arnett E, Simper J, Azad A, Guerrero-Arguero I, Ye C, Zhang H, Cai H, Wang Y, Lai Z, Jarvis N, Lumbreras M, Maselli DJ, Peters J, Torrelles JB, Martinez-Sobrido L, Schlesinger LS. 2023. A new tractable method for generating human alveolar macrophage-like cells. mBio:e0083423. 37288969 10.1128/mbio.00834-23 PMC10470505

[48] Madhvi A, Mishra H, Leisching GR, Mahlobo PZ, Baker B. 2019. Comparison of human monocyte derived macrophages and THP1-like macrophages as *in vitro* models for *M. tuberculosis* infection. Comp Immunol Microbiol Infect Dis 67:101355. doi:10.1016/j.cimid.2019.101355 31586851

[49] Kurynina AV, Erokhina MV, Makarevich OA, Sysoeva VY, Lepekha LN, Kuznetsov SA, Onishchenko GE. 2018. Plasticity of human THP-1 cell phagocytic activity during macrophagic differentiation. Biochemistry (Mosc) 83:200–214. doi:10.1134/S0006297918030021 29625541

[50] Abuawad A, Mbadugha C, Ghaemmaghami AM, Kim DH. 2020. Metabolic characterisation of THP-1 macrophage polarisation using LC-MS-based metabolite profiling. Metabolomics (Los Angel) 16:33. doi:10.1007/s11306-020-01656-4 PMC7049298 32114632

[51] Li P, Hao Z, Wu J, Ma C, Xu Y, Li J, Lan R, Zhu B, Ren P, Fan D, Sun S. 2021. Comparative proteomic analysis of polarized human THP-1 and mouse RAW264.7 macrophages. Front Immunol 12:700009. doi:10.3389/fimmu.2021.700009 34267761 PMC8276023

[52] Shah PT, Tufail M, Wu C, Xing L. 2022. THP-1 cell line model for tuberculosis: A platform for in vitro macrophage manipulation. Tuberculosis (Edinb) 136:102243. doi:10.1016/j.tube.2022.102243 35963145

[53] Lim JJ, Grinstein S, Roth Z. 2017. Diversity and versatility of phagocytosis: roles in innate immunity, tissue remodeling, and homeostasis. Front Cell Infect Microbiol 7:191. doi:10.3389/fcimb.2017.00191 28589095 PMC5440456

[54] Rothchild AC, Olson GS, Nemeth J, Amon LM, Mai D, Gold ES, Diercks AH, Aderem A. 2019. Alveolar macrophages generate a noncanonical NRF2-driven transcriptional response. Sci Immunol 4 10.1126/sciimmunol.aaw6693 PMC6910245 31350281

[55] Pisu D, Huang L, Narang V, Theriault M, Lê-Bury G, Lee B, Lakudzala AE, Mzinza DT, Mhango DV, Mitini-Nkhoma SC, Jambo KC, Singhal A, Mwandumba HC, Russell DG. 2021. Single cell analysis of M. tuberculosis phenotype and macrophage lineages in the infected lung. J Exp Med 218:e20210615. doi:10.1084/jem.20210615 34292313 PMC8302446

[56] Huang L, Nazarova EV, Tan S, Liu Y, Russell DG. 2018. Growth of *Mycobacterium tuberculosis in vivo* segregates with host macrophage metabolism and ontogeny. J Exp Med 215:1135–1152. doi:10.1084/jem.20172020 29500179 PMC5881470

[57] Zheng W, Borja M, Dorman LC, Liu J, Zhou A, Seng A, Arjyal R, Sunshine S, Nalyvayko A, Pisco AO, Rosenberg OS, Neff N, Zha BS. 2025. Single-cell analysis reveals. Sci Adv 11:eadq8158. doi:10.1126/sciadv.adq8158 39813329 PMC11734715

[58] Solomon SL, Bryson BD. 2023. Single-cell analysis reveals a weak macrophage subpopulation response to *Mycobacterium tuberculosis* infection. Cell Rep 42:113418. doi:10.1016/j.celrep.2023.113418 37963018 PMC10842899

[59] Bussi C, Gutierrez MG. 2019. *Mycobacterium tuberculosis* infection of host cells in space and time. FEMS Microbiol Rev 43:341–361. doi:10.1093/femsre/fuz006 30916769 PMC6606852

[60] Kilinç G, Saris A, Ottenhoff THM, Haks MC. 2021. Host-directed therapy to combat mycobacterial infections. Immunol Rev 301:62–83. doi:10.1111/imr.12951 33565103 PMC8248113

[61] Taya T, Teruyama F, Gojo S. 2023. Host-directed therapy for bacterial infections -Modulation of the phagolysosome pathway. Front Immunol 14:1227467. doi:10.3389/fimmu.2023.1227467 37841276 PMC10570837

