# Supplementary material for: Mycobacterium tuberculosis diversity and macrophage heterogeneity dictate phagosomal acidification
Source: Microbiol Spectr. 2026 Jul 20;14(9):e03762-25. doi: 10.1128/spectrum.03762-25 (PMC13532232; doi:10.1128/spectrum.03762-25)
Supplement: Supplementary file 1 [file spectrum.03762-25-s0003.pdf]

SUPPLEMENTARY 1

MATERIALS AND METHODS

Images and data analysis

At least 5 infected (e.g. internalized mycobacteria) plus 5 uninfected cells (e.g. exposed to mycobacteria in the same well of infected cells, but no uptake observed) (unless not available) were considered for each acquisition. Uninfected controls (CTRL) are considered macrophages not exposed to mycobacteria (in a different well). Only morphologically intact, Hoechst-positive cells were included in the single-cell acidification analysis; cells displaying features of late apoptosis or loss of nuclear integrity were excluded.

<u>Phagosomal acidification</u>: To assess acidification dynamics in infected macrophages, we employed LysoSensor staining combined with confocal microscopy and z-stack acquisition. The LysoSensor dye is an acidotropic probe exhibiting a dynamic pH-dependent increase in fluorescence intensity upon acidification only, with very low background in the cytosol. The use of LysoSensor is therefore more appropriate compared to LysoTracker dyes, as it allows to monitor pH changes rather than track acidic organelles. LysoSensor Green was used to quantify the abundance and intensity of acidified compartments in THP-1-derived M1 and M2 macrophages. To specifically assess phagosomal acidification, LysoSensor fluorescence was analyzed in conjunction with intracellular bacterial localization. Acidified compartments and mycobacteria were selected by thresholding the 458 nm and the 561 nm channels, respectively. Intracellular acidification was quantified by applying a size-filtered region of interest (ROI) mask to identify LysoSensor-positive structures, excluding background signal and capturing a defined range of acidic compartments — including but not limited to lysosomes and phagosomes. Briefly, LysoSensor-positive compartments were segmented using Fiji by applying an intensity threshold (Huang method) and size filtering (0.5–1.8 μm^2) to focus on medium- to large-sized acidified vesicles. This approach excludes background and smaller lysosomes (<0.5 μm^2) while capturing structures consistent with mature lysosomes, phagolysosomes, and other larger acidic compartments. For clarity, these LysoSensor-positive structures are hereafter referred to as acidified compartments,

encompassing large lysosomes as well as phagolysosomes. Acidified compartments and mycobacteria counts were also acquired for each single cell ROI.

To specifically assess phagosomal acidification, we performed colocalization analysis between LysoSensor fluorescence and internalized bacteria, allowing us to distinguish pathogen-associated acidified compartments from the overall lysosomal response. To analyze the spatial relationship between bacteria and lysosomes (colocalization), we wrote a custom pipeline in Matlab [2]. Briefly, the confocal stacks are maximal projected and the individual cells are segmented manually. Next, the cell nuclei and the bacteria are automatically segmented, and the average LysoSensor intensity around the segmented bacteria L_bac is calculated (in a region of 1 pixel in width). Such intensity is compared to the average LysoSensor intensity in the cell ⟨L⟩, to provide an inclusion index I_i=(L_bac-⟨L⟩)/⟨L⟩ (Figure 1a). Such index would be higher than zero when bacteria are located inside the LysoSensor-positive acidified compartments and lower than zero when bacteria are excluded from the acidified compartments. Random positioning of bacteria would provide I_i=0. Cells touching the edge of the image have been excluded from the analysis.

<u>Statistical analysis:</u> These procedures were employed to properly account for the dependence structure among observations, by including nested random-effect terms, thus considering in the model unobservable sources of heterogeneity among experimental units. For numeric variables, uninfected control THP-1 macrophages (M1 or M2, no exposure to mycobacteria) were used for normalization. Evaluation of the bystander effect, defined as an increase in acidification in uninfected macrophages imaged within the same field of view as infected cells (exposed to mycobacteria but with no detectable bacterial uptake), was performed by quantifying the percentage of acidified cells.. A cell is considered acidified if its phagosomal acidification value exceeds the upper control limit (mean+1.96SD) based on the THP-1 macrophage controls. In the linear mixed-effects models, to meet the assumption of normality of the residuals of the models, an appropriate transformation was eventually applied to the response variable; and, when necessary, few outliers identified by the specific model were not included in that analysis. Linear quantile mixed models on the median were applied to test differences between M1 and M2. Zero-inflated Mixed-effects Poisson models were applied to properly analyze count data with excess zeros. For testing difference

between pairs of groups, post-hoc analysis was performed applying the FDR procedure to account for multiple comparisons.

Spearman's correlation coefficient was calculated to evaluate the presence of a monotonic relationship between variables.

Macrophage survival

Briefly, cells were fixed with cold (-20 °C) methanol (Sigma Aldrich, Missouri, USA) and stained with Crystal violet. This assay relies on the loss of adherence of macrophages that undergo cell death or severe loss of fitness during infection, whereas viable cells remain attached to the culture surface and are retained during fixation and staining. After a wash step, the Crystal violet dye is solubilized with lysis buffer (0.1M sodium citrate, 50% Ethanol, pH 4.2) and measured by absorbance at 570 nm using the BioTek™ Gen5™ software (Agilent).

References

1. Maciag A, Dainese E, Rodriguez GM, et al. Global analysis of the *Mycobacterium tuberculosis* Zur (FurB) regulon. J Bacteriol **2007**; 189:730-40.

2. The MathWorks I. MATLAB version: 9.4.0.813654 (R2018a). **2018**.
