# Supplementary material for: Mycobacterium tuberculosis diversity and macrophage heterogeneity dictate phagosomal acidification
Source: Microbiol Spectr. 2026 Jul 20;14(9):e03762-25. doi: 10.1128/spectrum.03762-25 (PMC13532232; doi:10.1128/spectrum.03762-25)
Supplement: Supplementary file 2 [file spectrum.03762-25-s0004.pdf]

SUPPLEMENTARY 2

PHAGOSOME ACIDIFICATION

Number of M1 and M2 macrophages considered for each MTBC strain, at each timepoint:

6 h p.i.

6 h p.i.							
		NOT infected		Infected		*TOT*	
		N of cells	Replicates	N of cells	Replicates	Not inf	Inf
M1	CTRL	197	8	*na*	*na*	*1141*	*909*
	H37Rv	130	3	116	3		
	H37Ra	119	3	105	3		
	BCG	78	2	84	2		
	CDC1551	129	3	122	3		
	Beijing A	122	3	129	3		
	Beijing B	60	2	70	2		
	EAI A	67	2	66	2		
	EAI B	79	2	71	2		
	Afri A	93	2	80	2		
	Afri B	67	2	66	2		
M2	CTRL	190	8	*na*	*na*	*1116*	*932*
	H37Rv	111	3	113	3		
	BCG	73	2	84	2		
	H37Ra	131	3	121	3		
	CDC1551	134	3	123	3		
	Beijing A	126	3	148	3		
	Beijing B	60	2	70	2		
	EAI A	61	2	61	2		
	EAI B	81	2	73	2		
	Afri A	83	2	77	2		
	Afri B	66	2	62	2		
	TOT	*2257*		*1841*			
			**4098**				

24 h p.i.

24 h p.i.							
		NOT infected		Infected		*TOT*	
		N of cells	Replicates	N of cells	Replicates	Not inf	Inf
M1	CTRL	152	8	*na*	*na*	*813*	*806*
	H37Rv	77	3	110	3		
	H37Ra	73	3	111	3		
	BCG	51	2	76	2		
	CDC1551	75	3	91	3		
	Beijing A	81	3	102	3		
	Beijing B	48	2	67	2		
	EAI A	60	2	60	2		
	EAI B	76	2	67	2		
	Afri A	53	2	59	2		
	Afri B	67	2	63	2		
M2	CTRL	138	8	*na*	*na*	*859*	*808*
	H37Rv	87	3	105	3		
	BCG	70	2	80	2		
	H37Ra	94	3	109	3		
	CDC1551	84	3	82	3		
	Beijing A	83	3	98	3		
	Beijing B	43	2	64	2		
	EAI A	61	2	61	2		
	EAI B	80	2	80	2		
	Afri A	60	2	64	2		
	Afri B	59	2	65	2		
	TOT	*1672*		*1614*			
			**3286**				

Total

Overall							
		**NOT infected**		**Infected**		*TOT*	
		**N of cells**	**Replicates**	**N of cells**	**Replicates**	**Not inf**	**Inf**
	**CTRL**	349	8	*na*	*na*		
	**H37Rv**	207	3	226	3		
	**H37Ra**	192	3	216	3		
	**BCG**	129	2	160	2		
	**CDC1551**	204	3	213	3		
**M1**	**Beijing A**	203	3	231	3	*1954*	*1715*
	**Beijing B**	108	2	137	2		
	**EAI A**	127	2	126	2		
	**EAI B**	155	2	138	2		
	**Afri A**	146	2	139	2		
	**Afri B**	134	2	129	2		
	**CTRL**	328	8	*na*	*na*		
	**H37Rv**	198	3	218	3		
	**BCG**	143	2	164	2		
	**H37Ra**	225	3	230	3		
	**CDC1551**	218	3	205	3		
**M2**	**Beijing A**	209	3	246	3	*1975*	*1740*
	**Beijing B**	103	2	134	2		
	**EAI A**	122	2	122	2		
	**EAI B**	161	2	153	2		
	**Afri A**	143	2	141	2		
	**Afri B**	125	2	127	2		
	TOT	*3929*		*3455*			
		**7384**					

Imaging acquisition and analysis details

Acquisition:	obj. **63x, oil NA 1.40**<br>**z-stacks: 0.89 µm x 7 stacks**<br>Res. **16-bit**, Format: **1024x1024**<br>Laser 405 (for Hoechst) = **6%** gain: 898.9; offset: 0%<br>Laser 458 (for LysoSensor) = **10% (Argon power: 30%); gain:724.8**, offset: 0%<br>Laser 561 (for mCherry) = **8%; gain: 800**, offset 0%<br>well/sample: 3<br>acquistion postions/well: 2
Analysis:	6 h p.i. and 24 h p.i.; (N of phagolysosomes), phagolysosomes intensity and % of active cells, MOI, colocaluzation myc/acidic compartments
**Hoechst (Ex/Em 350/461)**	threshold (Huang, Red; no flags on Dark background and Stack histogram) = approx. 215<br>size = 40-inf μm^2, circularity = 0-1 (show: overlay mask; flag only Add to Manager)
**LysoSensor (Ex/Em 443/505)**	**threshold (Huang, Red; no flags on Dark background and Stack histogram) = 155**<br>**size = 0.5-1.8 μm^2, circularity = 0-1 (show: overlay mask; NO flag on Add to Manager)**
**mCherry (Ex/Em 587-610)**	**threshold (Huang, Red; no flags on Dark background and Stack histogram) = 215**<br>**size = 2-100 μm^2, circularity = 0-0.9 (show: overlay mask; NO flag on Add to Manager)**

M1 – 6 h p.i.

a) Acifdification of LysoSensor⁺ compartments

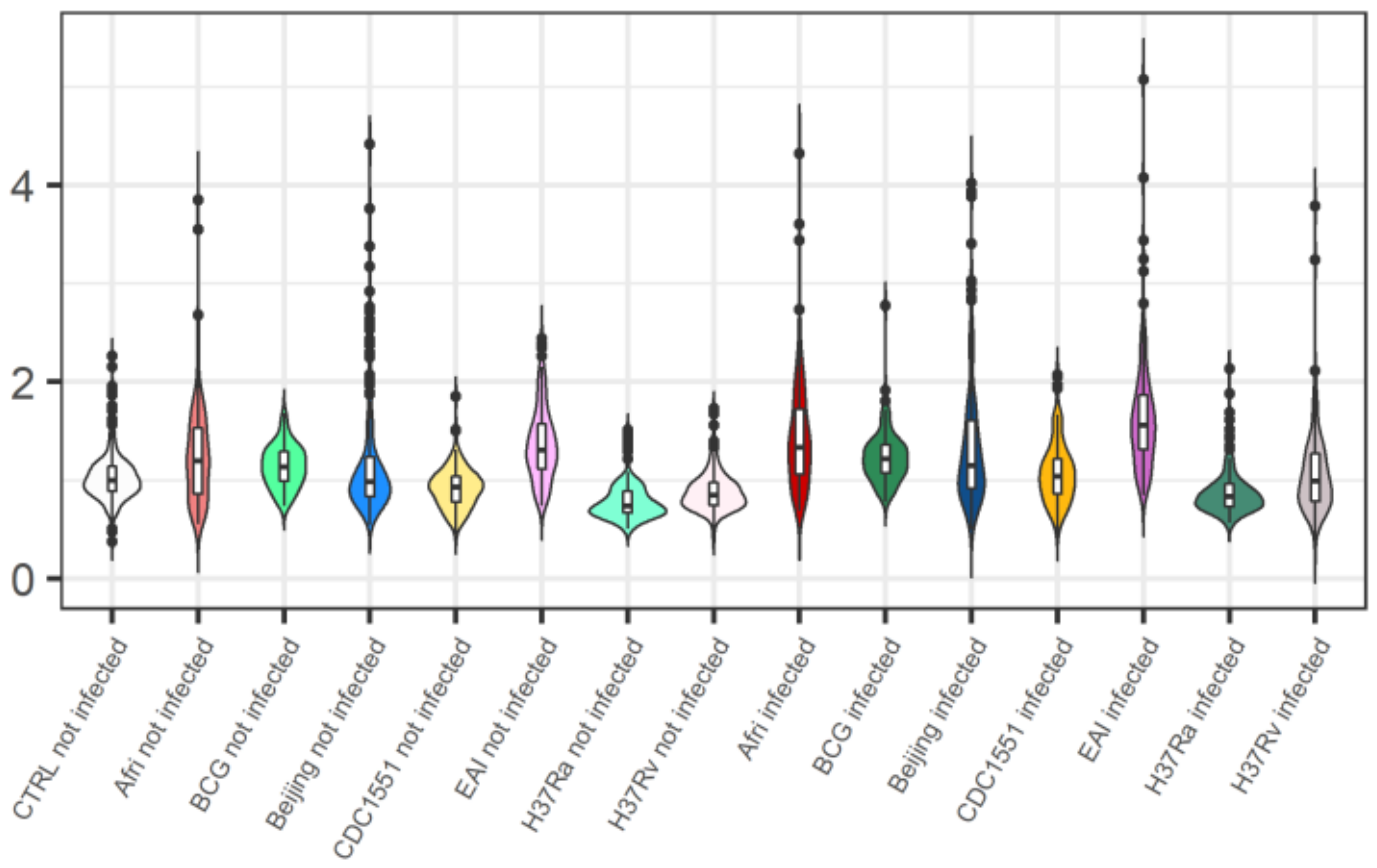

b) Percentage of cells showing acidified compartments

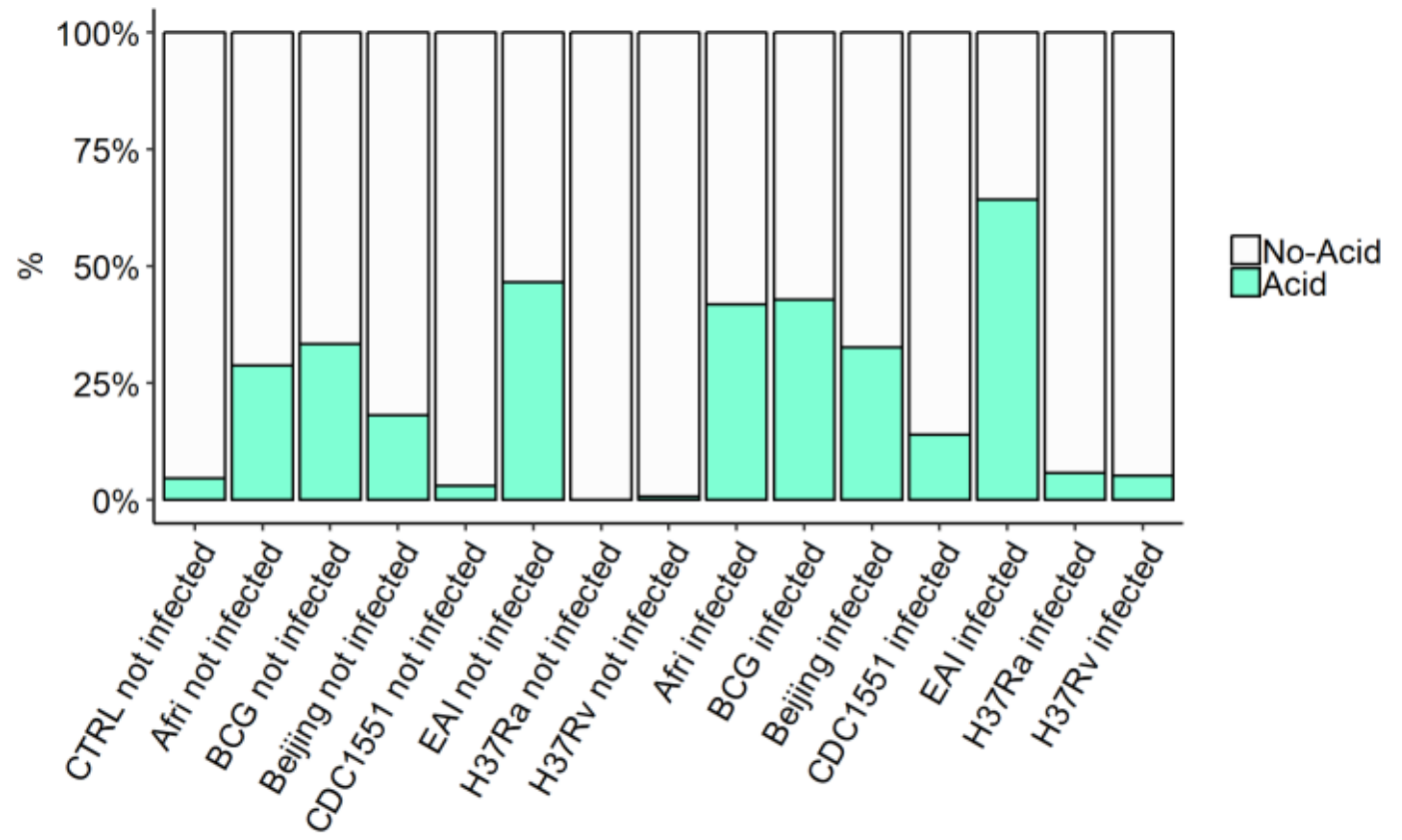

Par	Value	Std.Error	p.value
(Intercept)	0.0058	0.0668	0.9308
groupAfri not infected	0.1237	0.0695	0.0754
groupBCG not infected	0.0317	0.1052	0.7631
groupBeijing not infected	0.0952	0.0641	0.1379
groupCDC1551 not infected	-0.0414	0.0774	0.5923
groupEAI not infected	0.1613	0.075	0.0315
groupH37Ra not infected	-0.1934	0.0796	0.0152
groupH37Rv not infected	-0.0723	0.0801	0.3668
groupAfri infected	0.2767	0.0697	1e-04
groupBCG infected	0.097	0.1051	0.3562
groupBeijing infected	0.2043	0.064	0.0014
groupCDC1551 infected	0.1113	0.0776	0.1516
groupEAI infected	0.3083	0.0752	0
groupH37Ra infected	-0.112	0.08	0.1614
groupH37Rv infected	0.0413	0.0806	0.6083

Contrast	estimate	SE	p.value
none not infected-Afri not infected	-0.1295	0.079	0.2473
none not infected-BCG not infected	-0.0375	0.1119	0.7622
none not infected-Beijing not infected	-0.101	0.0743	0.3244
none not infected-CDC1551 not infected	0.0356	0.086	0.7407
none not infected-EAI not infected	-0.1671	0.084	0.1734
none not infected-H37Ra not infected	0.1876	0.0873	0.1516
none not infected-H37Rv not infected	0.0665	0.0877	0.5734
none not infected-Afri infected	-0.2825	0.0791	0.0403
none not infected-BCG infected	-0.1028	0.1118	0.5209
none not infected-Beijing infected	-0.2101	0.0742	0.0799
none not infected-CDC1551 infected	-0.1171	0.0862	0.3244
none not infected-EAI infected	-0.3141	0.0842	0.0356
none not infected-H37Ra infected	0.1062	0.0877	0.3793
none not infected-H37Rv infected	-0.0471	0.0882	0.6986
Afri not infected-BCG not infected	0.092	0.1167	0.5427
Afri not infected-Beijing not infected	0.0285	0.0724	0.7407
Afri not infected-CDC1551 not infected	0.1651	0.0858	0.1334
Afri not infected-EAI not infected	-0.0376	0.0853	0.7315
Afri not infected-H37Ra not infected	0.317	0.0932	0.0039
Afri not infected-H37Rv not infected	0.196	0.096	0.1135
BCG not infected-Beijing not infected	-0.0635	0.1143	0.6752
BCG not infected-CDC1551 not infected	0.0732	0.1243	0.6612
BCG not infected-EAI not infected	-0.1296	0.0989	0.3074
BCG not infected-H37Ra not infected	0.2251	0.1265	0.1585
BCG not infected-H37Rv not infected	0.104	0.127	0.5422
Beijing not infected-CDC1551 not infected	0.1366	0.0802	0.1734
Beijing not infected-EAI not infected	-0.0662	0.0827	0.5427
Beijing not infected-H37Ra not infected	0.2885	0.0891	0.0064
Beijing not infected-H37Rv not infected	0.1674	0.0895	0.1436
CDC1551 not infected-EAI not infected	-0.2028	0.098	0.1106
CDC1551 not infected-H37Ra not infected	0.1519	0.0968	0.2102
CDC1551 not infected-H37Rv not infected	0.0308	0.0968	0.7622
EAI not infected-H37Ra not infected	0.3547	0.1017	0.0035
EAI not infected-H37Rv not infected	0.2336	0.1025	0.0754
H37Ra not infected-H37Rv not infected	-0.1211	0.0898	0.2947
Afri infected-BCG infected	0.1798	0.1167	0.2162
Afri infected-Beijing infected	0.0725	0.0725	0.4445
Afri infected-CDC1551 infected	0.1654	0.0862	0.1334
Afri infected-EAI infected	-0.0316	0.0856	0.7478
Afri infected-H37Ra infected	0.3888	0.0936	4e-04
Afri infected-H37Rv infected	0.2354	0.0966	0.0551
BCG infected-Beijing infected	-0.1073	0.1141	0.4756
BCG infected-CDC1551 infected	-0.0143	0.1244	0.9083
BCG infected-EAI infected	-0.2113	0.099	0.0987
BCG infected-H37Ra infected	0.209	0.1267	0.1837
BCG infected-H37Rv infected	0.0557	0.1273	0.7315
Beijing infected-CDC1551 infected	0.093	0.0803	0.3621
Beijing infected-EAI infected	-0.1041	0.0827	0.3244

Par	Estimate	se	OR	pvalue
(Intercept)	-3.505	0.7631	0.03	0
groupAfri not infected	1.8572	0.7306	6.4057	0.011
groupBCG not infected	0.8416	0.9631	2.32	0.3822
groupBeijing not infected	1.1595	0.7357	3.1882	0.115
groupCDC1551 not infected	0.1986	0.9662	1.2197	0.8371
groupEAI not infected	1.7238	0.7466	5.6058	0.021
groupH37Ra not infected	-14.8469	583.2223	0	0.9797
groupH37Rv not infected	-1.2751	1.3749	0.2794	0.3537
groupAfri infected	2.998	0.7254	20.046	0
groupBCG infected	1.25	0.9578	3.4903	0.1919
groupBeijing infected	2.6757	0.7166	14.5219	2e-04
groupCDC1551 infected	1.9659	0.8611	7.1411	0.0224
groupEAI infected	2.8677	0.7499	17.5963	1e-04
groupH37Ra infected	0.8593	0.9051	2.3614	0.3424
groupH37Rv infected	0.723	1.0227	2.0606	0.4796

Contrast	estimate	SE	p.value
none not infected-Afri not infected	1.6478	0.7801	0.1456
none not infected-BCG not infected	2.6634	1.0411	0.0663
none not infected-Beijing not infected	2.3455	0.777	0.0239
none not infected-CDC1551 not infected	3.3063	0.9888	0.0104
none not infected-EAI not infected	1.7812	0.8266	0.1403
none not infected-H37Ra not infected	18.3519	583.2224	0.9814
none not infected-H37Rv not infected	4.7801	1.3959	0.0097
none not infected-Afri infected	0.5069	0.7747	0.718
none not infected-BCG infected	2.255	1.0362	0.1403
none not infected-Beijing infected	0.8293	0.7559	0.5029
none not infected-CDC1551 infected	1.5391	0.8854	0.2071
none not infected-EAI infected	0.6373	0.828	0.6468
none not infected-H37Ra infected	2.6457	0.9386	0.038
none not infected-H37Rv infected	2.782	1.0503	0.0566
Afri not infected-BCG not infected	1.0156	1.035	0.5274
Afri not infected-Beijing not infected	0.6977	0.6778	0.5029
Afri not infected-CDC1551 not infected	1.6586	0.9211	0.1917
Afri not infected-EAI not infected	0.1334	0.7717	0.9814
Afri not infected-H37Ra not infected	16.7041	583.2224	0.9814
Afri not infected-H37Rv not infected	3.1323	1.4441	0.1403
BCG not infected-Beijing not infected	-0.3179	1.0413	0.939
BCG not infected-CDC1551 not infected	0.6429	1.2377	0.8089
BCG not infected-EAI not infected	-0.8822	0.837	0.5029
BCG not infected-H37Ra not infected	15.6885	583.2229	0.9814
BCG not infected-H37Rv not infected	2.1167	1.6078	0.3948
Beijing not infected-CDC1551 not infected	0.9608	0.9201	0.5029
Beijing not infected-EAI not infected	-0.5643	0.7826	0.6742
Beijing not infected-H37Ra not infected	16.0064	583.2224	0.9814
Beijing not infected-H37Rv not infected	2.4346	1.4383	0.2112
CDC1551 not infected-EAI not infected	-1.5252	1.043	0.3232
CDC1551 not infected-H37Ra not infected	15.0455	583.2227	0.9814
CDC1551 not infected-H37Rv not infected	1.4737	1.5552	0.5275
EAI not infected-H37Ra not infected	16.5707	583.2225	0.9814
EAI not infected-H37Rv not infected	2.9989	1.4773	0.157
H37Ra not infected-H37Rv not infected	-13.5718	583.2232	0.9814
Afri infected-BCG infected	1.748	1.0262	0.2112
Afri infected-Beijing infected	0.3224	0.6516	0.8147
Afri infected-CDC1551 infected	1.0322	0.8074	0.4087
Afri infected-EAI infected	0.1303	0.7644	0.9814
Afri infected-H37Ra infected	2.1388	0.9561	0.1403
Afri infected-H37Rv infected	2.275	1.109	0.157
BCG infected-Beijing infected	-1.4257	1.0247	0.3566
BCG infected-CDC1551 infected	-0.7159	1.1531	0.7323
BCG infected-EAI infected	-1.6177	0.8452	0.1752
BCG infected-H37Ra infected	0.3907	1.2151	0.939
BCG infected-H37Rv infected	0.527	1.3151	0.8854
Beijing infected-CDC1551 infected	0.7098	0.7852	0.549
Beijing infected-EAI infected	-0.192	0.767	0.972

M1 – 6 h p.i.

c) Effective number of mycobacteria per cell (M/C)

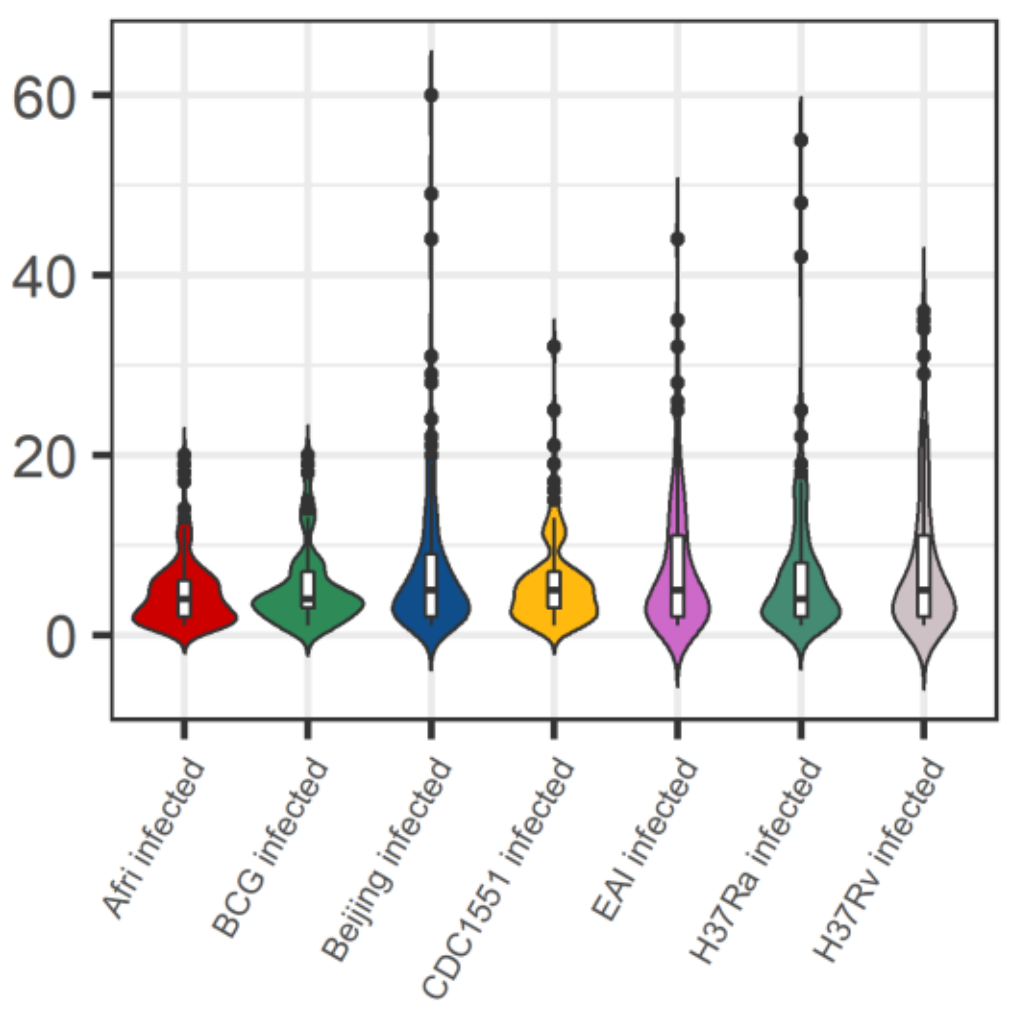

Par	Value	Std.Error	p.value
(Intercept)	1.1618	0.1532	0
groupBCG infected	0.5123	0.2551	0.0493
groupBeijing infected	0.2666	0.146	0.0729
groupCDC1551 infected	0.0771	0.1751	0.6612
groupEAI infected	0.6082	0.183	0.0015
groupH37Ra infected	0.3031	0.1976	0.1306
groupH37Rv infected	0.2976	0.2059	0.1539

Contrast	estimate	SE	p.value
Afri infected - BCG infected	-0.5123	0.2551	0.3061
Afri infected - Beijing infected	-0.2666	0.146	0.3061
Afri infected - CDC1551 infected	-0.0771	0.1751	0.7713
Afri infected - EAI infected	-0.6082	0.183	0.0324
Afri infected - H37Ra infected	-0.3031	0.1976	0.3867
Afri infected - H37Rv infected	-0.2976	0.2059	0.3867
BCG infected - Beijing infected	0.2457	0.2511	0.498
BCG infected - CDC1551 infected	0.4352	0.2743	0.3867
BCG infected - EAI infected	-0.0959	0.2033	0.7713
BCG infected - H37Ra infected	0.2092	0.2835	0.6083
BCG infected - H37Rv infected	0.2148	0.2855	0.6083
Beijing infected - CDC1551 infected	0.1895	0.1614	0.4502
Beijing infected - EAI infected	-0.3416	0.1787	0.3061
Beijing infected - H37Ra infected	-0.0364	0.1881	0.9146
Beijing infected - H37Rv infected	-0.0309	0.1897	0.9146
CDC1551 infected - EAI infected	-0.5311	0.2133	0.1645
CDC1551 infected - H37Ra infected	-0.226	0.2009	0.4502
CDC1551 infected - H37Rv infected	-0.2204	0.2016	0.4502
EAI infected - H37Ra infected	0.3051	0.227	0.3867
EAI infected - H37Rv infected	0.3107	0.2299	0.3867
H37Ra infected - H37Rv infected	0.0055	0.1847	0.9763

d) Colocalization Index

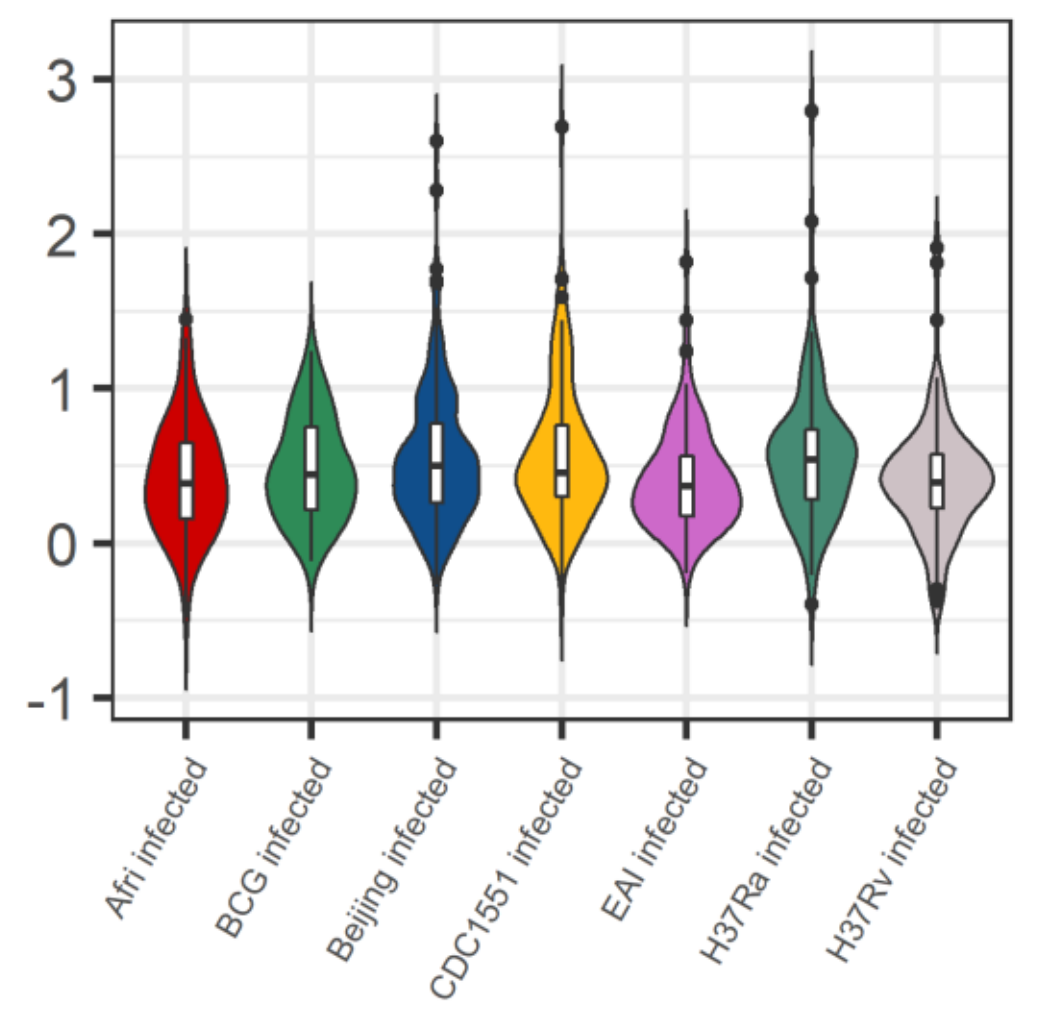

Par	Value	Std.Error	p.value
(Intercept)	-0.009	0.1222	0.9412
groupBCG infected	0.0397	0.2109	0.8513
groupBeijing infected	0.2322	0.1282	0.0752
groupCDC1551 infected	0.1699	0.1498	0.2613
groupEAI infected	-0.0855	0.1555	0.5845
groupH37Ra infected	0.2281	0.1663	0.1756
groupH37Rv infected	-0.2072	0.1721	0.2335

Contrast	estimate	SE	p.value
Afri infected - BCG infected	-0.0397	0.2109	0.8939
Afri infected - Beijing infected	-0.2322	0.1282	0.3157
Afri infected - CDC1551 infected	-0.1699	0.1498	0.5486
Afri infected - EAI infected	0.0855	0.1555	0.722
Afri infected - H37Ra infected	-0.2281	0.1663	0.4609
Afri infected - H37Rv infected	0.2072	0.1721	0.5449
BCG infected - Beijing infected	-0.1925	0.2068	0.6223
BCG infected - CDC1551 infected	-0.1302	0.2242	0.722
BCG infected - EAI infected	0.1252	0.173	0.7081
BCG infected - H37Ra infected	-0.1883	0.231	0.6756
BCG infected - H37Rv infected	0.2469	0.2313	0.5539
Beijing infected - CDC1551 infected	0.0623	0.1381	0.7623
Beijing infected - EAI infected	0.3177	0.151	0.2084
Beijing infected - H37Ra infected	0.0042	0.1592	0.9791
Beijing infected - H37Rv infected	0.4395	0.1583	0.0962
CDC1551 infected - EAI infected	0.2554	0.1773	0.4609
CDC1551 infected - H37Ra infected	-0.0581	0.1698	0.8106
CDC1551 infected - H37Rv infected	0.3771	0.1691	0.2076
EAI infected - H37Ra infected	-0.3135	0.1874	0.349
EAI infected - H37Rv infected	0.1217	0.1882	0.722
H37Ra infected - H37Rv infected	0.4353	0.1614	0.0962

M1 – 24 h p.i.

a) Acifdification of LysoSensor⁺ compartments

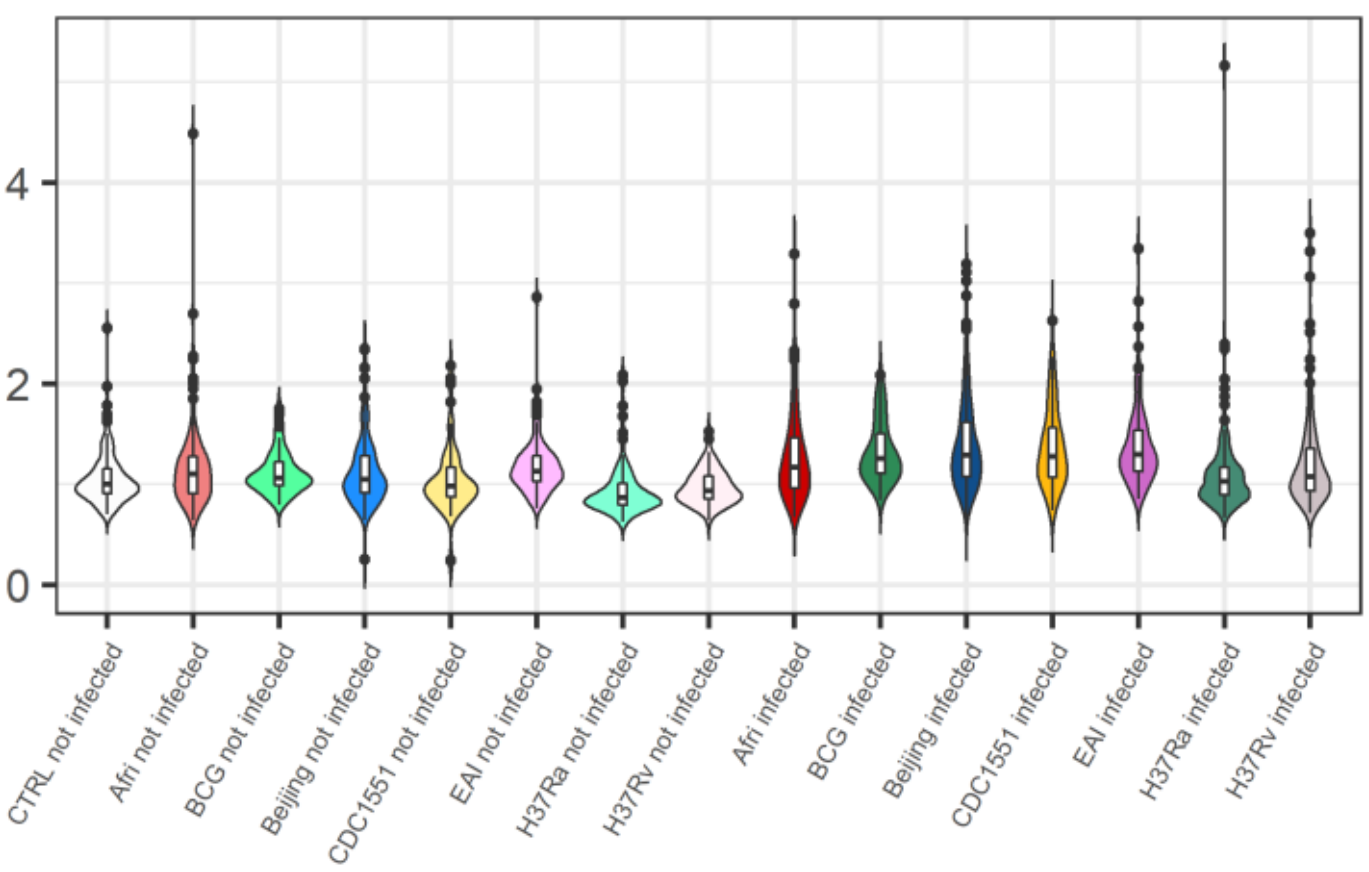

Par	Value	Std.Error	p.value
(Intercept)	0.0184	0.0353	0.6021
groupAfri not infected	0.0221	0.0389	0.5709
groupBCG not infected	0.0757	0.0572	0.1858
groupBeijing not infected	0.0485	0.0368	0.1871
groupCDC1551 not infected	0.0033	0.0444	0.9402
groupEAI not infected	0.0742	0.0406	0.0675
groupH37Ra not infected	-0.0957	0.0466	0.0403
groupH37Rv not infected	-0.0249	0.0462	0.5902
groupAfri infected	0.1127	0.0389	0.0038
groupBCG infected	0.2329	0.0548	0
groupBeijing infected	0.2026	0.0358	0
groupCDC1551 infected	0.2551	0.0435	0
groupEAI infected	0.1992	0.0408	0
groupH37Ra infected	0.0394	0.0448	0.3793
groupH37Rv infected	0.1119	0.045	0.0129

Contrast	estimate	SE	p.value
none not infected-Afri not infected	-0.0405	0.0408	0.4642
none not infected-BCG not infected	-0.0941	0.0591	0.2506
none not infected-Beijing not infected	-0.067	0.0387	0.2413
none not infected-CDC1551 not infected	-0.0218	0.0459	0.7302
none not infected-EAI not infected	-0.0927	0.0429	0.1486
none not infected-H37Ra not infected	0.0773	0.047	0.2436
none not infected-H37Rv not infected	0.0065	0.0467	0.9384
none not infected-Afri infected	-0.1312	0.0407	0.0438
none not infected-BCG infected	-0.2514	0.0568	0.0128
none not infected-Beijing infected	-0.2211	0.0378	0.0039
none not infected-CDC1551 infected	-0.2735	0.045	0.0039
none not infected-EAI infected	-0.2176	0.0431	0.0077
none not infected-H37Ra infected	-0.0578	0.0452	0.354
none not infected-H37Rv infected	-0.1303	0.0454	0.0631
Afri not infected-BCG not infected	-0.0536	0.0626	0.4942
Afri not infected-Beijing not infected	-0.0265	0.0399	0.6188
Afri not infected-CDC1551 not infected	0.0187	0.0479	0.7557
Afri not infected-EAI not infected	-0.0522	0.0453	0.3578
Afri not infected-H37Ra not infected	0.1178	0.052	0.0631
Afri not infected-H37Rv not infected	0.047	0.0529	0.4823
BCG not infected-Beijing not infected	0.0271	0.0618	0.7302
BCG not infected-CDC1551 not infected	0.0723	0.0677	0.3947
BCG not infected-EAI not infected	0.0014	0.0535	0.9868
BCG not infected-H37Ra not infected	0.1714	0.0694	0.0431
BCG not infected-H37Rv not infected	0.1006	0.0693	0.2436
Beijing not infected-CDC1551 not infected	0.0452	0.0453	0.4275
Beijing not infected-EAI not infected	-0.0257	0.0445	0.6703
Beijing not infected-H37Ra not infected	0.1443	0.0502	0.0144
Beijing not infected-H37Rv not infected	0.0734	0.0501	0.2436
CDC1551 not infected-EAI not infected	-0.0709	0.0532	0.2814
CDC1551 not infected-H37Ra not infected	0.099	0.0548	0.1486
CDC1551 not infected-H37Rv not infected	0.0282	0.0544	0.6959
EAI not infected-H37Ra not infected	0.17	0.0558	0.0114
EAI not infected-H37Rv not infected	0.0991	0.0557	0.1535
H37Ra not infected-H37Rv not infected	-0.0708	0.0509	0.2588
Afri infected-BCG infected	-0.1202	0.0605	0.1186
Afri infected-Beijing infected	-0.0899	0.0392	0.0625
Afri infected-CDC1551 infected	-0.1424	0.0471	0.0115
Afri infected-EAI infected	-0.0864	0.0455	0.1375
Afri infected-H37Ra infected	0.0733	0.0504	0.2436
Afri infected-H37Rv infected	9e-04	0.0518	0.9868
BCG infected-Beijing infected	0.0303	0.059	0.6959
BCG infected-CDC1551 infected	-0.0222	0.0651	0.7835
BCG infected-EAI infected	0.0338	0.0513	0.6188
BCG infected-H37Ra infected	0.1935	0.0663	0.0132
BCG infected-H37Rv infected	0.1211	0.0665	0.1486
Beijing infected-CDC1551 infected	-0.0525	0.0438	0.3465
Beijing infected-EAI infected	0.0035	0.0438	0.9676

b) Percentage of cells showing acidified compartments

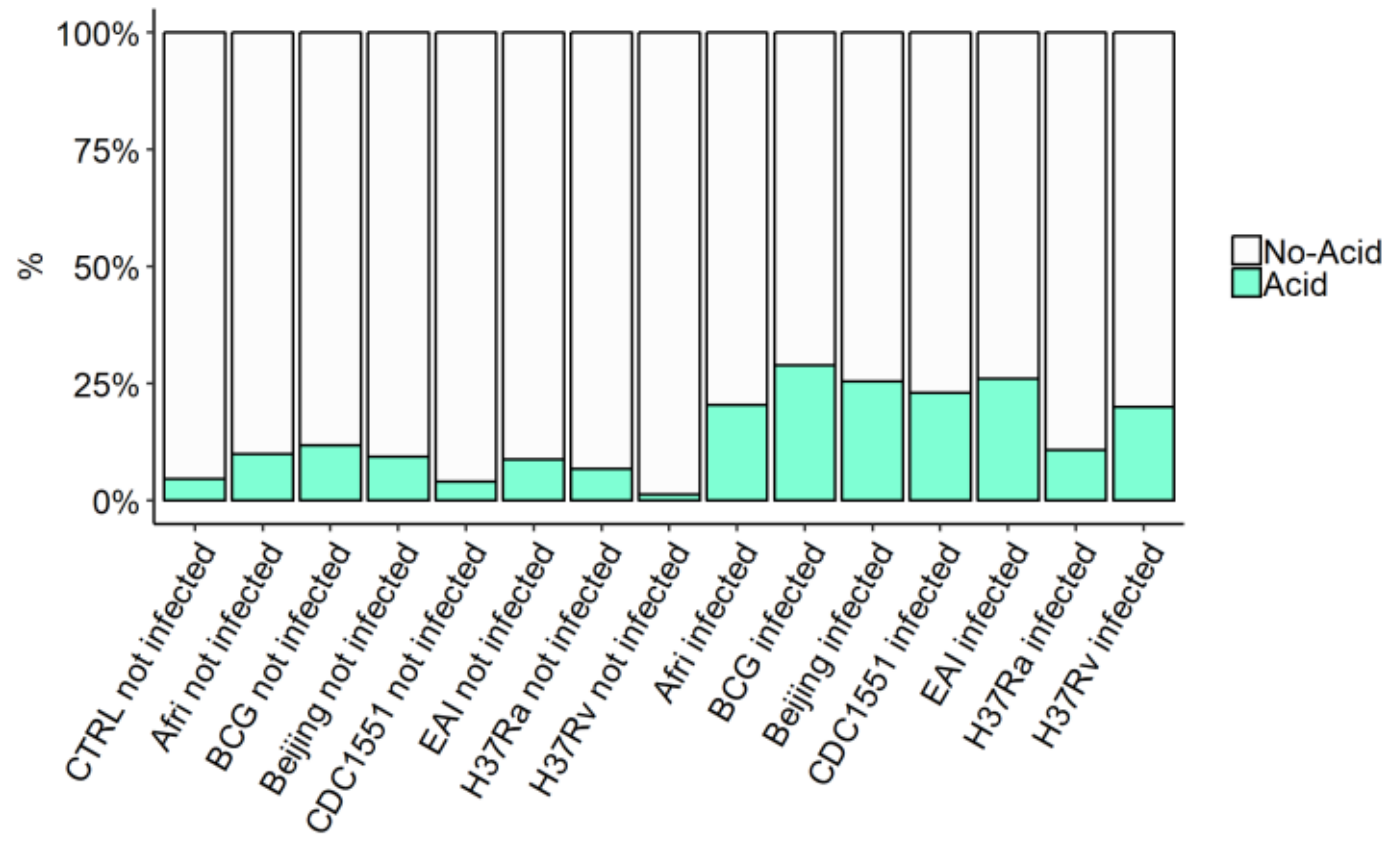

Par	Estimate	se	OR	pvalue
(Intercept)	-3.1573	0.4472	0.0425	0
groupAfri not infected	0.6976	0.5452	2.0088	0.2007
groupBCG not infected	0.8584	0.683	2.3594	0.2088
groupBeijing not infected	0.6945	0.5348	2.0028	0.1941
groupCDC1551 not infected	0.0581	0.7516	1.0598	0.9384
groupEAI not infected	0.4412	0.5449	1.5546	0.4181
groupH37Ra not infected	0.9405	0.6736	2.5612	0.1626
groupH37Rv not infected	-1.0996	1.115	0.333	0.324
groupAfri infected	1.6261	0.5024	5.0838	0.0012
groupBCG infected	1.9978	0.5825	7.3727	6e-04
groupBeijing infected	1.9321	0.4719	6.9042	0
groupCDC1551 infected	1.9848	0.5336	7.2773	2e-04
groupEAI infected	1.7757	0.496	5.9046	3e-04
groupH37Ra infected	1.422	0.5799	4.1452	0.0142
groupH37Rv infected	1.855	0.5356	6.3916	5e-04

Contrast	estimate	SE	p.value
none not infected-Afri not infected	2.4597	0.4095	0
none not infected-BCG not infected	2.2988	0.5951	6e-04
none not infected-Beijing not infected	2.4627	0.3939	0
none not infected-CDC1551 not infected	3.0991	0.6548	0
none not infected-EAI not infected	2.716	0.4226	0
none not infected-H37Ra not infected	2.2168	0.5547	5e-04
none not infected-H37Rv not infected	4.2569	1.0504	5e-04
none not infected-Afri infected	1.5312	0.349	1e-04
none not infected-BCG infected	1.1595	0.4765	0.0524
none not infected-Beijing infected	1.2251	0.3016	5e-04
none not infected-CDC1551 infected	1.1725	0.3853	0.0098
none not infected-EAI infected	1.3815	0.3567	6e-04
none not infected-H37Ra infected	1.7353	0.4357	5e-04
none not infected-H37Rv infected	1.3023	0.3821	0.003
Afri not infected-BCG not infected	-0.1609	0.6796	0.9145
Afri not infected-Beijing not infected	0.003	0.4857	0.995
Afri not infected-CDC1551 not infected	0.6394	0.7259	0.6934
Afri not infected-EAI not infected	0.2563	0.5149	0.8655
Afri not infected-H37Ra not infected	-0.2429	0.6584	0.8697
Afri not infected-H37Rv not infected	1.7972	1.1098	0.2766
BCG not infected-Beijing not infected	0.1639	0.6755	0.9145
BCG not infected-CDC1551 not infected	0.8003	0.8647	0.6934
BCG not infected-EAI not infected	0.4172	0.6115	0.7797
BCG not infected-H37Ra not infected	-0.082	0.7989	0.9483
BCG not infected-H37Rv not infected	1.958	1.1967	0.2766
Beijing not infected-CDC1551 not infected	0.6364	0.71	0.6934
Beijing not infected-EAI not infected	0.2533	0.5127	0.8655
Beijing not infected-H37Ra not infected	-0.2459	0.6453	0.8697
Beijing not infected-H37Rv not infected	1.7942	1.0982	0.2766
CDC1551 not infected-EAI not infected	-0.3831	0.7501	0.8655
CDC1551 not infected-H37Ra not infected	-0.8823	0.8231	0.6585
CDC1551 not infected-H37Rv not infected	1.1577	1.2072	0.6934
EAI not infected-H37Ra not infected	-0.4992	0.6765	0.7635
EAI not infected-H37Rv not infected	1.5408	1.1186	0.4243
H37Ra not infected-H37Rv not infected	2.0401	1.1464	0.2255
Afri infected-BCG infected	-0.3717	0.5393	0.7797
Afri infected-Beijing infected	-0.3061	0.3608	0.6934
Afri infected-CDC1551 infected	-0.3587	0.4494	0.7232
Afri infected-EAI infected	-0.1497	0.4142	0.8697
Afri infected-H37Ra infected	0.2041	0.5203	0.8697
Afri infected-H37Rv infected	-0.2289	0.478	0.8655
BCG infected-Beijing infected	0.0657	0.5166	0.9438
BCG infected-CDC1551 infected	0.013	0.5839	0.995
BCG infected-EAI infected	0.2221	0.4403	0.8655
BCG infected-H37Ra infected	0.5758	0.6279	0.6934
BCG infected-H37Rv infected	0.1428	0.59	0.9145
Beijing infected-CDC1551 infected	-0.0526	0.397	0.9438
Beijing infected-EAI infected	0.1564	0.3882	0.8697

M1 – 24 h p.i.

c) Effective number of mycobacteria per cell (M/C)

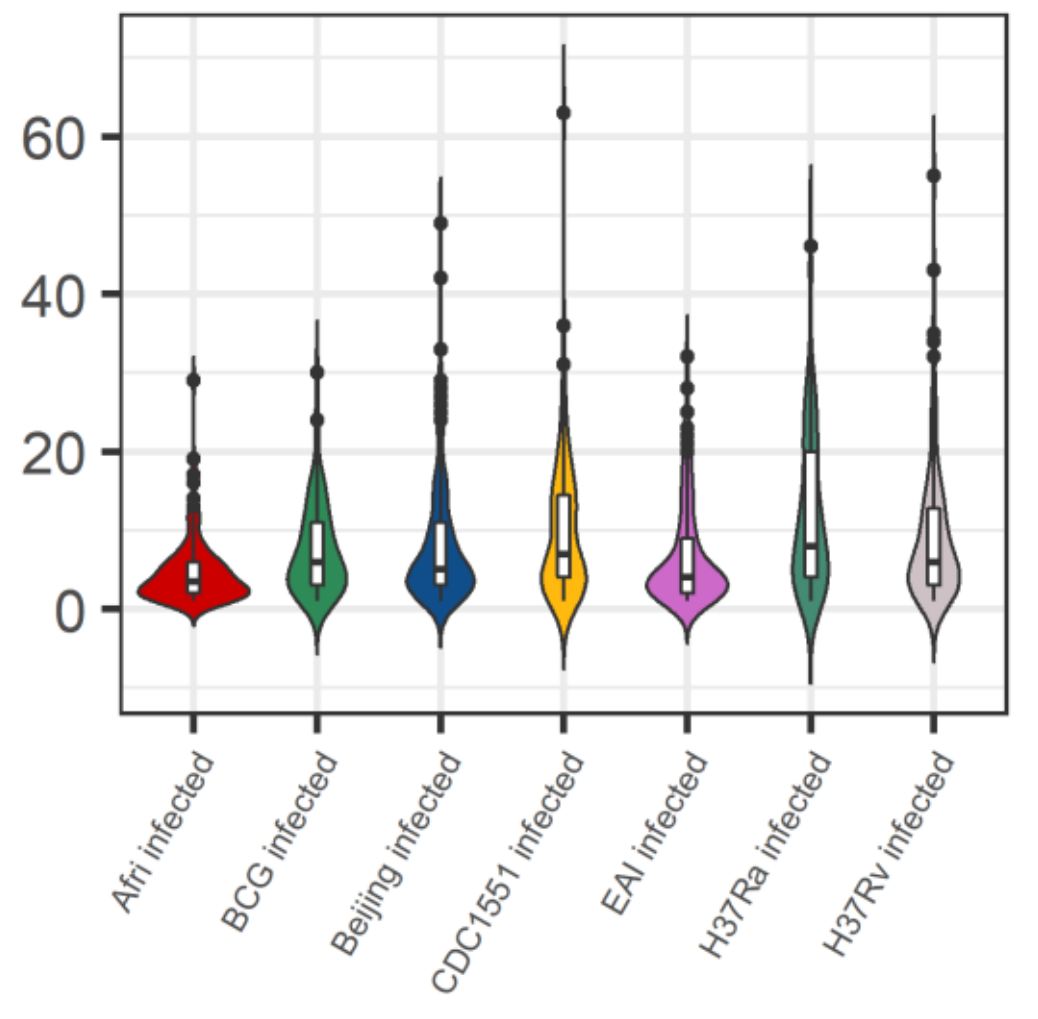

Par	Value	Std.Error	p.value
(Intercept)	1.3459	0.1565	0
groupBCG infected	0.8519	0.2039	1e-04
groupBeijing infected	0.3098	0.1109	0.0071
groupCDC1551 infected	0.367	0.1412	0.0119
groupEAI infected	0.3906	0.1448	0.0091
groupH37Ra infected	0.7005	0.162	1e-04
groupH37Rv infected	0.1793	0.1664	0.2859

Contrast	estimate	SE	p.value
Afri infected - BCG infected	-0.8519	0.2039	0.0011
Afri infected - Beijing infected	-0.3098	0.1109	0.0247
Afri infected - CDC1551 infected	-0.367	0.1412	0.0256
Afri infected - EAI infected	-0.3906	0.1448	0.0249
Afri infected - H37Ra infected	-0.7005	0.162	0.0011
Afri infected - H37Rv infected	-0.1793	0.1664	0.3753
BCG infected - Beijing infected	0.5421	0.202	0.0249
BCG infected - CDC1551 infected	0.4849	0.2259	0.0688
BCG infected - EAI infected	0.4613	0.1516	0.0185
BCG infected - H37Ra infected	0.1514	0.2369	0.6128
BCG infected - H37Rv infected	0.6726	0.2381	0.0247
Beijing infected - CDC1551 infected	-0.0572	0.1262	0.6847
Beijing infected - EAI infected	-0.0808	0.1427	0.6337
Beijing infected - H37Ra infected	-0.3907	0.151	0.0256
Beijing infected - H37Rv infected	0.1305	0.1506	0.4812
CDC1551 infected - EAI infected	-0.0236	0.1761	0.8938
CDC1551 infected - H37Ra infected	-0.3335	0.1598	0.0723
CDC1551 infected - H37Rv infected	0.1877	0.1579	0.3591
EAI infected - H37Ra infected	-0.3099	0.1911	0.1781
EAI infected - H37Rv infected	0.2113	0.1926	0.3753
H37Ra infected - H37Rv infected	0.5212	0.1322	0.0015

d) Colocalization Index

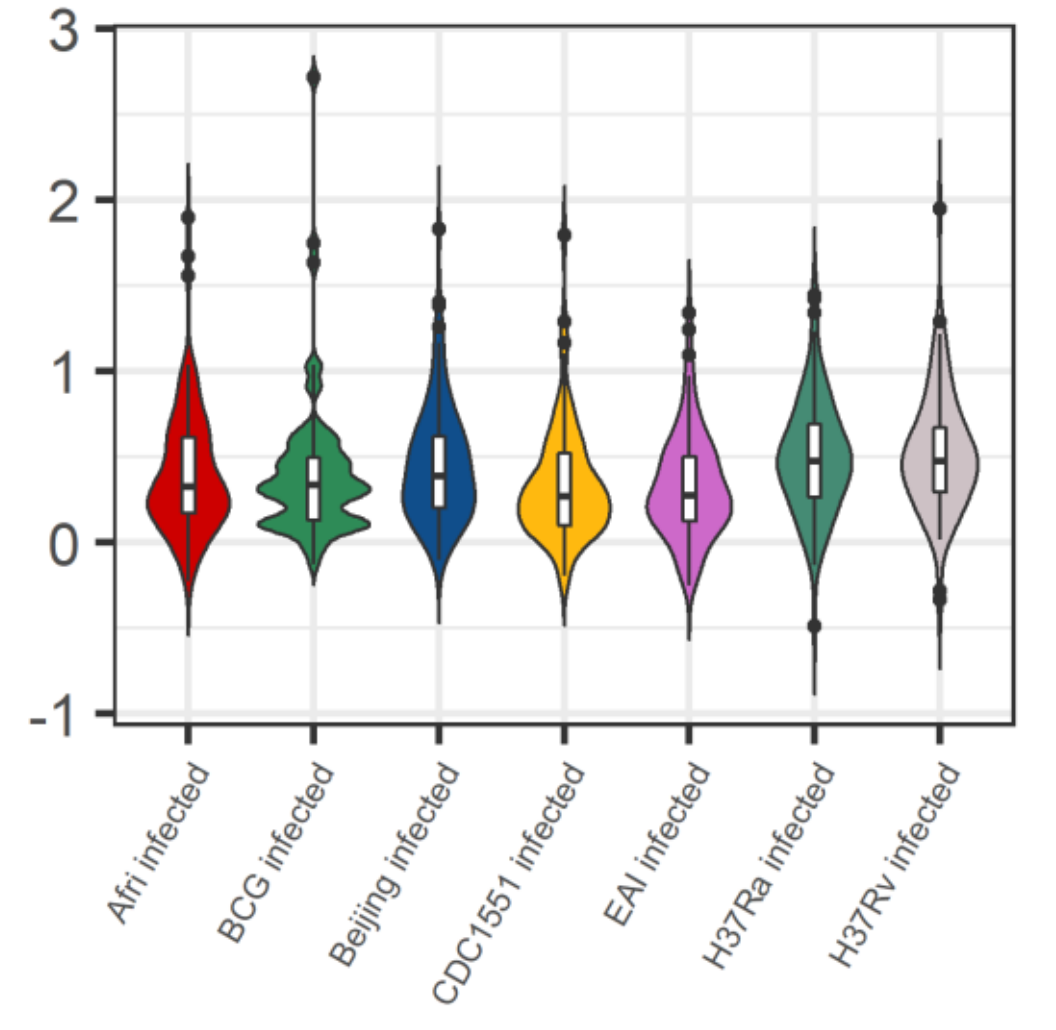

Par	Value	Std.Error	p.value
(Intercept)	-0.1611	0.1235	0.1926
groupBCG infected	-0.1594	0.1916	0.409
groupBeijing infected	0.131	0.1114	0.2443
groupCDC1551 infected	-0.1753	0.1453	0.2326
groupEAI infected	-0.2812	0.1399	0.0492
groupH37Ra infected	0.629	0.1562	2e-04
groupH37Rv infected	0.6642	0.1575	1e-04

Contrast	estimate	SE	p.value
Afri infected - BCG infected	0.1594	0.1916	0.482
Afri infected - Beijing infected	-0.131	0.1114	0.3206
Afri infected - CDC1551 infected	0.1753	0.1453	0.3206
Afri infected - EAI infected	0.2812	0.1399	0.0795
Afri infected - H37Ra infected	-0.629	0.1562	6e-04
Afri infected - H37Rv infected	-0.6642	0.1575	4e-04
BCG infected - Beijing infected	-0.2904	0.1889	0.1944
BCG infected - CDC1551 infected	0.0159	0.2133	0.9408
BCG infected - EAI infected	0.1218	0.1478	0.482
BCG infected - H37Ra infected	-0.7884	0.2173	0.0014
BCG infected - H37Rv infected	-0.8236	0.2164	0.001
Beijing infected - CDC1551 infected	0.3063	0.1345	0.0463
Beijing infected - EAI infected	0.4123	0.1368	0.0073
Beijing infected - H37Ra infected	-0.498	0.1486	0.003
Beijing infected - H37Rv infected	-0.5332	0.1463	0.0014
CDC1551 infected - EAI infected	0.1059	0.1717	0.5967
CDC1551 infected - H37Ra infected	-0.8043	0.1586	0
CDC1551 infected - H37Rv infected	-0.8395	0.1494	0
EAI infected - H37Ra infected	-0.9102	0.1782	0
EAI infected - H37Rv infected	-0.9454	0.1772	0
H37Ra infected - H37Rv infected	-0.0352	0.1294	0.8259

M2 – 6 h p.i.

a) Acifdification of LysoSensor⁺ compartments

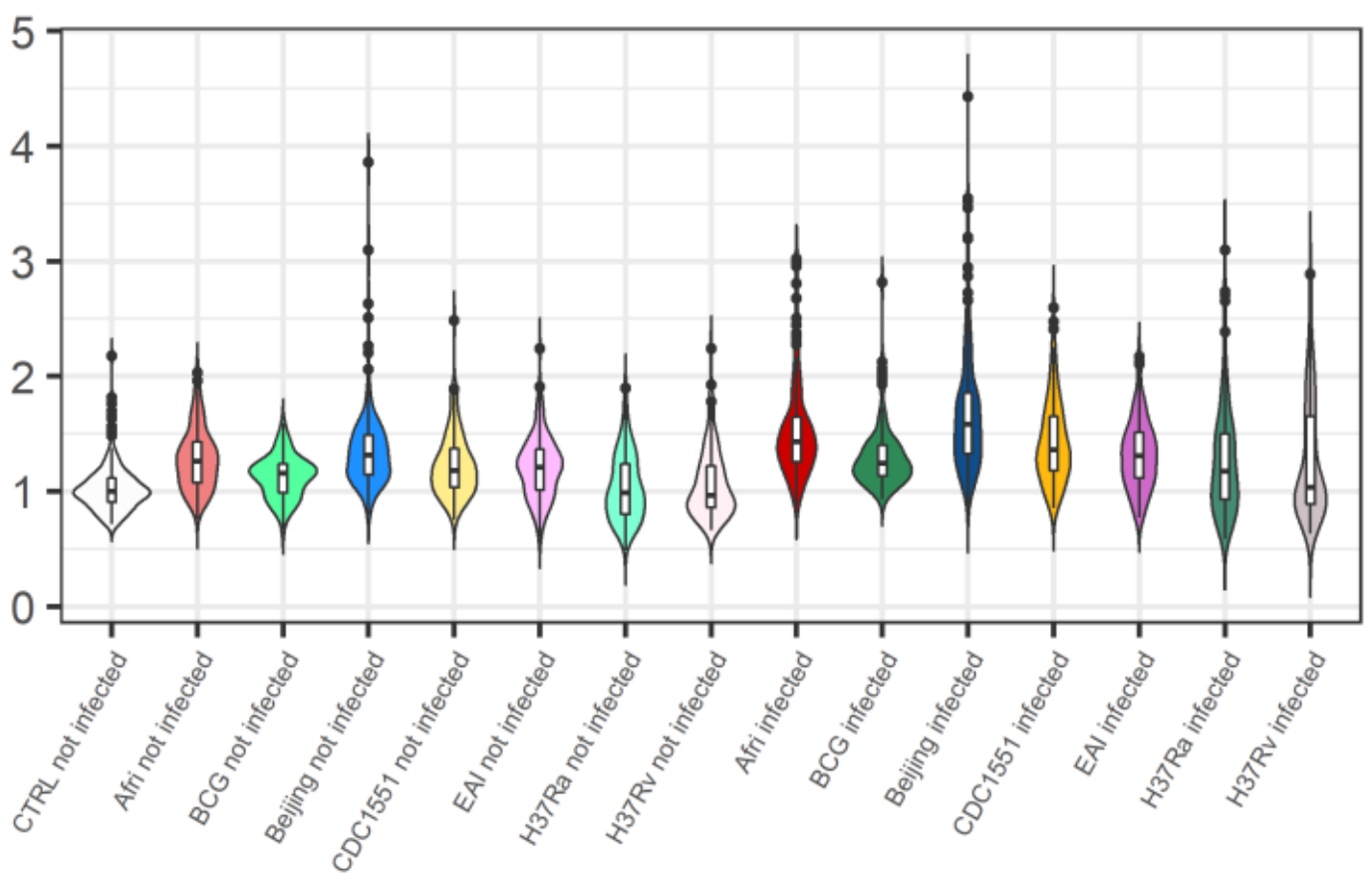

b) Percentage of cells showing acidified compartments

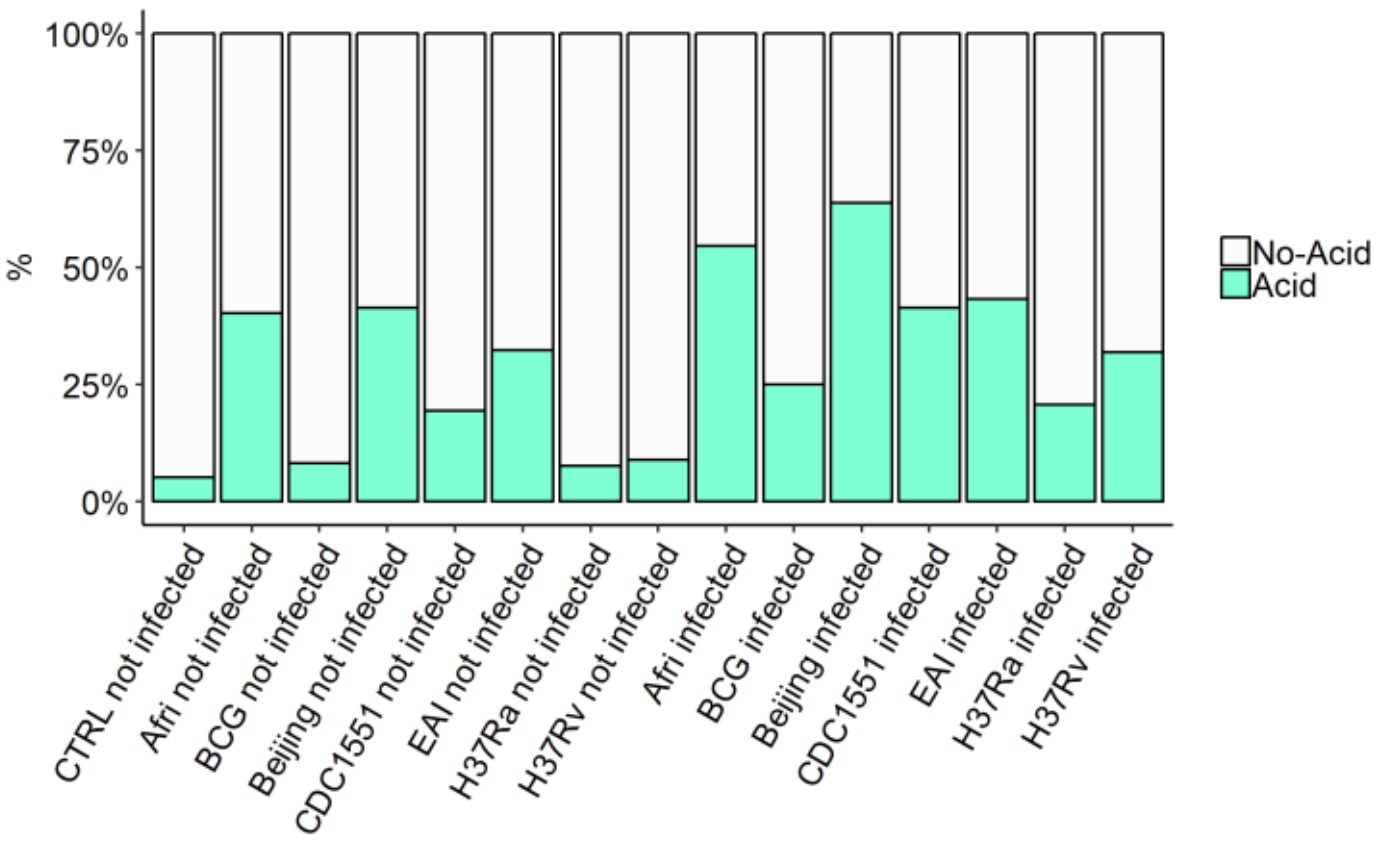

Par	Value	Std.Error	p.value
(Intercept)	-0.0102	0.0427	0.8114
groupAfri not infected	0.2373	0.0532	0
groupBCG not infected	0.0963	0.0789	0.2224
groupBeijing not infected	0.2672	0.0491	0
groupCDC1551 not infected	0.1257	0.0587	0.0325
groupEAI not infected	0.1354	0.0569	0.0174
groupH37Ra not infected	0.0167	0.0593	0.7779
groupH37Rv not infected	0.0451	0.0599	0.4519
groupAfri infected	0.3544	0.0535	0
groupBCG infected	0.1968	0.0788	0.0125
groupBeijing infected	0.4182	0.0489	0
groupCDC1551 infected	0.2551	0.059	0
groupEAI infected	0.242	0.0569	0
groupH37Ra infected	0.178	0.0597	0.0029
groupH37Rv infected	0.1709	0.06	0.0045

Contrast	estimate	SE	p.value
none not infected-Afri not infected	-0.2271	0.0533	0.0147
none not infected-BCG not infected	-0.0861	0.079	0.4273
none not infected-Beijing not infected	-0.257	0.0493	0.0055
none not infected-CDC1551 not infected	-0.1155	0.059	0.1639
none not infected-EAI not infected	-0.1252	0.0569	0.1297
none not infected-H37Ra not infected	-0.0065	0.0597	0.9175
none not infected-H37Rv not infected	-0.0349	0.0603	0.6881
none not infected-Afri infected	-0.3442	0.0536	0.0019
none not infected-BCG infected	-0.1866	0.0789	0.1048
none not infected-Beijing infected	-0.408	0.049	6e-04
none not infected-CDC1551 infected	-0.245	0.0592	0.0158
none not infected-EAI infected	-0.2319	0.057	0.0158
none not infected-H37Ra infected	-0.1679	0.0601	0.0651
none not infected-H37Rv infected	-0.1607	0.0605	0.076
Afri not infected-BCG not infected	0.141	0.0873	0.1867
Afri not infected-Beijing not infected	-0.0299	0.0555	0.6881
Afri not infected-CDC1551 not infected	0.1116	0.0653	0.1621
Afri not infected-EAI not infected	0.1019	0.0646	0.1906
Afri not infected-H37Ra not infected	0.2205	0.0696	0.0066
Afri not infected-H37Rv not infected	0.1922	0.0719	0.0232
BCG not infected-Beijing not infected	-0.1709	0.0854	0.0988
BCG not infected-CDC1551 not infected	-0.0294	0.0926	0.8301
BCG not infected-EAI not infected	-0.0391	0.0754	0.6919
BCG not infected-H37Ra not infected	0.0796	0.0936	0.5107
BCG not infected-H37Rv not infected	0.0512	0.0941	0.6881
Beijing not infected-CDC1551 not infected	0.1415	0.0611	0.0522
Beijing not infected-EAI not infected	0.1318	0.0624	0.0784
Beijing not infected-H37Ra not infected	0.2504	0.0666	0.0012
Beijing not infected-H37Rv not infected	0.2221	0.0672	0.0047
CDC1551 not infected-EAI not infected	-0.0097	0.0734	0.9175
CDC1551 not infected-H37Ra not infected	0.109	0.0726	0.2106
CDC1551 not infected-H37Rv not infected	0.0806	0.0729	0.3846
EAI not infected-H37Ra not infected	0.1187	0.0752	0.1906
EAI not infected-H37Rv not infected	0.0903	0.076	0.3606
H37Ra not infected-H37Rv not infected	-0.0283	0.0686	0.7647
Afri infected-BCG infected	0.1576	0.0874	0.1409
Afri infected-Beijing infected	-0.0638	0.0556	0.369
Afri infected-CDC1551 infected	0.0993	0.0658	0.2106
Afri infected-EAI infected	0.1124	0.0649	0.1591
Afri infected-H37Ra infected	0.1764	0.0702	0.0317
Afri infected-H37Rv infected	0.1835	0.0722	0.0305
BCG infected-Beijing infected	-0.2214	0.0851	0.0268
BCG infected-CDC1551 infected	-0.0583	0.0926	0.6667
BCG infected-EAI infected	-0.0452	0.0753	0.6773
BCG infected-H37Ra infected	0.0188	0.0937	0.8981
BCG infected-H37Rv infected	0.0259	0.094	0.8501
Beijing infected-CDC1551 infected	0.1631	0.0612	0.0232
Beijing infected-EAI infected	0.1762	0.0623	0.0158

Par	Estimate	se	OR	pvalue
(Intercept)	-3.1634	0.5967	0.0423	0
groupAfri not infected	2.4272	0.639	11.3268	1e-04
groupBCG not infected	0.4143	0.9521	1.5133	0.6634
groupBeijing not infected	2.3782	0.5865	10.7859	1e-04
groupCDC1551 not infected	1.0524	0.6893	2.8645	0.1268
groupEAI not infected	1.6846	0.6812	5.3902	0.0134
groupH37Ra not infected	1.351	0.7642	3.8612	0.0771
groupH37Rv not infected	-0.0155	0.7984	0.9846	0.9845
groupAfri infected	3.4862	0.6419	32.6619	0
groupBCG infected	1.8422	0.8825	6.3104	0.0369
groupBeijing infected	3.6119	0.5845	37.0353	0
groupCDC1551 infected	2.579	0.6714	13.1845	1e-04
groupEAI infected	2.3989	0.6793	11.0112	4e-04
groupH37Ra infected	2.6859	0.7168	14.6711	2e-04
groupH37Rv infected	2.3284	0.7232	10.2615	0.0013

Contrast	estimate	SE	p.value
none not infected-Afri not infected	0.7362	0.6118	0.3897
none not infected-BCG not infected	2.749	0.9388	0.0239
none not infected-Beijing not infected	0.7851	0.5609	0.3384
none not infected-CDC1551 not infected	2.1109	0.6688	0.0153
none not infected-EAI not infected	1.4788	0.6612	0.114
none not infected-H37Ra not infected	1.8124	0.7267	0.0663
none not infected-H37Rv not infected	3.1789	0.7727	6e-04
none not infected-Afri infected	-0.3229	0.6143	0.7402
none not infected-BCG infected	1.3212	0.8681	0.2881
none not infected-Beijing infected	-0.4485	0.5584	0.5844
none not infected-CDC1551 infected	0.5843	0.6499	0.553
none not infected-EAI infected	0.7644	0.6589	0.4078
none not infected-H37Ra infected	0.4775	0.6767	0.6177
none not infected-H37Rv infected	0.835	0.6913	0.3897
Afri not infected-BCG not infected	2.0129	0.9882	0.1544
Afri not infected-Beijing not infected	0.0489	0.5653	0.9334
Afri not infected-CDC1551 not infected	1.3748	0.6868	0.1581
Afri not infected-EAI not infected	0.7426	0.6922	0.4501
Afri not infected-H37Ra not infected	1.0762	0.7976	0.3384
Afri not infected-H37Rv not infected	2.4427	0.8509	0.0258
BCG not infected-Beijing not infected	-1.9639	0.9638	0.1544
BCG not infected-CDC1551 not infected	-0.6381	1.0514	0.6853
BCG not infected-EAI not infected	-1.2703	0.8324	0.2881
BCG not infected-H37Ra not infected	-0.9367	1.1066	0.5799
BCG not infected-H37Rv not infected	0.4298	1.1354	0.8059
Beijing not infected-CDC1551 not infected	1.3258	0.631	0.1496
Beijing not infected-EAI not infected	0.6936	0.6611	0.4519
Beijing not infected-H37Ra not infected	1.0273	0.7566	0.3384
Beijing not infected-H37Rv not infected	2.3937	0.7908	0.0195
CDC1551 not infected-EAI not infected	-0.6322	0.7953	0.5844
CDC1551 not infected-H37Ra not infected	-0.2986	0.8218	0.8059
CDC1551 not infected-H37Rv not infected	1.0679	0.8434	0.3698
EAI not infected-H37Ra not infected	0.3336	0.873	0.8059
EAI not infected-H37Rv not infected	1.7001	0.91	0.1944
H37Ra not infected-H37Rv not infected	1.3665	0.8538	0.276
Afri infected-BCG infected	1.644	0.9248	0.2067
Afri infected-Beijing infected	-0.1257	0.5665	0.8657
Afri infected-CDC1551 infected	0.9072	0.6688	0.3384
Afri infected-EAI infected	1.0873	0.6953	0.2856
Afri infected-H37Ra infected	0.8003	0.7498	0.4501
Afri infected-H37Rv infected	1.1578	0.7825	0.302
BCG infected-Beijing infected	-1.7697	0.8937	0.1581
BCG infected-CDC1551 infected	-0.7368	0.9763	0.6004
BCG infected-EAI infected	-0.5567	0.7492	0.6004
BCG infected-H37Ra infected	-0.8437	1.0131	0.5799
BCG infected-H37Rv infected	-0.4862	1.0232	0.7544
Beijing infected-CDC1551 infected	1.0328	0.6103	0.2377
Beijing infected-EAI infected	1.213	0.6569	0.1945

M2 – 6 h p.i.

c) Effective number of mycobacteria per cell (M/C)

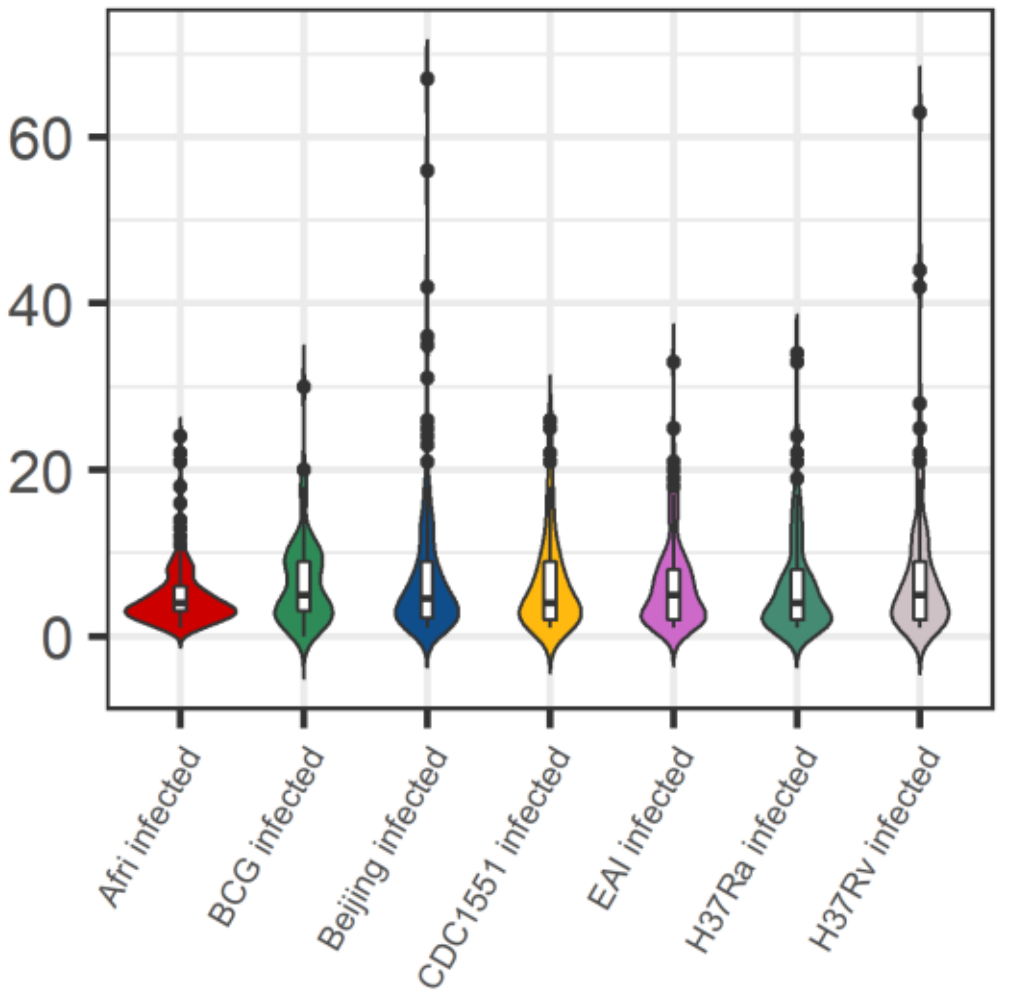

Par	Value	Std.Error	p.value
(Intercept)	0.0121	0.174	0.9446
groupBCG infected	0.229	0.2207	0.3039
groupBeijing infected	0.0098	0.1149	0.9324
groupCDC1551 infected	-0.0838	0.1396	0.5507
groupEAI infected	0.2112	0.1578	0.186
groupH37Ra infected	-0.3097	0.1632	0.0628
groupH37Rv infected	-0.2894	0.176	0.1055

Contrast	estimate	SE	p.value
Afri infected - BCG infected	-0.229	0.2207	0.4195
Afri infected - Beijing infected	-0.0098	0.1149	0.9324
Afri infected - CDC1551 infected	0.0838	0.1396	0.6425
Afri infected - EAI infected	-0.2112	0.1578	0.3211
Afri infected - H37Ra infected	0.3097	0.1632	0.1977
Afri infected - H37Rv infected	0.2894	0.176	0.2694
BCG infected - Beijing infected	0.2192	0.2183	0.4195
BCG infected - CDC1551 infected	0.3127	0.2395	0.3211
BCG infected - EAI infected	0.0178	0.1622	0.9324
BCG infected - H37Ra infected	0.5387	0.2516	0.1977
BCG infected - H37Rv infected	0.5184	0.2574	0.1977
Beijing infected - CDC1551 infected	0.0936	0.125	0.5648
Beijing infected - EAI infected	-0.2014	0.1549	0.3211
Beijing infected - H37Ra infected	0.3195	0.1521	0.1977
Beijing infected - H37Rv infected	0.2992	0.1596	0.1977
CDC1551 infected - EAI infected	-0.2949	0.1846	0.2694
CDC1551 infected - H37Ra infected	0.226	0.1612	0.3211
CDC1551 infected - H37Rv infected	0.2057	0.1685	0.3406
EAI infected - H37Ra infected	0.5209	0.2009	0.1977
EAI infected - H37Rv infected	0.5006	0.2083	0.1977
H37Ra infected - H37Rv infected	-0.0203	0.1445	0.9324

d) Colocalization Index

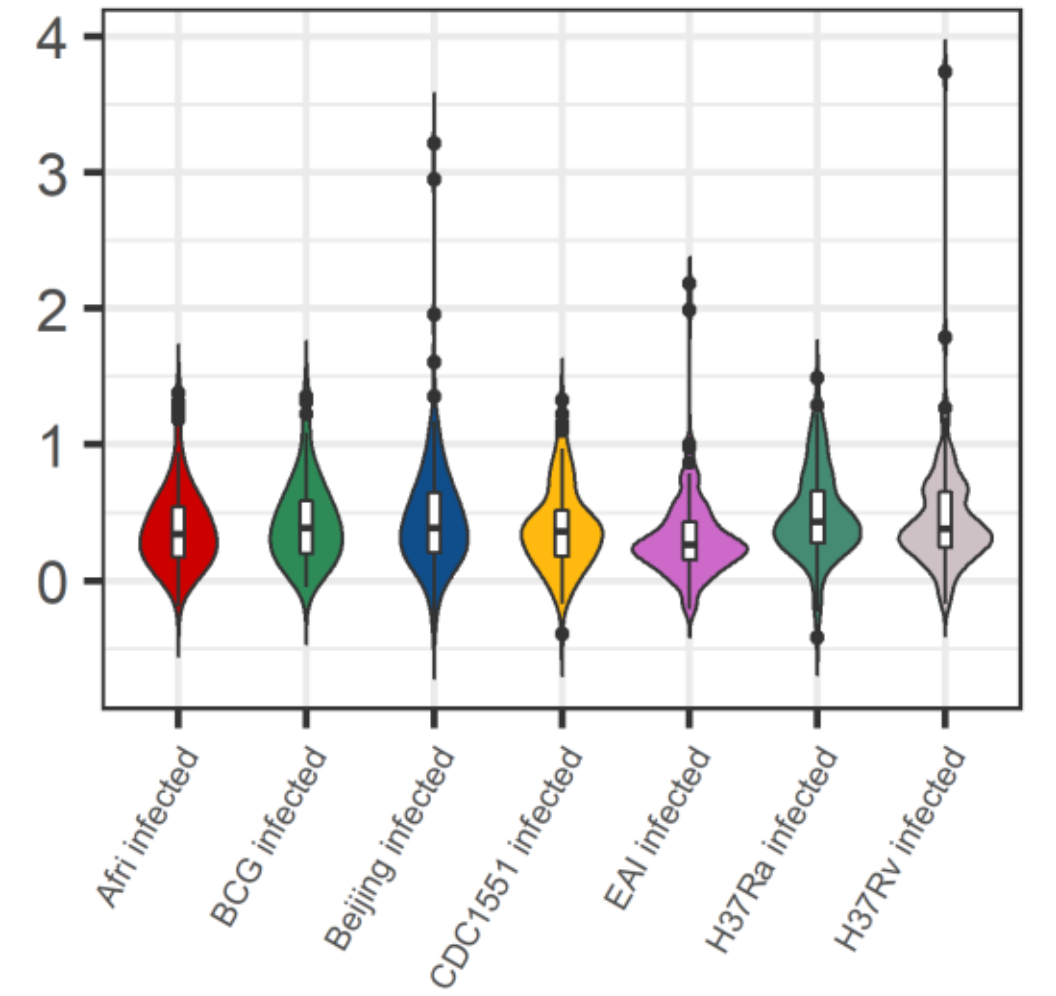

Par	Value	Std.Error	p.value
(Intercept)	-0.2367	0.0657	3e-04
groupBCG infected	0.1292	0.1071	0.2323
groupBeijing infected	0.2211	0.084	0.0108
groupCDC1551 infected	0.054	0.0961	0.5762
groupEAI infected	-0.1516	0.0943	0.1135
groupH37Ra infected	0.3735	0.0968	3e-04
groupH37Rv infected	0.1878	0.0981	0.0605

Contrast	estimate	SE	p.value
Afri infected - BCG infected	-0.1292	0.1071	0.3049
Afri infected - Beijing infected	-0.2211	0.084	0.036
Afri infected - CDC1551 infected	-0.054	0.0961	0.6318
Afri infected - EAI infected	0.1516	0.0943	0.1702
Afri infected - H37Ra infected	-0.3735	0.0968	0.002
Afri infected - H37Rv infected	-0.1878	0.0981	0.1163
BCG infected - Beijing infected	-0.0919	0.0994	0.4433
BCG infected - CDC1551 infected	0.0752	0.1098	0.5787
BCG infected - EAI infected	0.2808	0.1083	0.036
BCG infected - H37Ra infected	-0.2443	0.1104	0.0811
BCG infected - H37Rv infected	-0.0585	0.1116	0.6318
Beijing infected - CDC1551 infected	0.1671	0.0874	0.1163
Beijing infected - EAI infected	0.3727	0.0855	6e-04
Beijing infected - H37Ra infected	-0.1524	0.0882	0.1445
Beijing infected - H37Rv infected	0.0334	0.0896	0.7111
CDC1551 infected - EAI infected	0.2056	0.0974	0.0913
CDC1551 infected - H37Ra infected	-0.3195	0.0998	0.0093
CDC1551 infected - H37Rv infected	-0.1338	0.1011	0.2673
EAI infected - H37Ra infected	-0.5251	0.0981	0
EAI infected - H37Rv infected	-0.3394	0.0994	0.0062
H37Ra infected - H37Rv infected	0.1858	0.1018	0.1278

M2 – 24 h p.i.

a) Acifdification of LysoSensor+ compartments

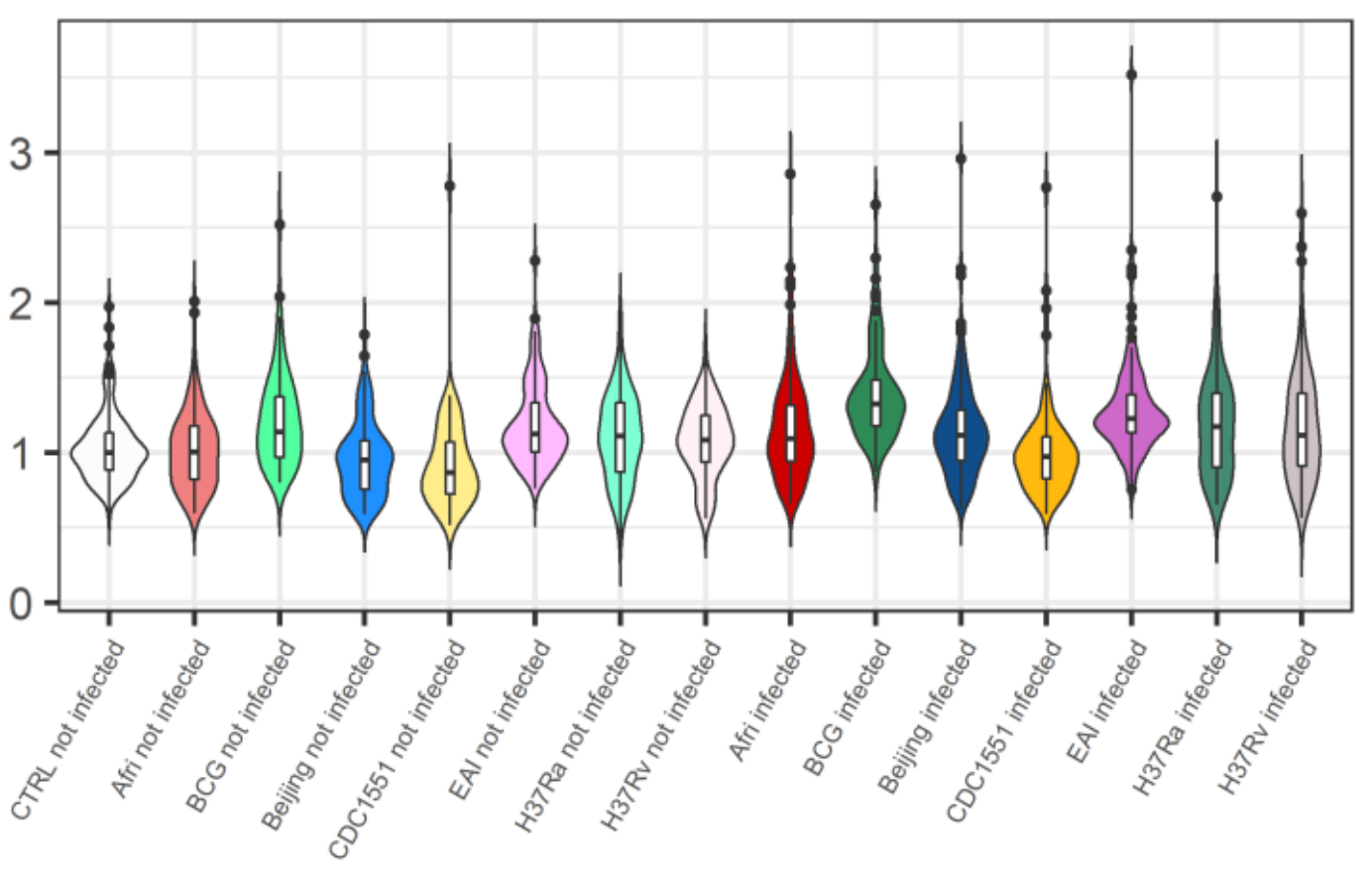

b) Percentage of cells showing acidified compartments

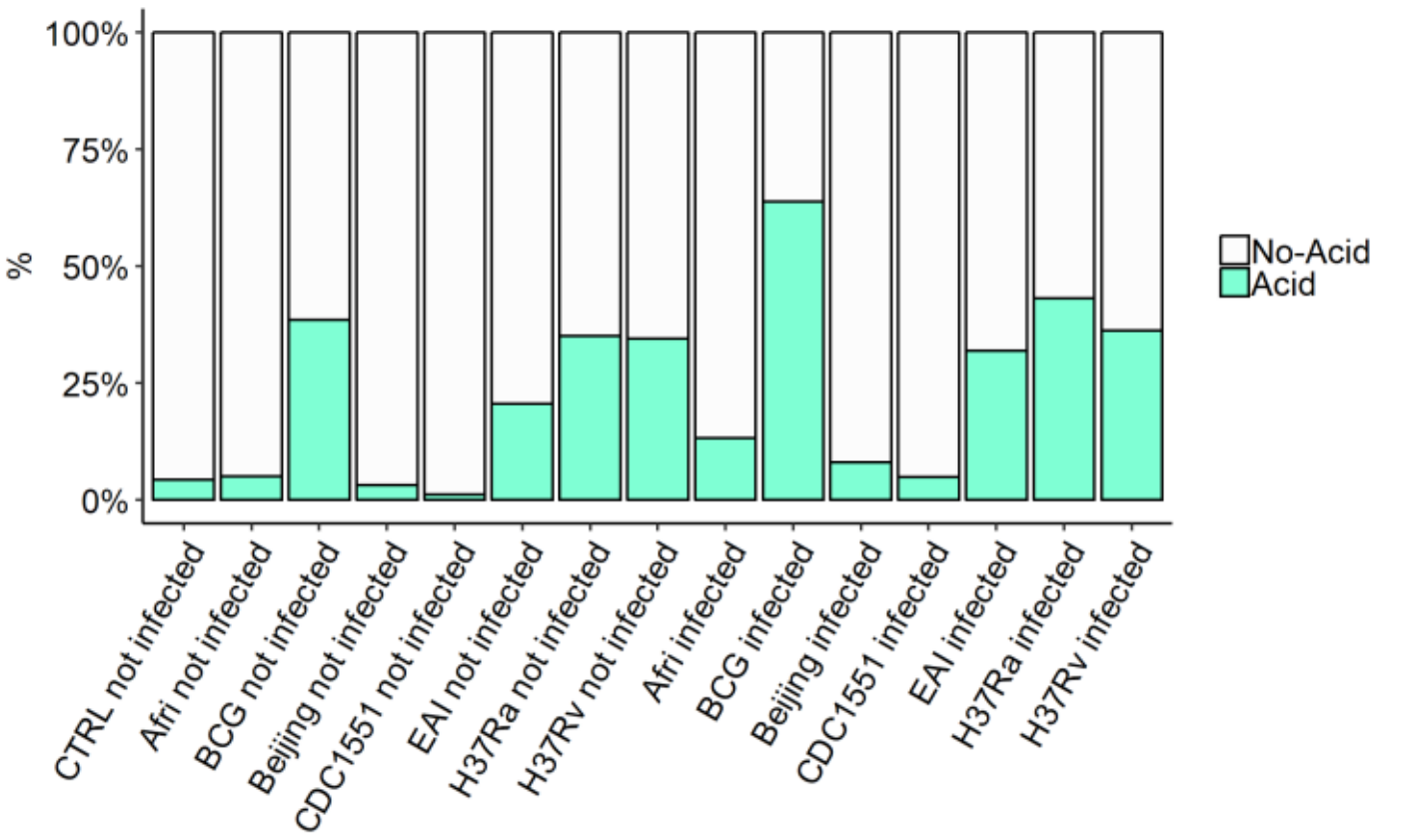

Par	Value	Std.Error	p.value
(Intercept)	0.0139	0.0526	0.7917
groupAfri not infected	-0.0115	0.0369	0.7548
groupBCG not infected	0.0972	0.054	0.0724
groupBeijing not infected	-0.0691	0.0351	0.0492
groupCDC1551 not infected	-0.0907	0.0411	0.0275
groupEAI not infected	0.0767	0.0392	0.0506
groupH37Ra not infected	-0.0554	0.044	0.2083
groupH37Rv not infected	-0.1223	0.0446	0.0061
groupAfri infected	0.1065	0.0368	0.0039
groupBCG infected	0.2481	0.0534	0
groupBeijing infected	0.0653	0.034	0.055
groupCDC1551 infected	-0.023	0.0416	0.5806
groupEAI infected	0.1561	0.0393	1e-04
groupH37Ra infected	0.0528	0.0426	0.2149
groupH37Rv infected	-0.0563	0.0435	0.195

Contrast	estimate	SE	p.value
none not infected-Afri not infected	-0.0024	0.056	0.9672
none not infected-BCG not infected	-0.1111	0.0691	0.2339
none not infected-Beijing not infected	0.0552	0.0549	0.4292
none not infected-CDC1551 not infected	0.0768	0.0588	0.3256
none not infected-EAI not infected	-0.0906	0.058	0.2376
none not infected-H37Ra not infected	0.0415	0.0602	0.5771
none not infected-H37Rv not infected	0.1084	0.0606	0.1932
none not infected-Afri infected	-0.1203	0.056	0.1202
none not infected-BCG infected	-0.262	0.0687	0.0207
none not infected-Beijing infected	-0.0792	0.0541	0.2675
none not infected-CDC1551 infected	0.0091	0.0591	0.8966
none not infected-EAI infected	-0.17	0.0581	0.0498
none not infected-H37Ra infected	-0.0667	0.0592	0.3739
none not infected-H37Rv infected	0.0424	0.0598	0.5771
Afri not infected-BCG not infected	-0.1087	0.0593	0.1202
Afri not infected-Beijing not infected	0.0576	0.0376	0.202
Afri not infected-CDC1551 not infected	0.0792	0.0441	0.1238
Afri not infected-EAI not infected	-0.0882	0.0434	0.0835
Afri not infected-H37Ra not infected	0.0438	0.0495	0.4553
Afri not infected-H37Rv not infected	0.1108	0.0514	0.0664
BCG not infected-Beijing not infected	0.1663	0.0588	0.0158
BCG not infected-CDC1551 not infected	0.1879	0.064	0.0126
BCG not infected-EAI not infected	0.0205	0.049	0.7218
BCG not infected-H37Ra not infected	0.1525	0.0666	0.0498
BCG not infected-H37Rv not infected	0.2195	0.0672	0.0054
Beijing not infected-CDC1551 not infected	0.0216	0.0418	0.6575
Beijing not infected-EAI not infected	-0.1458	0.043	0.0041
Beijing not infected-H37Ra not infected	-0.0138	0.0478	0.8113
Beijing not infected-H37Rv not infected	0.0532	0.0488	0.3547
CDC1551 not infected-EAI not infected	-0.1674	0.0507	0.0051
CDC1551 not infected-H37Ra not infected	-0.0354	0.0509	0.5771
CDC1551 not infected-H37Rv not infected	0.0316	0.0515	0.5966
EAI not infected-H37Ra not infected	0.132	0.0542	0.0395
EAI not infected-H37Rv not infected	0.199	0.0551	0.0022
H37Ra not infected-H37Rv not infected	0.067	0.0474	0.2367
Afri infected-BCG infected	-0.1417	0.0587	0.0395
Afri infected-Beijing infected	0.0411	0.0367	0.3514
Afri infected-CDC1551 infected	0.1294	0.0446	0.0131
Afri infected-EAI infected	-0.0496	0.0434	0.3468
Afri infected-H37Ra infected	0.0536	0.0484	0.3514
Afri infected-H37Rv infected	0.1628	0.0504	0.0057
BCG infected-Beijing infected	0.1828	0.0576	0.0064
BCG infected-CDC1551 infected	0.2711	0.0638	3e-04
BCG infected-EAI infected	0.092	0.0484	0.1061
BCG infected-H37Ra infected	0.1953	0.0652	0.011
BCG infected-H37Rv infected	0.3045	0.066	1e-04
Beijing infected-CDC1551 infected	0.0883	0.0414	0.0674
Beijing infected-EAI infected	-0.0908	0.0422	0.0664

Par	Estimate	se	OR	pvalue
(Intercept)	-3.484	0.6133	0.0307	0
groupAfri not infected	0.9109	0.6428	2.4866	0.1565
groupBCG not infected	1.7782	0.5663	5.9191	0.0017
groupBeijing not infected	0.5988	0.7014	1.82	0.3933
groupCDC1551 not infected	-0.2013	1.1277	0.8176	0.8583
groupEAI not infected	1.3244	0.5316	3.7599	0.0127
groupH37Ra not infected	1.4107	0.5892	4.0989	0.0166
groupH37Rv not infected	1.1886	0.5907	3.2824	0.0442
groupAfri infected	1.9846	0.5506	7.2758	3e-04
groupBCG infected	2.805	0.5607	16.5272	0
groupBeijing infected	1.4598	0.5585	4.3052	0.009
groupCDC1551 infected	1.3262	0.7207	3.7668	0.0657
groupEAI infected	1.951	0.5188	7.0354	2e-04
groupH37Ra infected	2.3266	0.5764	10.243	1e-04
groupH37Rv infected	1.7108	0.5785	5.5335	0.0031

Contrast	estimate	SE	p.value
none not infected-Afri not infected	2.5731	0.6172	0.001
none not infected-BCG not infected	1.7058	0.5856	0.0189
none not infected-Beijing not infected	2.8851	0.6739	0.001
none not infected-CDC1551 not infected	3.6853	1.1059	0.0077
none not infected-EAI not infected	2.1596	0.5412	0.0011
none not infected-H37Ra not infected	2.0732	0.5744	0.0032
none not infected-H37Rv not infected	2.2954	0.5766	0.0011
none not infected-Afri infected	1.4994	0.5203	0.0192
none not infected-BCG infected	0.6789	0.5802	0.3944
none not infected-Beijing infected	2.0241	0.5265	0.0015
none not infected-CDC1551 infected	2.1577	0.6859	0.0116
none not infected-EAI infected	1.533	0.5264	0.0189
none not infected-H37Ra infected	1.1574	0.5541	0.1178
none not infected-H37Rv infected	1.7731	0.56	0.0116
Afri not infected-BCG not infected	-0.8673	0.5955	0.2996
Afri not infected-Beijing not infected	0.3121	0.6701	0.7089
Afri not infected-CDC1551 not infected	1.1122	1.1161	0.4785
Afri not infected-EAI not infected	-0.4135	0.543	0.5859
Afri not infected-H37Ra not infected	-0.4998	0.6498	0.5859
Afri not infected-H37Rv not infected	-0.2777	0.6554	0.7174
BCG not infected-Beijing not infected	1.1794	0.6683	0.2131
BCG not infected-CDC1551 not infected	1.9795	1.1224	0.2131
BCG not infected-EAI not infected	0.4538	0.3524	0.3666
BCG not infected-H37Ra not infected	0.3675	0.6323	0.6653
BCG not infected-H37Rv not infected	0.5896	0.6358	0.5065
Beijing not infected-CDC1551 not infected	0.8001	1.1422	0.6218
Beijing not infected-EAI not infected	-0.7256	0.6229	0.3944
Beijing not infected-H37Ra not infected	-0.8119	0.6971	0.3944
Beijing not infected-H37Rv not infected	-0.5898	0.701	0.5602
CDC1551 not infected-EAI not infected	-1.5257	1.0974	0.3237
CDC1551 not infected-H37Ra not infected	-1.6121	1.1087	0.2996
CDC1551 not infected-H37Rv not infected	-1.3899	1.1093	0.3679
EAI not infected-H37Ra not infected	-0.0863	0.5905	0.898
EAI not infected-H37Rv not infected	0.1358	0.5944	0.8569
H37Ra not infected-H37Rv not infected	0.2221	0.3913	0.6653
Afri infected-BCG infected	-0.8205	0.4894	0.2459
Afri infected-Beijing infected	0.5247	0.4148	0.3679
Afri infected-CDC1551 infected	0.6583	0.6258	0.45
Afri infected-EAI infected	0.0336	0.4105	0.9348
Afri infected-H37Ra infected	-0.342	0.5317	0.6424
Afri infected-H37Rv infected	0.2737	0.5451	0.7051
BCG infected-Beijing infected	1.3452	0.5093	0.0372
BCG infected-CDC1551 infected	1.4788	0.7104	0.1178
BCG infected-EAI infected	0.8541	0.3319	0.0423
BCG infected-H37Ra infected	0.4784	0.6097	0.5859
BCG infected-H37Rv infected	1.0942	0.6158	0.2131
Beijing infected-CDC1551 infected	0.1336	0.621	0.8569
Beijing infected-EAI infected	-0.4911	0.4352	0.4082

M2 – 24 h p.i.

c) Effective number of mycobacteria per cell (M/C)

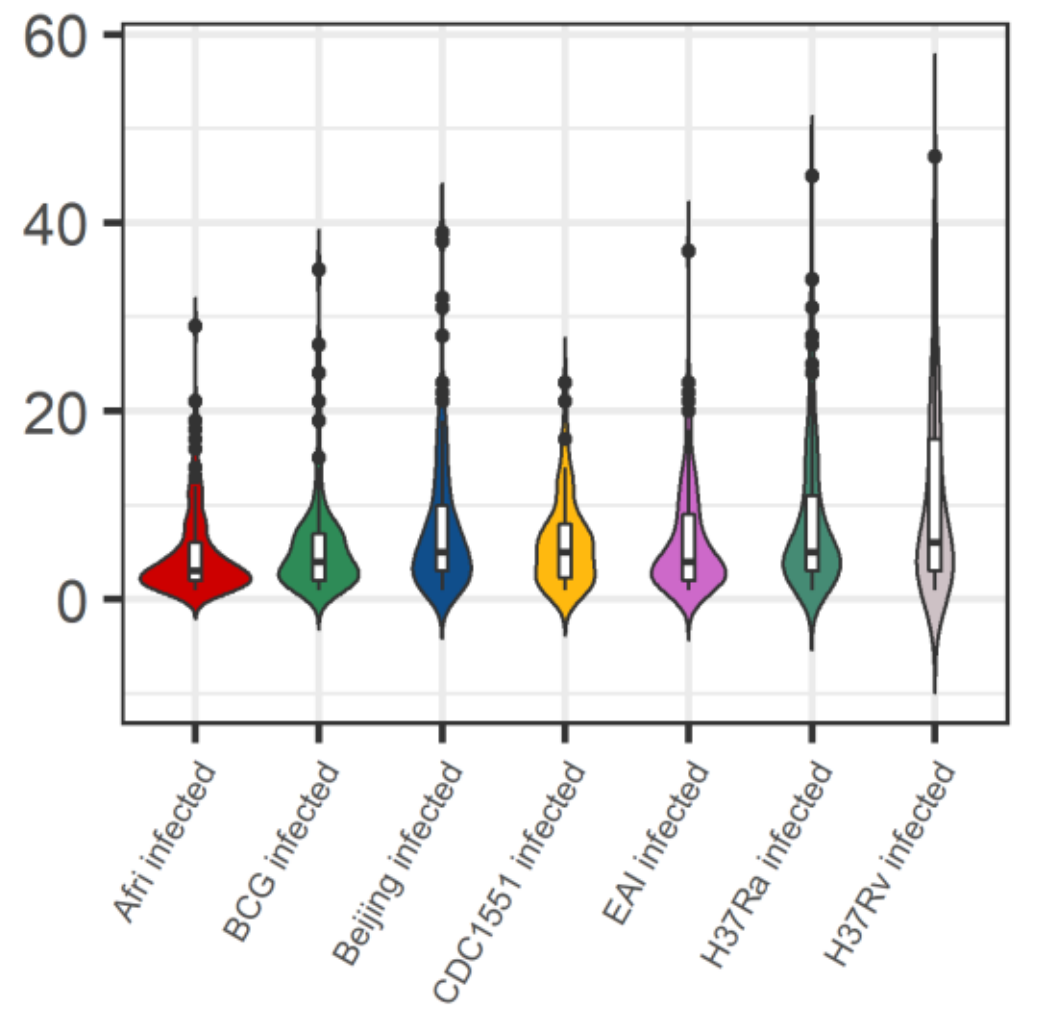

Par	Value	Std.Error	p.value
(Intercept)	1.126	0.1674	0
groupBCG infected	0.7435	0.235	0.0025
groupBeijing infected	0.3785	0.1311	0.0055
groupCDC1551 infected	0.1261	0.1629	0.4418
groupEAI infected	0.6265	0.1691	5e-04
groupH37Ra infected	0.6752	0.186	6e-04
groupH37Rv infected	0.7453	0.1955	3e-04

Contrast	estimate	SE	p.value
Afri infected - BCG infected	-0.7435	0.235	0.0104
Afri infected - Beijing infected	-0.3785	0.1311	0.0164
Afri infected - CDC1551 infected	-0.1261	0.1629	0.6185
Afri infected - EAI infected	-0.6265	0.1691	0.0042
Afri infected - H37Ra infected	-0.6752	0.186	0.0042
Afri infected - H37Rv infected	-0.7453	0.1955	0.0042
BCG infected - Beijing infected	0.365	0.2331	0.1984
BCG infected - CDC1551 infected	0.6174	0.2601	0.0488
BCG infected - EAI infected	0.117	0.1746	0.6634
BCG infected - H37Ra infected	0.0684	0.271	0.8669
BCG infected - H37Rv infected	-0.0018	0.2748	0.9949
Beijing infected - CDC1551 infected	0.2524	0.1509	0.1744
Beijing infected - EAI infected	-0.248	0.1672	0.215
Beijing infected - H37Ra infected	-0.2966	0.1765	0.1744
Beijing infected - H37Rv infected	-0.3667	0.18	0.0968
CDC1551 infected - EAI infected	-0.5004	0.2047	0.0461
CDC1551 infected - H37Ra infected	-0.549	0.1879	0.0164
CDC1551 infected - H37Rv infected	-0.6192	0.1915	0.0104
EAI infected - H37Ra infected	-0.0486	0.2197	0.8669
EAI infected - H37Rv infected	-0.1187	0.2246	0.7399
H37Ra infected - H37Rv infected	-0.0701	0.1606	0.7748

d) Colocalization Index

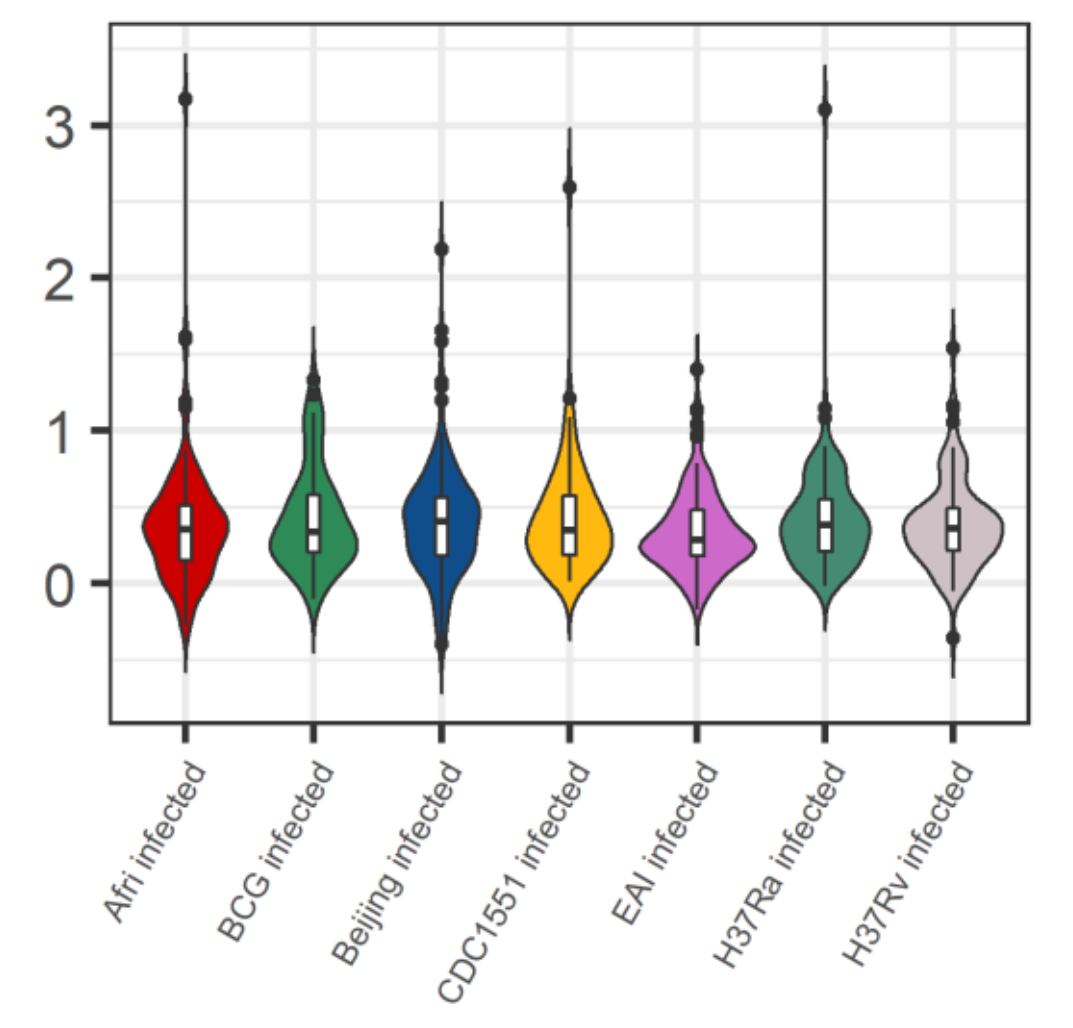

Par	Value	Std.Error	p.value
(Intercept)	-0.1201	0.0896	0.1804
groupBCG infected	0.1305	0.1528	0.3968
groupBeijing infected	0.1024	0.1093	0.3527
groupCDC1551 infected	0.1207	0.1284	0.3511
groupEAI infected	-0.1137	0.119	0.3433
groupH37Ra infected	0.121	0.1261	0.3414
groupH37Rv infected	0.0021	0.1336	0.9873

Contrast	estimate	SE	p.value
Afri infected - BCG infected	-0.1305	0.1528	0.6543
Afri infected - Beijing infected	-0.1024	0.1093	0.6543
Afri infected - CDC1551 infected	-0.1207	0.1284	0.6543
Afri infected - EAI infected	0.1137	0.119	0.6543
Afri infected - H37Ra infected	-0.121	0.1261	0.6543
Afri infected - H37Rv infected	-0.0021	0.1336	0.9985
BCG infected - Beijing infected	0.0281	0.1498	0.9985
BCG infected - CDC1551 infected	0.0098	0.1651	0.9985
BCG infected - EAI infected	0.2442	0.1298	0.4907
BCG infected - H37Ra infected	0.0095	0.1599	0.9985
BCG infected - H37Rv infected	0.1283	0.1637	0.6543
Beijing infected - CDC1551 infected	-0.0183	0.1235	0.9985
Beijing infected - EAI infected	0.2161	0.1154	0.4907
Beijing infected - H37Ra infected	-0.0185	0.1226	0.9985
Beijing infected - H37Rv infected	0.1003	0.1274	0.6543
CDC1551 infected - EAI infected	0.2344	0.1374	0.4907
CDC1551 infected - H37Ra infected	-3e-04	0.1352	0.9985
CDC1551 infected - H37Rv infected	0.1186	0.1404	0.6543
EAI infected - H37Ra infected	-0.2346	0.1318	0.4907
EAI infected - H37Rv infected	-0.1158	0.1365	0.6543
H37Ra infected - H37Rv infected	0.1188	0.1235	0.6543

ANALYSIS ON THE NUMBER OF LYSOSENSOR-POSITIVE COMPARTMENTS

GEO_HOST_adaptation, M1-day=1

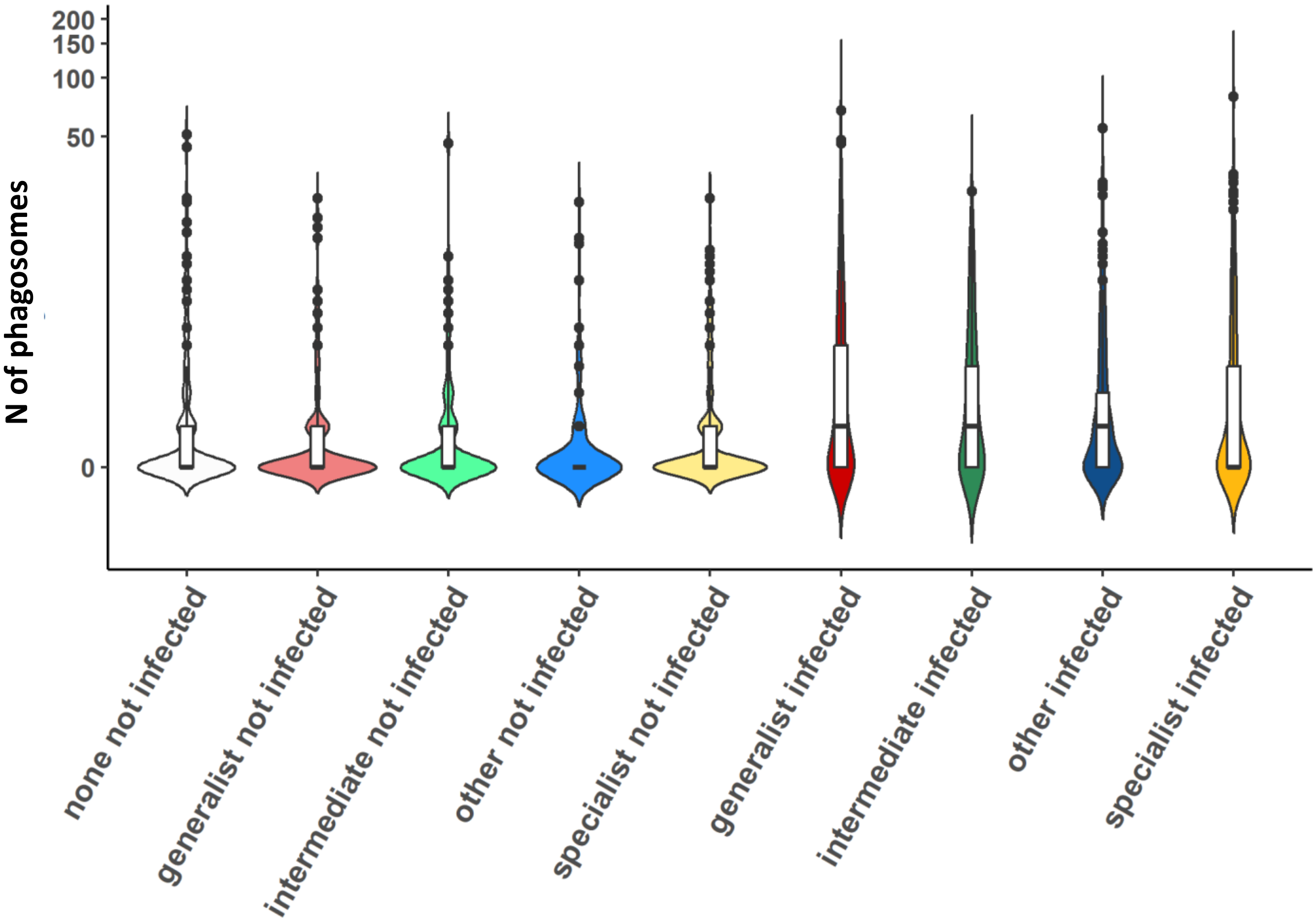

Zero-inflated Mixed-effects Poisson model
Random effects were defined nested with respect to EXP/well

Conditional model

	**Estimate**	**SE**	**p-value**
Intercept	0.836	0.276	0.0025
groupgeneralist not infected	-0.252	0.2359	0.2855
groupintermediate not infected	-0.1189	0.3026	0.6944
groupother not infected	-0.1857	0.2745	0.4988
groupspecialist not infected	0.1435	0.2552	0.5739
groupgeneralist infected	0.3905	0.2296	0.0889
groupintermediate infected	0.2791	0.293	0.3408
groupother infected	0.1793	0.262	0.4938
groupspecialist infected	0.9579	0.2492	0.0001

Zero-inflation model

	**Estimate**	**SE**	**p-value**
Intercept	0.623	0.3462	0.0719
groupgeneralist not infected	0.388	0.3098	0.2104
groupintermediate not infected	0.4105	0.3952	0.2989
groupother not infected	0.9999	0.3517	0.0045
groupspecialist not infected	0.0701	0.3225	0.8279
groupgeneralist infected	-1.0992	0.3073	3e-04
groupintermediate infected	-0.7924	0.3911	0.0428
groupother infected	-0.5407	0.34	0.1118
groupspecialist infected	-0.7732	0.3181	0.0151

**Contrast**	**estimate**	**SE**	**p-value***
none not infected-generalist not infected	0.252	0.2359	0.5709
none not infected-intermediate not infected	0.1189	0.3026	0.7936
none not infected-other not infected	0.1857	0.2745	0.7041
none not infected-specialist not infected	-0.1435	0.2552	0.7652
none not infected-generalist infected	-0.3905	0.2296	0.2135
none not infected-intermediate infected	-0.2791	0.293	0.6162
none not infected-other infected	-0.1793	0.262	0.7041
**none not infected-specialist infected**	**-0.9579**	**0.2492**	**0.001**
generalist not infected-intermediate not infected	-0.1331	0.28	0.7936
generalist not infected-other not infected	-0.0663	0.2621	0.8352
generalist not infected-specialist not infected	-0.3955	0.2309	0.2135
intermediate not infected-other not infected	0.0668	0.3263	0.8377
intermediate not infected-specialist not infected	-0.2624	0.3007	0.6162
other not infected-specialist not infected	-0.3292	0.2611	0.4526
generalist infected-intermediate infected	0.1114	0.2636	0.7936
generalist infected-other infected	0.2112	0.2432	0.6162
**generalist infected-specialist infected**	**-0.5674**	**0.2175**	**0.0312**
intermediate infected-other infected	0.0998	0.3064	0.8124
intermediate infected-specialist infected	-0.6788	0.285	0.0517
**other infected-specialist infected**	**-0.7786**	**0.2412**	**0.005**
**generalist not infected-generalist infected**	**-0.6425**	**0.07**	**<0.0001**
**intermediate not infected-intermediate infected**	**-0.398**	**0.1062**	**0.0011**
**other not infected-other infected**	**-0.365**	**0.1108**	**0.0047**
**specialist not infected-specialist infected**	**-0.8144**	**0.0745**	**<0.0001**

* False discovery rate (FDR) correction

GEO_HOST_adaptation, M1-day=2

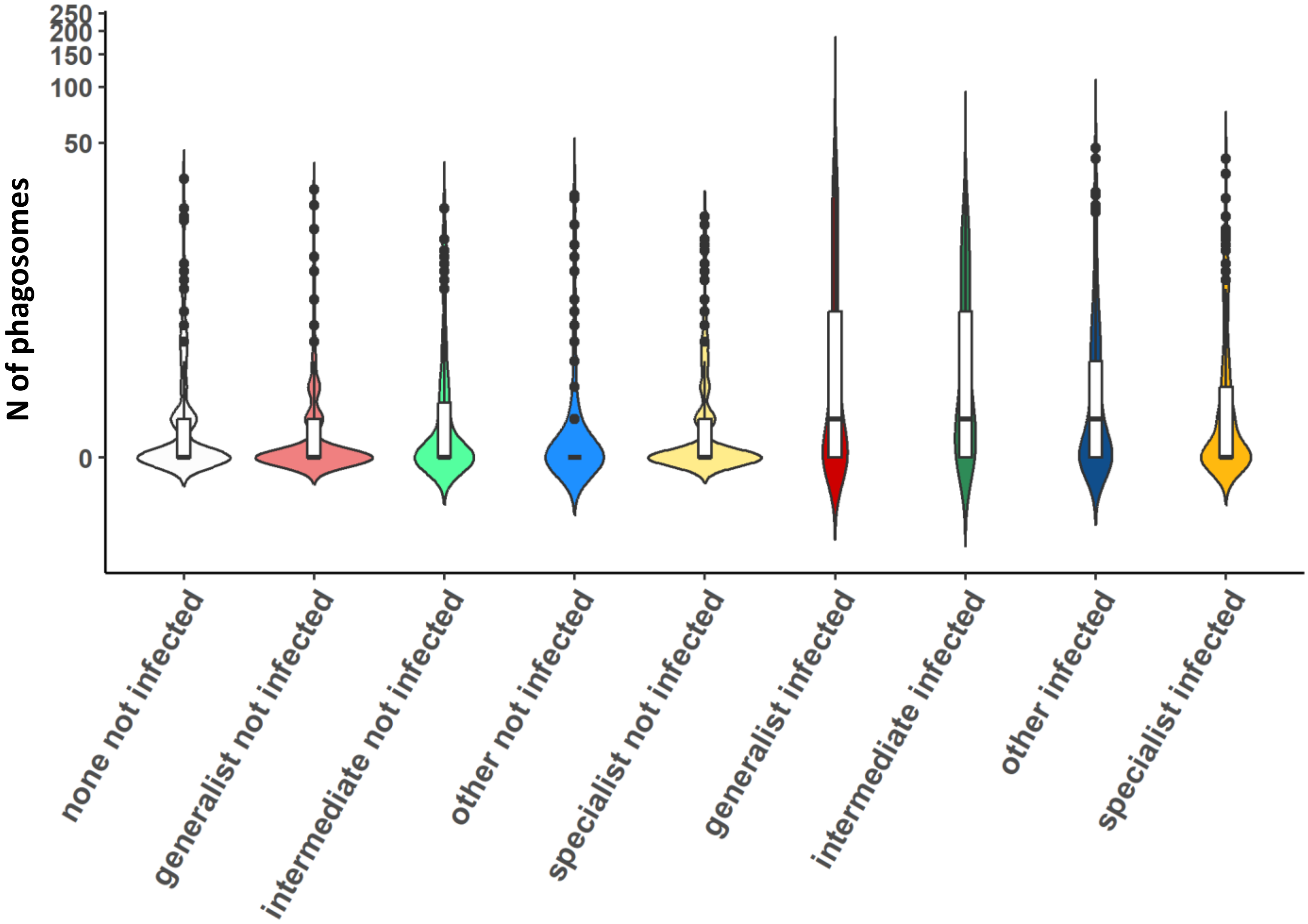

Zero-inflated Mixed-effects Poisson model
Random effects were defined nested with respect to EXP/well

Conditional model

	**Estimate**	**SE**	**p-value**
Intercept	0.7967	0.2426	0.001
groupgeneralist not infected	-0.024	0.2663	0.928
groupintermediate not infected	0.5369	0.3227	0.0961
groupother not infected	0.4789	0.2945	0.104
groupspecialist not infected	0.3259	0.2741	0.2345
groupgeneralist infected	1.2219	0.2559	<0.0001
groupintermediate infected	0.8383	0.3151	0.0078
groupother infected	0.8774	0.2848	0.0021
groupspecialist infected	0.5818	0.2715	0.0321

Zero-inflation model

	**Estimate**	**SE**	**p-value**
Intercept	0.3109	0.2958	0.2933
groupgeneralist not infected	0.4307	0.307	0.1606
groupintermediate not infected	0.4463	0.3747	0.2335
groupother not infected	0.9704	0.3463	0.0051
groupspecialist not infected	0.2976	0.2986	0.3189
groupgeneralist infected	-0.7279	0.2837	0.0103
groupintermediate infected	-1.0123	0.3718	0.0065
groupother infected	-0.3559	0.3117	0.2535
groupspecialist infected	-0.3045	0.2951	0.3021

**Contrast**	**estimate**	**SE**	**p-value***
none not infected-generalist not infected	0.024	0.2663	0.928
none not infected-intermediate not infected	-0.5369	0.3227	0.1919
none not infected-other not infected	-0.4789	0.2945	0.1919
none not infected-specialist not infected	-0.3259	0.2741	0.3142
**none not infected-generalist infected**	**-1.2219**	**0.2559**	**<0.0001**
**none not infected-intermediate infected**	**-0.8383**	**0.3151**	**0.0234**
**none not infected-other infected**	**-0.8774**	**0.2848**	**0.0099**
none not infected-specialist infected	-0.5818	0.2715	0.0856
generalist not infected-intermediate not infected	-0.5609	0.2985	0.133
generalist not infected-other not infected	-0.5029	0.2684	0.133
generalist not infected-specialist not infected	-0.35	0.2356	0.2357
intermediate not infected-other not infected	0.058	0.3358	0.928
intermediate not infected-specialist not infected	0.211	0.3121	0.5989
other not infected-specialist not infected	0.1529	0.2635	0.6419
generalist infected-intermediate infected	0.3836	0.2806	0.2575
generalist infected-other infected	0.3445	0.2456	0.2573
**generalist infected-specialist infected**	**0.6401**	**0.2198**	**0.0144**
intermediate infected-other infected	-0.0391	0.3197	0.928
intermediate infected-specialist infected	0.2565	0.3022	0.5002
other infected-specialist infected	0.2956	0.2493	0.3142
**generalist not infected-generalist infected**	**-1.2459**	**0.0847**	**<0.0001**
**intermediate not infected-intermediate infected**	**-0.3014**	**0.1102**	**0.0213**
**other not infected-other infected**	**-0.3986**	**0.0943**	**0.0002**
**specialist not infected-specialist infected**	**-0.2559**	**0.0736**	**0.003**

* False discovery rate (FDR) correction

GEO_HOST_adaptation, M2-day=1

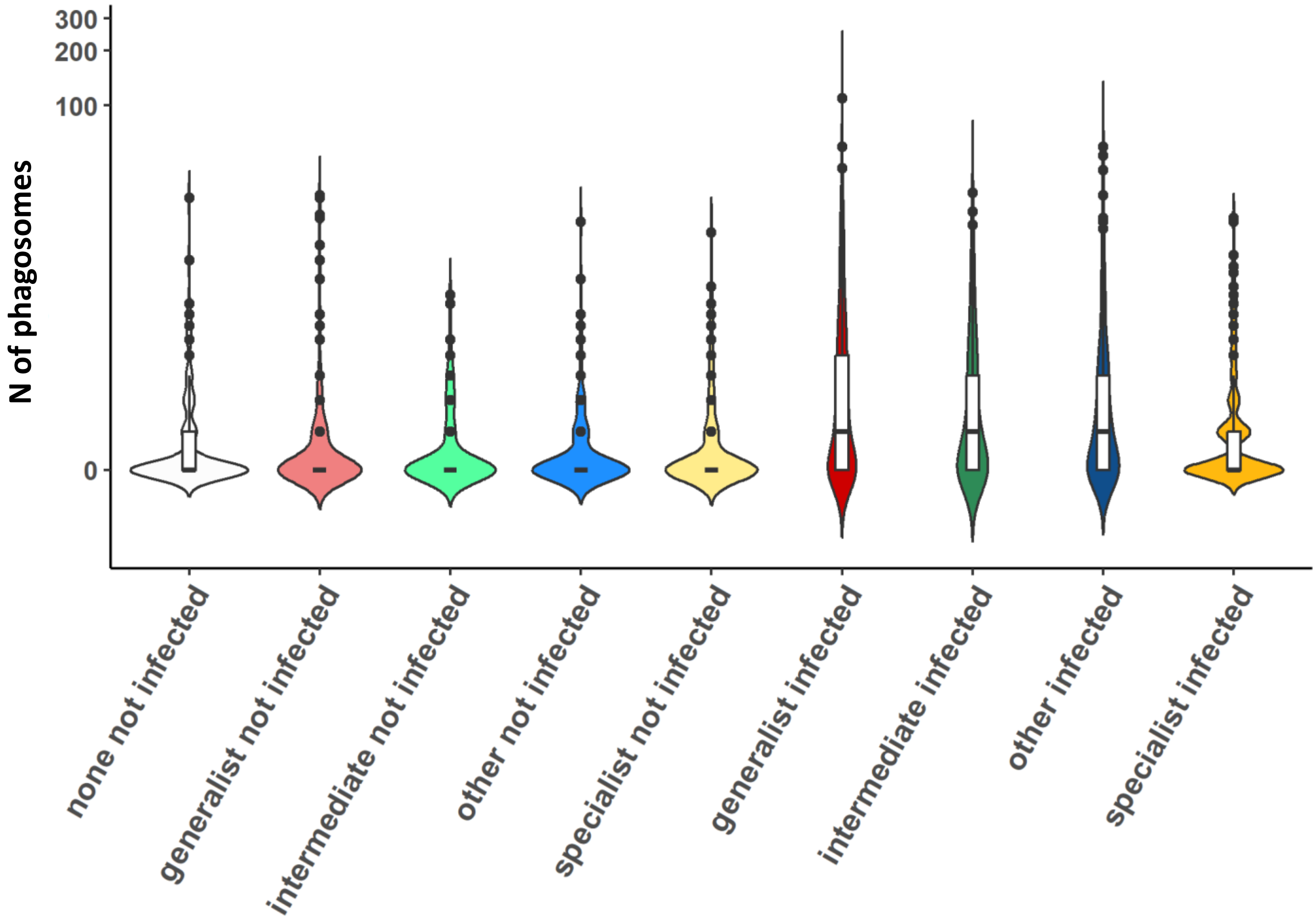

Zero-inflated Mixed-effects Poisson model
Random effects were defined nested with respect to EXP/well

Conditional model

	Estimate	SE	p-value
Intercept	0.5071	0.2957	0.0863
groupgeneralist not infected	0.2814	0.2841	0.3219
groupintermediate not infected	-0.3785	0.3625	0.2963
groupother not infected	-0.1204	0.3034	0.6914
groupspecialist not infected	-0.0118	0.3037	0.969
groupgeneralist infected	0.8031	0.2782	0.0039
groupintermediate infected	0.688	0.3403	0.0432
groupother infected	0.8053	0.2886	0.0053
groupspecialist infected	0.3975	0.2975	0.1815

Zero-inflation model

	Estimate	SE	p-value
Intercept	0.88	0.3608	0.0147
groupgeneralist not infected	0.3193	0.2765	0.2482
groupintermediate not infected	0.4372	0.348	0.209
groupother not infected	0.2471	0.2982	0.4074
groupspecialist not infected	-0.0445	0.2904	0.8783
groupgeneralist infected	-1.2474	0.2695	<0.0001
groupintermediate infected	-0.7226	0.3164	0.0224
groupother infected	-1.0517	0.2833	0.0002
groupspecialist infected	-0.9201	0.2875	0.0014

Contrast	estimate	SE	p-value*
none not infected-generalist not infected	-0.2814	0.2841	0.4829
none not infected-intermediate not infected	0.3785	0.3625	0.4741
none not infected-other not infected	0.1204	0.3034	0.78
none not infected-specialist not infected	0.0118	0.3037	0.9932
**none not infected-generalist infected**	**-0.8031**	**0.2782**	**0.0187**
none not infected-intermediate infected	-0.688	0.3403	0.1296
**none not infected-other infected**	**-0.8053**	**0.2886**	**0.0211**
none not infected-specialist infected	-0.3975	0.2975	0.3629
generalist not infected-intermediate not infected	0.66	0.3059	0.1062
generalist not infected-other not infected	0.4018	0.2783	0.3246
generalist not infected-specialist not infected	0.2932	0.2522	0.4524
intermediate not infected-other not infected	-0.2581	0.3579	0.6278
intermediate not infected-specialist not infected	-0.3667	0.3371	0.4741
other not infected-specialist not infected	-0.1086	0.277	0.78
generalist infected-intermediate infected	0.1151	0.273	0.78
generalist infected-other infected	-0.0022	0.2555	0.9932
generalist infected-specialist infected	0.4056	0.2378	0.2347
intermediate infected-other infected	-0.1173	0.3213	0.78
intermediate infected-specialist infected	0.2905	0.306	0.4834
other infected-specialist infected	0.4078	0.2529	0.2564
**generalist not infected-generalist infected**	**-0.5217**	**0.0701**	**<0.0001**
**intermediate not infected-intermediate infected**	**-1.0665**	**0.1467**	**<0.0001**
**other not infected-other infected**	**-0.9257**	**0.1112**	**<0.0001**
**specialist not infected-specialist infected**	**-0.4093**	**0.0948**	**0.0001**

* False discovery rate (FDR) correction

GEO_HOST_adaptation, M2-day=2

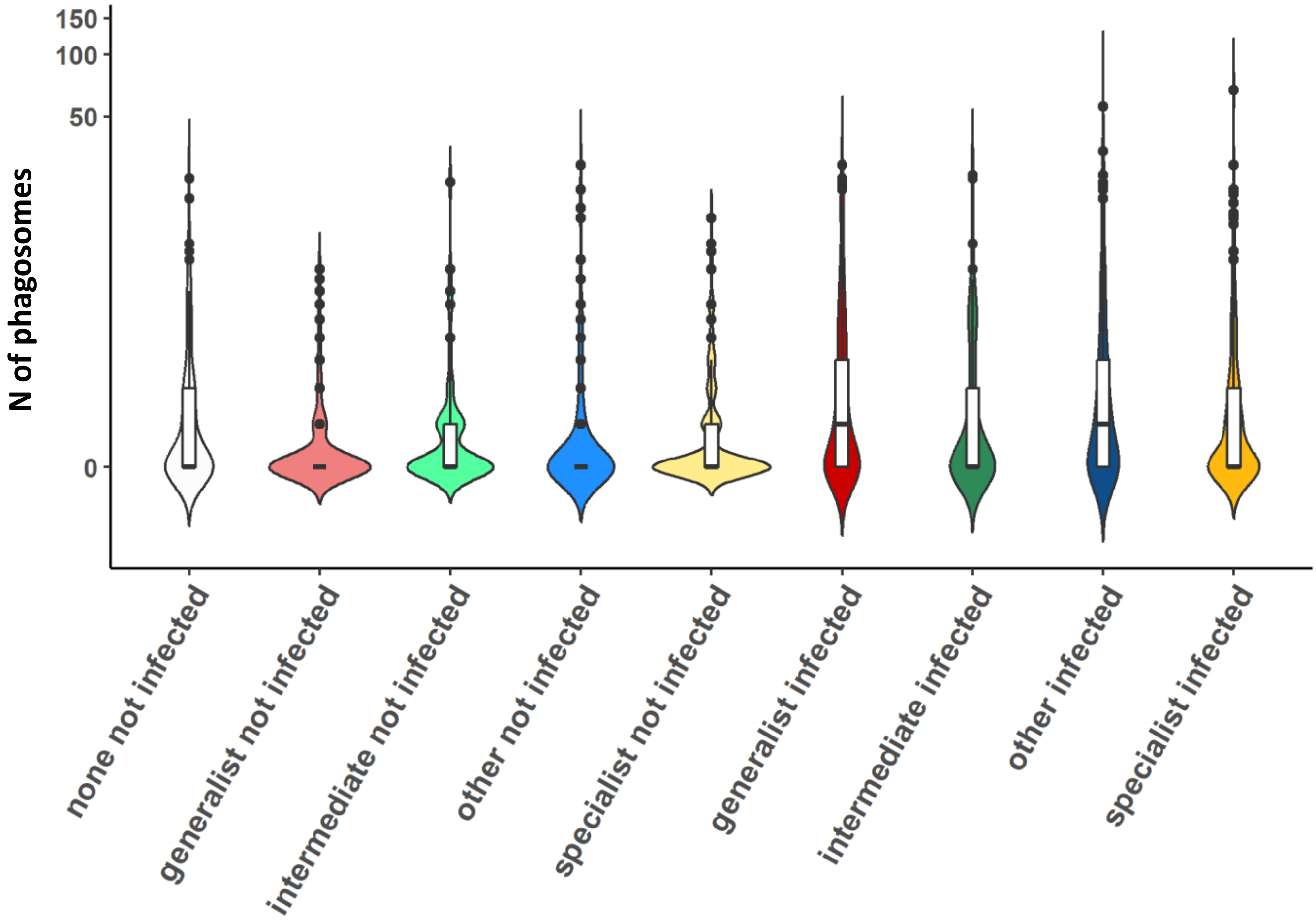

Zero-inflated Mixed-effects Poisson model
Random effects were defined nested with respect to EXP/well

Conditional model

	Estimate	SE	p-value
Intercept	1.0011	0.2105	<0.0001
groupgeneralist not infected	-0.4274	0.2408	0.0759
groupintermediate not infected	-0.3705	0.293	0.2061
groupother not infected	0.1869	0.2657	0.4817
groupspecialist not infected	-0.2388	0.2496	0.3386
groupgeneralist infected	0.3822	0.2184	0.0801
groupintermediate infected	0.3787	0.2784	0.1738
groupother infected	0.358	0.256	0.162
groupspecialist infected	0.2613	0.2413	0.2789

Zero-inflation model

	Estimate	SE	p-value
Intercept	0.3905	0.3332	0.2411
groupgeneralist not infected	0.577	0.2816	0.0404
groupintermediate not infected	0.2645	0.3391	0.4354
groupother not infected	0.6891	0.295	0.0195
groupspecialist not infected	0.2852	0.2667	0.2849
groupgeneralist infected	-0.6093	0.2499	0.0148
groupintermediate infected	0.3193	0.3171	0.3139
groupother infected	-0.8391	0.2757	0.0023
groupspecialist infected	-0.2514	0.2539	0.3222

**Contrast**	**estimate**	**SE**	**p-value**
none not infected-generalist not infected	0.4274	0.2408	0.2137
none not infected-intermediate not infected	0.3705	0.293	0.4122
none not infected-other not infected	-0.1869	0.2657	0.7226
none not infected-specialist not infected	0.2388	0.2496	0.5805
none not infected-generalist infected	-0.3822	0.2184	0.2137
none not infected-intermediate infected	-0.3787	0.2784	0.3791
none not infected-other infected	-0.358	0.256	0.3791
none not infected-specialist infected	-0.2613	0.2413	0.5149
generalist not infected-intermediate not infected	-0.0569	0.2881	0.9639
generalist not infected-other not infected	-0.6143	0.2555	0.0972
generalist not infected-specialist not infected	-0.1885	0.2386	0.6871
intermediate not infected-other not infected	-0.5574	0.3102	0.2137
intermediate not infected-specialist not infected	-0.1316	0.2999	0.808
other not infected-specialist not infected	0.4258	0.2426	0.2137
generalist infected-intermediate infected	0.0035	0.2536	0.9891
generalist infected-other infected	0.0242	0.223	0.9834
generalist infected-specialist infected	0.1209	0.2061	0.787
intermediate infected-other infected	0.0208	0.2875	0.9834
intermediate infected-specialist infected	0.1174	0.2786	0.808
other infected-specialist infected	0.0967	0.2242	0.808
**generalist not infected-generalist infected**	**-0.8096**	**0.1212**	**<0.0001**
**intermediate not infected-intermediate infected**	**-0.7492**	**0.1509**	**<0.0001**
other not infected-other infected	-0.171	0.0921	0.2137
**specialist not infected-specialist infected**	**-0.5001**	**0.0905**	**<0.0001**

* False discovery rate (FDR) correction
