# Supplementary material for: Mycobacterium tuberculosis diversity and macrophage heterogeneity dictate phagosomal acidification
Source: Microbiol Spectr. 2026 Jul 20;14(9):e03762-25. doi: 10.1128/spectrum.03762-25 (PMC13532232; doi:10.1128/spectrum.03762-25)
Supplement: Supplementary file 3 [file spectrum.03762-25-s0005.pdf]

SUPPLEMENTARY 3

Number of mycobacteria internalized per cell (M/C) over time

Comparison of M/C observed between time1 (6h p.i.) and time2 (24h p.i.)

GEO HOST ADAPTATION, M1

Par	Value	Std.Error	p.value
(Intercept)	-0.1346	0.1234	0.2754
groupintermediate infected	-0.0708	0.1359	0.6043
groupother infected	0.1237	0.1239	0.3219
groupspecialist infected	-0.0134	0.1083	0.9021
time2	0.1794	0.0728	0.0139
groupintermediate infected:time2	0.2195	0.1423	0.1232
groupother infected:time2	0.3517	0.1179	0.0029
groupspecialist infected:time2	-0.201	0.1048	0.0553

contrast	group	estimate	SE	p.value
time1 - time2	generalist infected	-0.1794	0.0728	0.0139
time1 - time2	intermediate infected	-0.3989	0.1222	0.0011
time1 - time2	other infected	-0.5312	0.0931	<0.0001
time1 - time2	specialist infected	0.0216	0.0753	0.7748

GEO HOST ADAPTATION, M2

Par	Value	Std.Error	p.value
(Intercept)	-0.0873	0.1289	0.4985
groupintermediate infected	-0.0647	0.1302	0.6209
groupother infected	0.0188	0.117	0.8728
groupspecialist infected	0.0851	0.1044	0.4181
time2	0.2436	0.0739	0.001
groupintermediate infected:time2	-0.2991	0.1456	0.0401
groupother infected:time2	-0.0636	0.1157	0.5824
groupspecialist infected:time2	-0.3512	0.1051	0.0008

contrast	group	estimate	SE	p.value
time1 - time2	generalist infected	-0.2436	0.0739	0.001
time1 - time2	intermediate infected	0.0555	0.1256	0.6585
time1 - time2	other infected	-0.18	0.0894	0.0442
time1 - time2	specialist infected	0.1076	0.0747	0.1501

COMPARISON M1 vs M2

Acidification Mean

Statistical method 1: Linear quantile mixed models (LQMM) on the median were applied to test the difference between M1 and M2, accounting for group membership.

Time 1

	Value	Std. Error	p-value
Intercept	0.8125	0.1582	<0.0001
groupgeneralist not infected	0.0737	0.0960	0.4461
groupintermediate not infected	0.1759	0.0846	0.0428
groupother not infected	-0.1229	0.1001	0.2258
groupspecialist not infected	0.2224	0.0858	0.0126
groupgeneralist infected	0.2114	0.1092	0.0586
groupintermediate infected	0.3325	0.0956	0.0011
groupother infected	-0.0058	0.1000	0.9539
groupspecialist infected	0.3908	0.0842	0.0000
M2	0.2244	0.1469	0.1330

M2 values are not significantly different from the M1 values (p=0.133).

Time 2

	Value	Std. Error	p-value
Intercept	1.0034	0.0266	<0.0001
groupgeneralist not infected	-0.0037	0.0357	0.9188
groupintermediate not infected	-0.0771	0.0394	0.0561
groupother not infected	0.0646	0.0541	0.2384
groupspecialist not infected	0.1043	0.0266	0.0003
groupgeneralist infected	0.1422	0.0405	0.0010
groupintermediate infected	0.0865	0.0551	0.1230
groupother infected	0.1805	0.0558	0.0022
groupspecialist infected	0.2150	0.0298	0.0000
M2	-0.0109	0.0359	0.7627

M2 values are not significantly different from the M1 values (p=0.7627).

Statistical method 2: Linear quantile mixed models (LQMM) on the median were applied to assess the difference between M1 and M2 within each group.

Time 1

	Value	Std. Error	p-value
Intercept	0.9152	0.1184	<0.0001
groupgeneralist not infected	-0.0003	0.1209	0.9978
groupintermediate not infected	-0.1275	0.1129	0.2642
groupother not infected	-0.1929	0.1375	0.1668
groupspecialist not infected	0.2745	0.1447	0.0637
groupgeneralist infected	0.1042	0.1349	0.4438

groupintermediate infected	0.0231	0.1188	0.8469
groupother infected	-0.1062	0.1329	0.4278
groupspecialist infected	0.4701	0.1537	0.0036
M2	0.1071	0.1079	0.3261
groupgeneralist not infected:M2	0.2334	0.1656	0.1650
groupintermediate not infected:M2	0.3555	0.1096	0.0021
groupother not infected:M2	0.1829	0.1429	0.2066
groupspecialist not infected:M2	-0.0619	0.1295	0.6350
groupgeneralist infected:M2	0.3084	0.1962	0.1225
groupintermediate infected:M2	0.3697	0.1272	0.0055
groupother infected:M2	0.2560	0.1530	0.1007
groupspecialist infected:M2	-0.1236	0.1398	0.3811

M2 values were significantly different from M1 values in groups intermediate not infected and intermediate infected, while no significant differences were observed in the other groups.

Time 2

	Value	Std. Error	p-value
(Intercept)	0.9919	0.0242	< 0.0001
groupgeneralist not infected	0.0119	0.0433	0.7848
groupintermediate not infected	0.0221	0.0520	0.6730
groupother not infected	-0.0653	0.0517	0.2128
groupspecialist not infected	0.1401	0.0415	0.0015
groupgeneralist infected	0.1766	0.0658	0.0099
groupintermediate infected	0.2892	0.0729	0.0002
groupother infected	0.0730	0.0588	0.2207
groupspecialist infected	0.2641	0.0444	0.0000
M2	0.0106	0.0332	0.7518
groupgeneralist not infected:M2	-0.0384	0.0639	0.5507
groupintermediate not infected:M2	-0.1111	0.0730	0.1341
groupother not infected:M2	0.1677	0.0881	0.0629
groupspecialist not infected:M2	-0.0465	0.0663	0.4863
groupgeneralist infected:M2	-0.1028	0.0792	0.2005
groupintermediate infected:M2	-0.3102	0.0794	0.0003
groupother infected:M2	0.1416	0.1067	0.1906
groupspecialist infected:M2	-0.0552	0.0677	0.4191

M2 values were significantly different from M1 values in groups intermediate infected, while no significant differences were observed in the other groups.

Number of cells

Statistical method 1: Logistic mixed-effects models were applied to test the difference between M1 and M2 in the percentage of cells showing acidification, accounting for group membership.

Time 1

	Estimate	se	OR	p-value
Intercept	-3.7842	0.5571	0.0227	<0.0001
groupgeneralist not infected	1.1209	0.5041	3.0676	0.0262
groupintermediate not infected	0.5415	0.6702	1.7185	0.4192
groupother not infected	0.7893	0.5811	2.2019	0.1744
groupspecialist not infected	1.8373	0.4994	6.2793	0.0002
groupgeneralist infected	2.6184	0.4955	13.7142	<0.0001
groupintermediate infected	2.1332	0.6457	8.4415	0.001
groupother infected	1.827	0.5671	6.2155	0.0013
groupspecialist infected	2.8635	0.4992	17.5222	<0.0001
M2	0.8475	0.3018	2.3338	0.005

A significant difference in the proportion of acidifying cells was observed between M1 and M2 (0.005).

Time 2

	Estimate	se	OR	p-value
Intercept	-3.1748	0.4154	0.0418	<0.0001
groupgeneralist not infected	0.6032	0.4181	1.8279	0.1491
groupintermediate not infected	-0.2386	0.6771	0.7877	0.7245
groupother not infected	1.0186	0.4377	2.7693	0.0200
groupspecialist not infected	0.6341	0.4030	1.8853	0.1157
groupgeneralist infected	1.6096	0.3937	5.0006	<0.0001
groupintermediate infected	1.6095	0.5028	5.0003	0.0014
groupother infected	1.9249	0.4217	6.8544	<0.0001
groupspecialist infected	1.5695	0.3910	4.8045	0.0001
M2	0.0117	0.1991	1.0118	0.9532

No significant difference in the proportion of acidifying cells was observed between M1 and M2.

Statistical method 2: Logistic mixed-effects models were applied to test the difference between M1 and M2 within each group.

Time 1

	Estimate	se	OR	p-value
Intercept	-3.3699	0.6497	0.0344	<0.0001
groupgeneralist not infected	0.0635	0.7231	1.0655	0.9301
groupintermediate not infected	-0.5919	0.9997	0.5533	0.5538
groupother not infected	0.8454	0.7901	2.3289	0.2846
groupspecialist not infected	1.7948	0.6766	6.0184	0.008
groupgeneralist infected	1.5876	0.695	4.892	0.0224
groupintermediate infected	1.1566	0.8967	3.179	0.1971
groupother infected	1.5115	0.7781	4.5333	0.0521
groupspecialist infected	2.9336	0.677	18.7945	<0.0001

M2	0.1024	0.7596	1.1079	0.8927
groupgeneralist not infected:M2	1.9428	0.9801	6.978	0.0475
groupintermediate not infected:M2	2.0135	1.2882	7.4897	0.118
groupother not infected:M2	-0.1649	1.0789	0.848	0.8785
groupspecialist not infected:M2	0.002	0.9402	1.002	0.9983
groupgeneralist infected:M2	1.9113	0.9547	6.7622	0.0453
groupintermediate infected:M2	1.8178	1.199	6.1583	0.1295
groupother infected:M2	0.5581	1.0481	1.7473	0.5944
groupspecialist infected:M2	-0.2412	0.9401	0.7857	0.7975

The probability of acidification was significantly different between M1 and M2 in the generalist not Infected and generalist Infected groups, while no significant differences were observed in the other groups.

Time 2

	Estimate	se	OR	p-value
Intercept	-3.1815	0.5058	0.0415	<0.0001
groupgeneralist not infected	0.4609	0.5746	1.5856	0.4224
groupintermediate not infected	0.3962	0.815	1.4862	0.6269
groupother not infected	0.2905	0.6173	1.3371	0.6379
groupspecialist not infected	0.5087	0.5395	1.6631	0.3457
groupgeneralist infected	2.0166	0.5151	7.5124	0.0001
groupintermediate infected	2.3323	0.6226	10.3013	0.0002
groupother infected	1.1574	0.559	3.1816	0.0384
groupspecialist infected	1.663	0.5156	5.2751	0.0013
M2	0.0421	0.6478	1.043	0.9482
groupgeneralist not infected:M2	0.2377	0.7987	1.2683	0.766
groupintermediate not infected:M2	-1.3997	1.4084	0.2467	0.3203
groupother not infected:M2	1.3791	0.8256	3.9714	0.0949
groupspecialist not infected:M2	0.256	0.759	1.2917	0.7359
groupgeneralist infected:M2	-0.8395	0.7401	0.4319	0.2566
groupintermediate infected:M2	-1.7714	0.9715	0.1701	0.0683
groupother infected:M2	1.4896	0.7768	4.4352	0.0551
groupspecialist infected:M2	-0.1638	0.7323	0.8489	0.823

No significant difference in the proportion of acidifying cells was observed between M1 and M2.

Colocalization index

Statistical method 1: Linear mixed-effects models were applied to test the difference between M1 and M2, accounting for group membership.

Time 1 (Ordered Quantile normalization transformation)

	Value	Std.Error	p-value
Intercept	0.1125	0.0645	0.0815
groupintermediate infected	-0.0107	0.0931	0.9088
groupother infected	0.1109	0.0842	0.1902
groupspecialist infected	-0.1970	0.0748	0.0094
M2	-0.1651	0.0560	0.0037

M2 values are significantly different from the M1 values (p=0.0037).

Time 2 (Ordered Quantile normalization transformation)

	Value	Std.Error	p-value
Intercept	0.1148	0.0748	0.1251
groupintermediate infected	-0.2001	0.1092	0.0692
groupother infected	0.0296	0.0904	0.7435
groupspecialist infected	-0.2184	0.0811	0.0080
M2	-0.0640	0.0600	0.2878

M2 values are not significantly different from the M1 values (p=0.2878).

Statistical method 2: Linear mixed-effects models were applied to test the difference between M1 and M2 within each group.

Time 1 (Ordered Quantile normalization transformation)

	Value	Std.Error	p-value
(Intercept)	0.0680	0.0754	0.3673
groupintermediate infected	0.1731	0.1295	0.1835
groupother infected	0.1407	0.1156	0.2257
groupspecialist infected	-0.1541	0.1022	0.1339
M2	-0.0779	0.0949	0.4132
groupintermediate infected:M2	-0.3662	0.1800	0.0439
groupother infected:M2	-0.0589	0.1536	0.7018
groupspecialist infected:M2	-0.0848	0.1381	0.5402

M2 values were significantly different from M1 values in group intermediate infected, while no significant differences were observed in the other groups.

Time 2 (Ordered Quantile normalization transformation)

	Value	Std.Error	p-value
Intercept	0.2052	0.0851	0.0160
groupintermediate infected	-0.4874	0.1471	0.0012
groupother infected	-0.0520	0.1184	0.6610
groupspecialist infected	-0.3424	0.1074	0.0018
M2	-0.2564	0.1024	0.0135
groupintermediate infected:M2	0.5763	0.2039	0.0055
groupother infected:M2	0.1825	0.1579	0.2500
groupspecialist infected:M2	0.2632	0.1440	0.0699

M2 values were significantly different from M1 values in groups generalist infected and intermediate infected, while no significant differences were observed in the other groups.

N of mycobacteria per cell (M/C)

Statistical method 1: Linear mixed-effects models were applied to test the difference between M1 and M2, accounting for group membership.

Time 1 (Ordered Quantile normalization transformation)

	Value	Std.Error	p-value
Intercept	0.0064	0.1018	0.9498
groupintermediate infected	-0.1199	0.1014	0.2393
groupother infected	-0.0270	0.0945	0.7752
groupspecialist infected	-0.0202	0.0830	0.8086
M2	0.0091	0.0589	0.8775

M2 values are not significantly different from the M1 values (p=0.8775).

Time 2 (Ordered Quantile normalization transformation)

	Value	Std.Error	p-value
(Intercept)	0.0738	0.1102	0.5031
groupintermediate infected	-0.0756	0.1033	0.4656
groupother infected	0.2127	0.0901	0.0197
groupspecialist infected	-0.1456	0.0801	0.0714
M2	-0.1149	0.0566	0.0442

M2 values are significantly different from the M1 values (p=0.0442).

Statistical method 2: Linear mixed-effects models were applied to assess the difference between M1 and M2 within each group.

Time 1

	Value	Std.Error	p-value
Intercept	0.0192	0.1103	0.8622
groupintermediate infected	-0.1487	0.1420	0.2970
groupother infected	-0.0180	0.1273	0.8877
groupspecialist infected	-0.0536	0.1115	0.6318
M2	-0.0159	0.1019	0.8763
groupintermediate infected:M2	0.0575	0.1946	0.7682
groupother infected:M2	-0.0176	0.1647	0.9151
groupspecialist infected:M2	0.0666	0.1468	0.6510

M2 values are not significantly different from the M1 values.

Time 2

	Value	Std.Error	p-value
(Intercept)	0.0224	0.1155	0.8462
groupintermediate infected	0.0998	0.1380	0.4709
groupother infected	0.3612	0.1164	0.0024
groupspecialist infected	-0.1468	0.1041	0.1607
M2	-0.0088	0.0958	0.9267
groupintermediate infected:M2	-0.3575	0.1920	0.0648
groupother infected:M2	-0.2963	0.1515	0.0527
groupspecialist infected:M2	-0.0044	0.1360	0.9743

M2 values are not significantly different from the M1 values.
