# Supplementary material for: Mycobacterium tuberculosis diversity and macrophage heterogeneity dictate phagosomal acidification
Source: Microbiol Spectr. 2026 Jul 20;14(9):e03762-25. doi: 10.1128/spectrum.03762-25 (PMC13532232; doi:10.1128/spectrum.03762-25)
Supplement: Supplementary file 6 [file spectrum.03762-25-s0008.pdf]

SUPPLEMENTARY 6

MTB INTRAMACROPHAGE SURVIVAL

Intramacrophage MTB survival

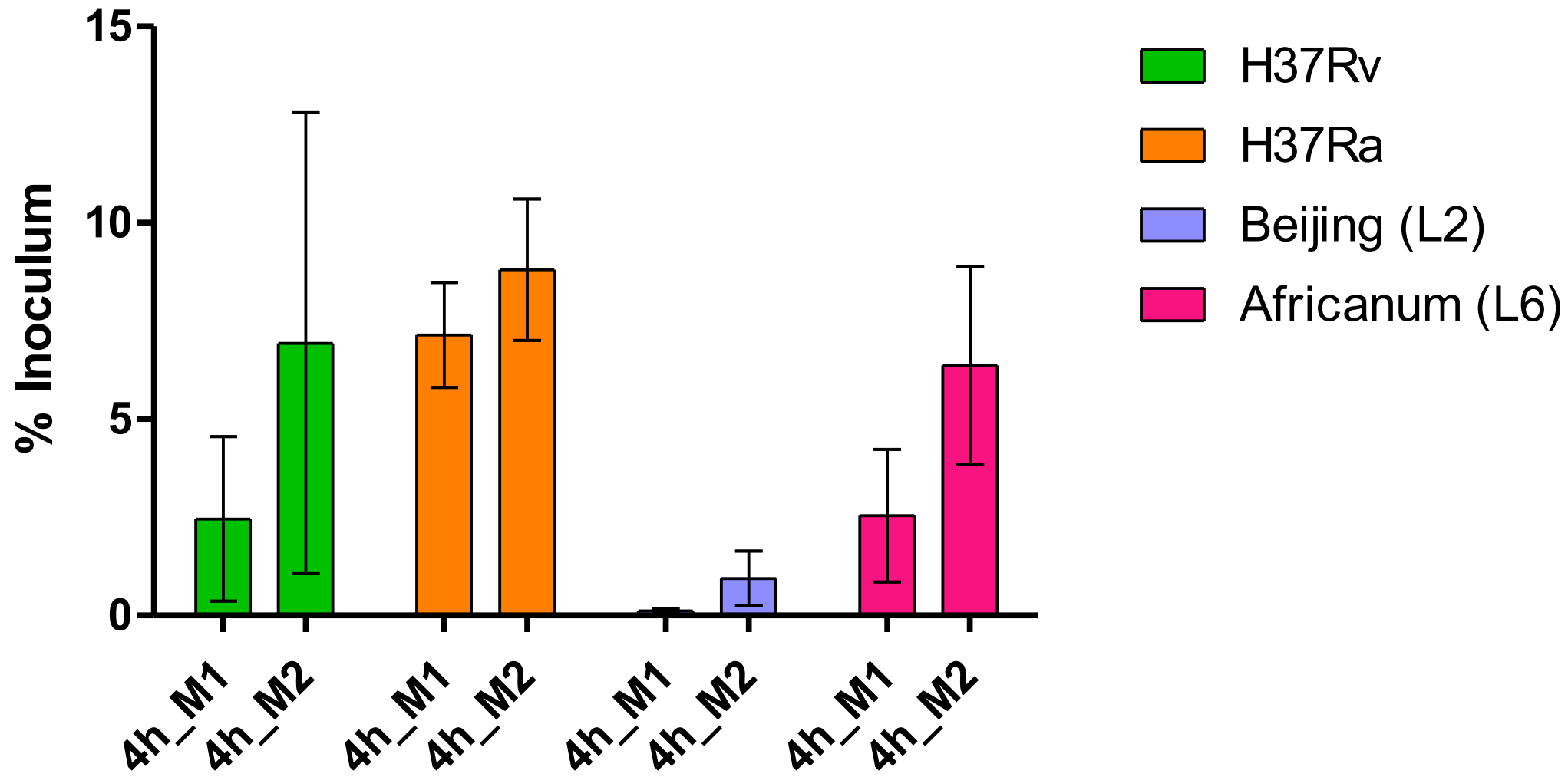

Figure S5.1: CFU results at 4h p.i. (% of the inoculum). Beijing: Generalist/L2; Africanum: Specialist/L6

Differences are present considering M1/M2 cell phenotype: entry of bacteria is increased in M2 cells in all MTB strains selected. Higher CFU levels are found in H37Ra infected cells, while H37Rv and L6 show a similar CFU level. L2 uptake resulted to be very low compared to all other strains, with CFU values close to 1% of the inoculum in M1 cells and to 3% in M2 cells.

Raw CFU counts

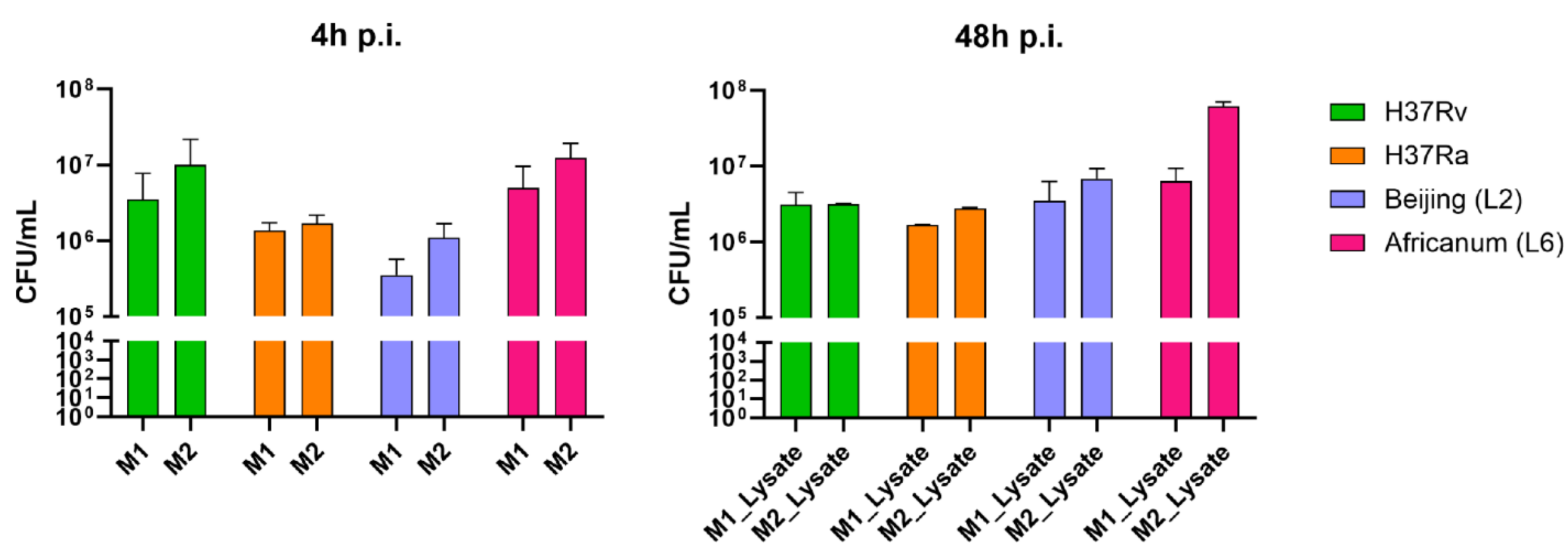

Figure S5.2: Raw intracellular CFU counts of MTB strains at 4h and 48h p.i. Raw CFU/mL values of intracellular bacteria recovered from M1 and M2 macrophages at 4h (left) and 48h (right) p.i. with H37Rv, H37Ra, Beijing (L2), and Africanum (L6) strains.

Differences are observed according to macrophage polarization state, with increased bacterial entry in M2 cells across all MTB strains tested at 4h p.i. Higher CFU levels are detected in H37Ra-infected cells, whereas H37Rv and L6 display comparable intracellular burdens. In contrast, L2 uptake at 4h p.i. is markedly reduced compared to the other strains. Despite this lower initial intracellular CFU count, L2 shows substantial intracellular expansion, reaching CFU levels at 48h p.i. that are comparable to those observed for the other strains. Data are shown as mean ± SEM of replicate experiments.
