# Supplementary material for: Mycobacterium tuberculosis diversity and macrophage heterogeneity dictate phagosomal acidification
Source: Microbiol Spectr. 2026 Jul 20;14(9):e03762-25. doi: 10.1128/spectrum.03762-25 (PMC13532232; doi:10.1128/spectrum.03762-25)

Supplementary 7. Representative confocal images illustrating compartments acidification phenotypes.

For each strain, cell type, and time point, montage1 and merge1-3 correspond to independent representative confocal fields acquired from different wells and/or independent experimental replicates of the same condition. Channels are shown as nuclei (Hoechst, blue), acidified compartments (LysoSensor Green, green), and mycobacteria (mCherry, magenta). Images shown correspond to representative confocal fields of view; quantitative analyses presented in Figures 2-4 (main text) were performed at the single-cell level by segmentation and extraction of individual cell regions of interest (ROIs) from all acquired images. These images complement the quantitative single-cell analyses and illustrate the underlying cellular heterogeneity not captured by population-level measurements. Scale bar: 10 μm.

Representative confocal microscopy images of M1 macrophages (uninfected controls) at 6h post-infection

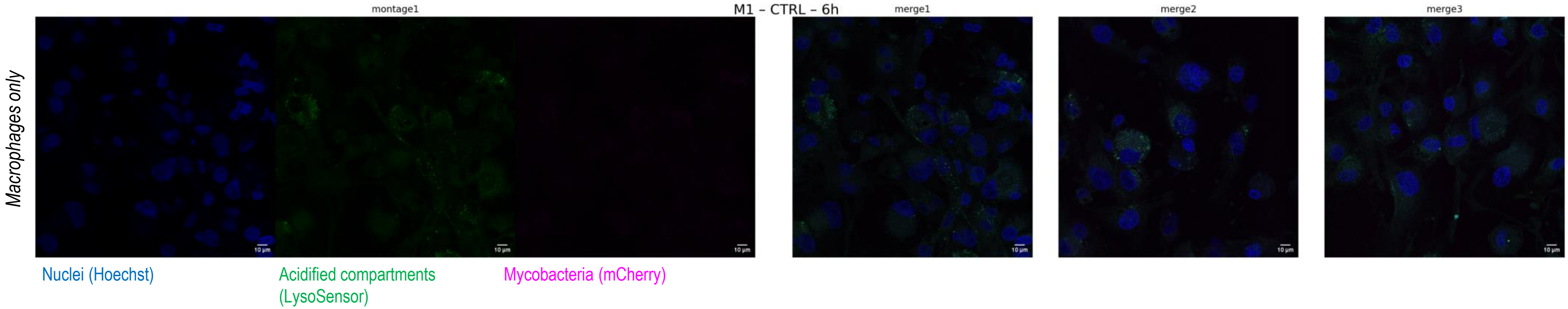

Representative confocal microscopy images of M1 macrophages infected with GEN MTBC strains at 6h post-infection

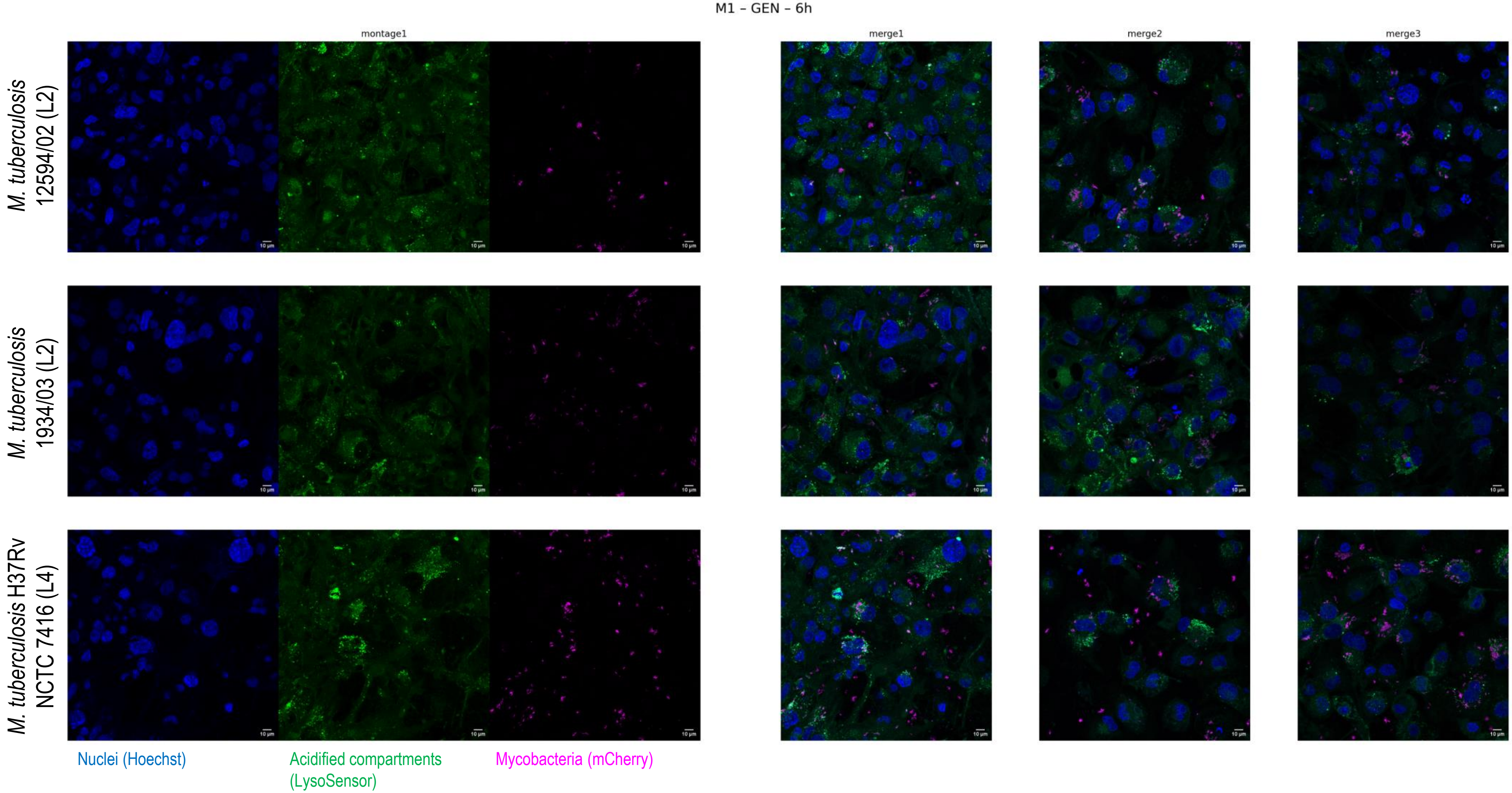

Representative confocal microscopy images of M1 macrophages infected with INT MTBC strains at 6h post-infection

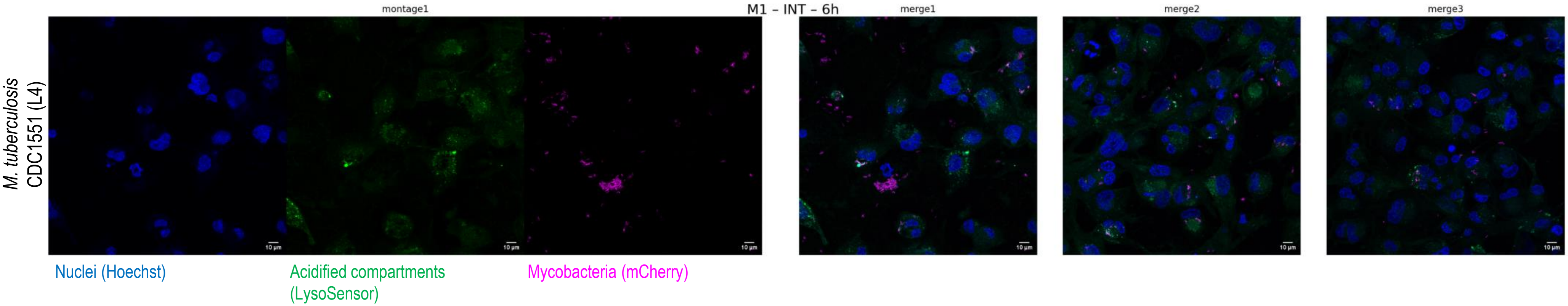

Representative confocal microscopy images of M1 macrophages infected with OTH MTBC strains at 6h post-infection

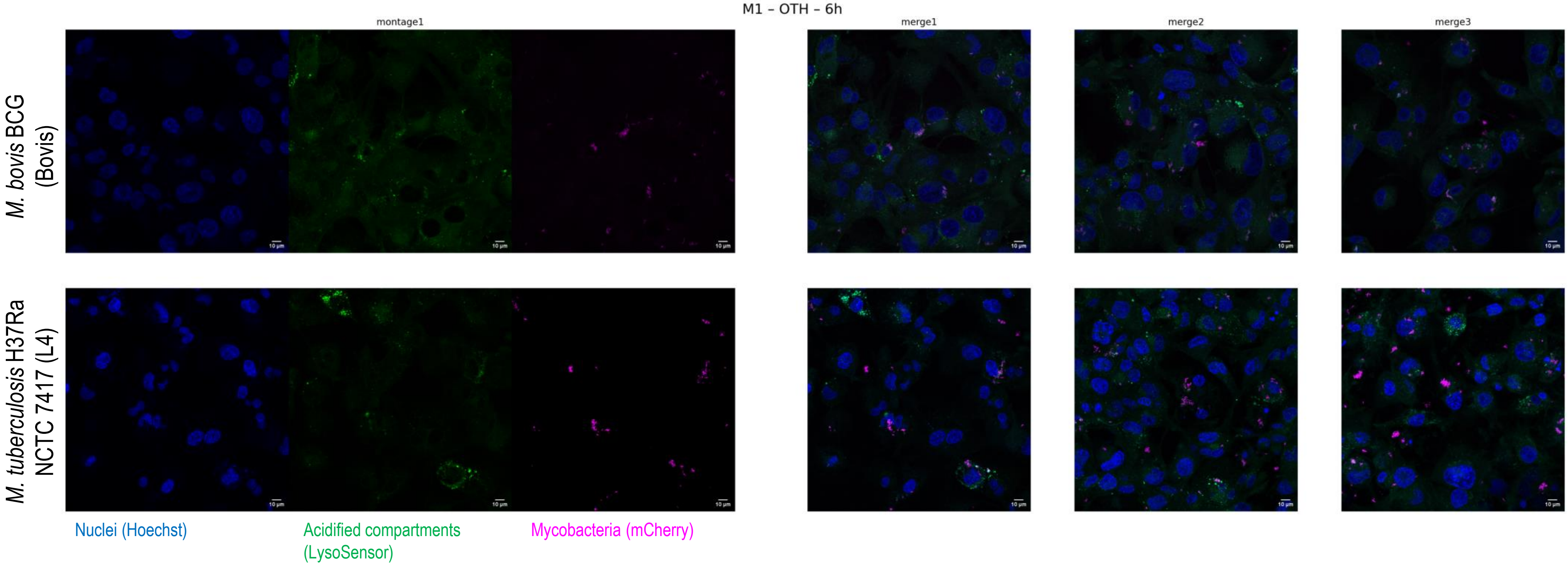

Representative confocal microscopy images of M1 macrophages infected with SPEC MTBC strains at 6h post-infection

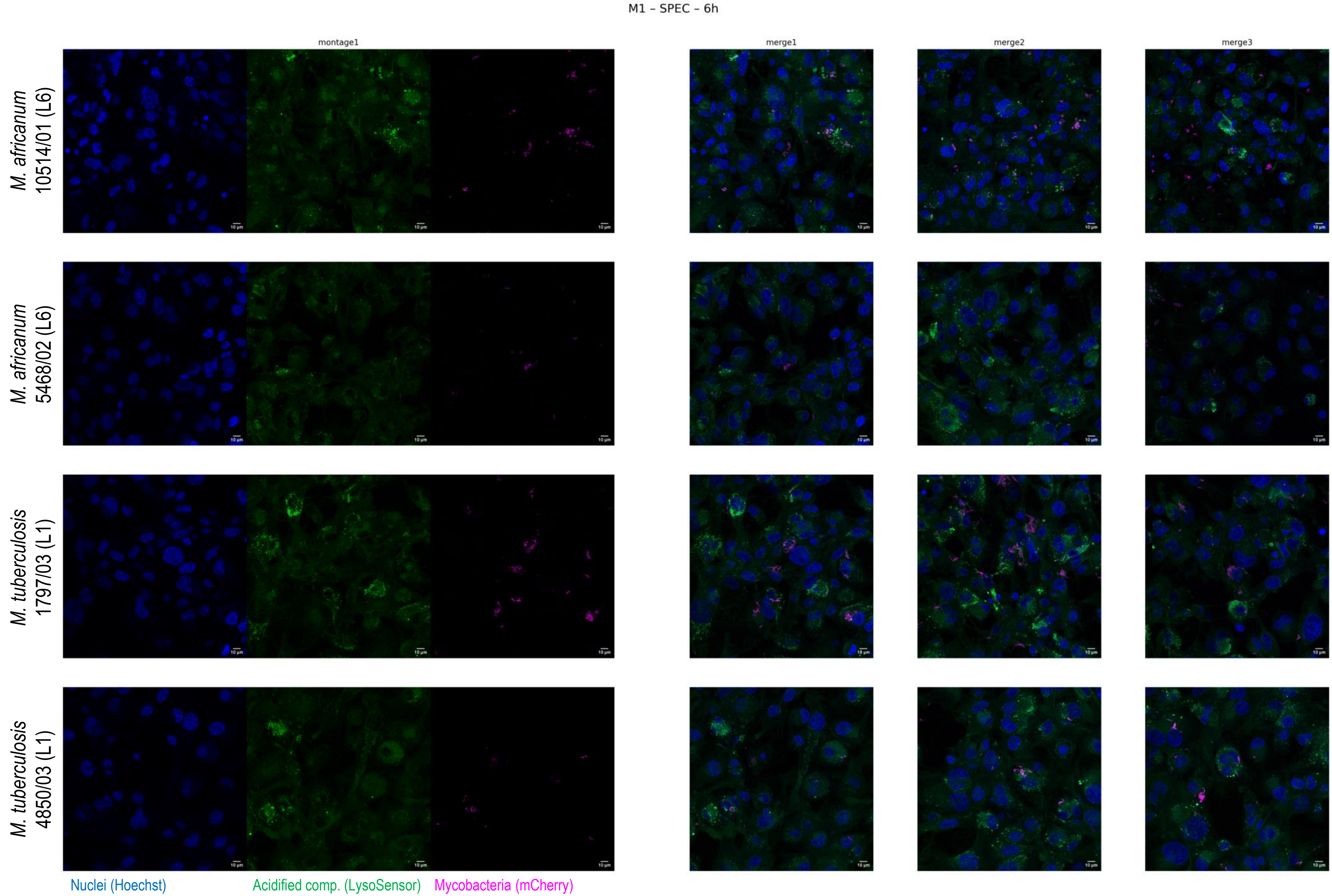

Representative confocal microscopy images of M2 macrophages (uninfected controls) at 6h post-infection

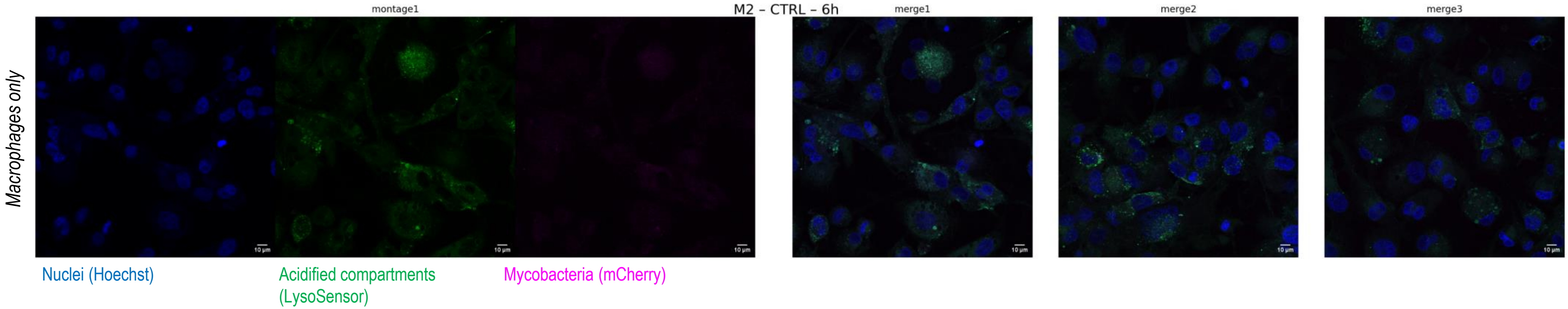

Representative confocal microscopy images of M2 macrophages infected with GEN MTBC strains at 6h post-infection

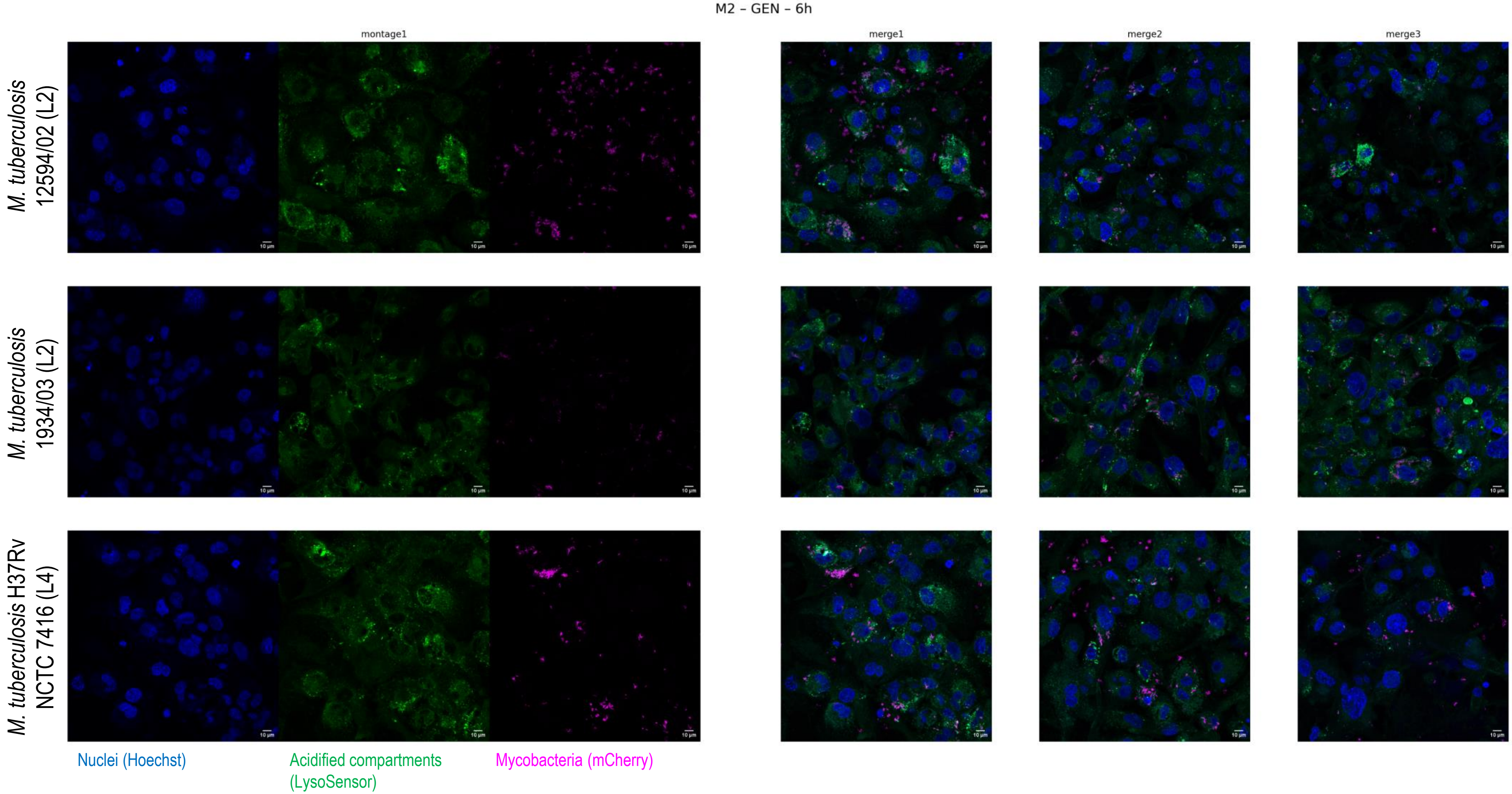

Representative confocal microscopy images of M2 macrophages infected with INT MTBC strains at 6h post-infection

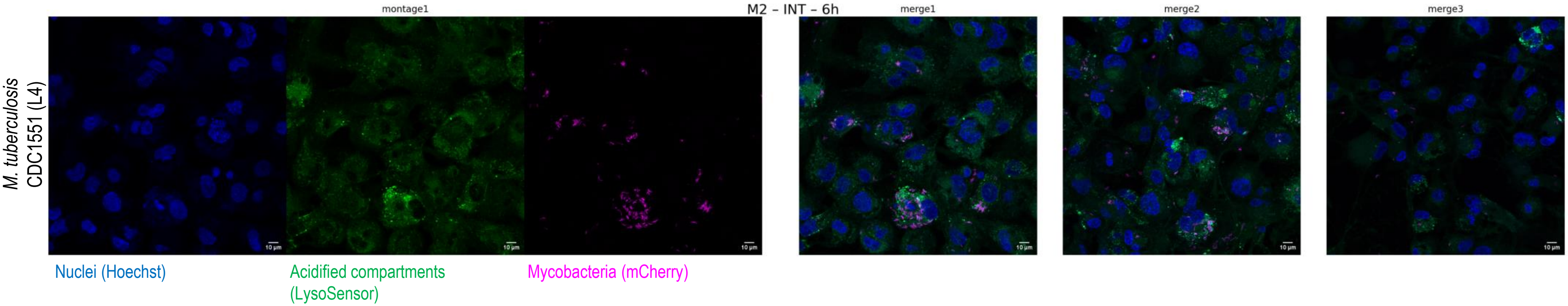

Representative confocal microscopy images of M2 macrophages infected with OTH MTBC strains at 6h post-infection

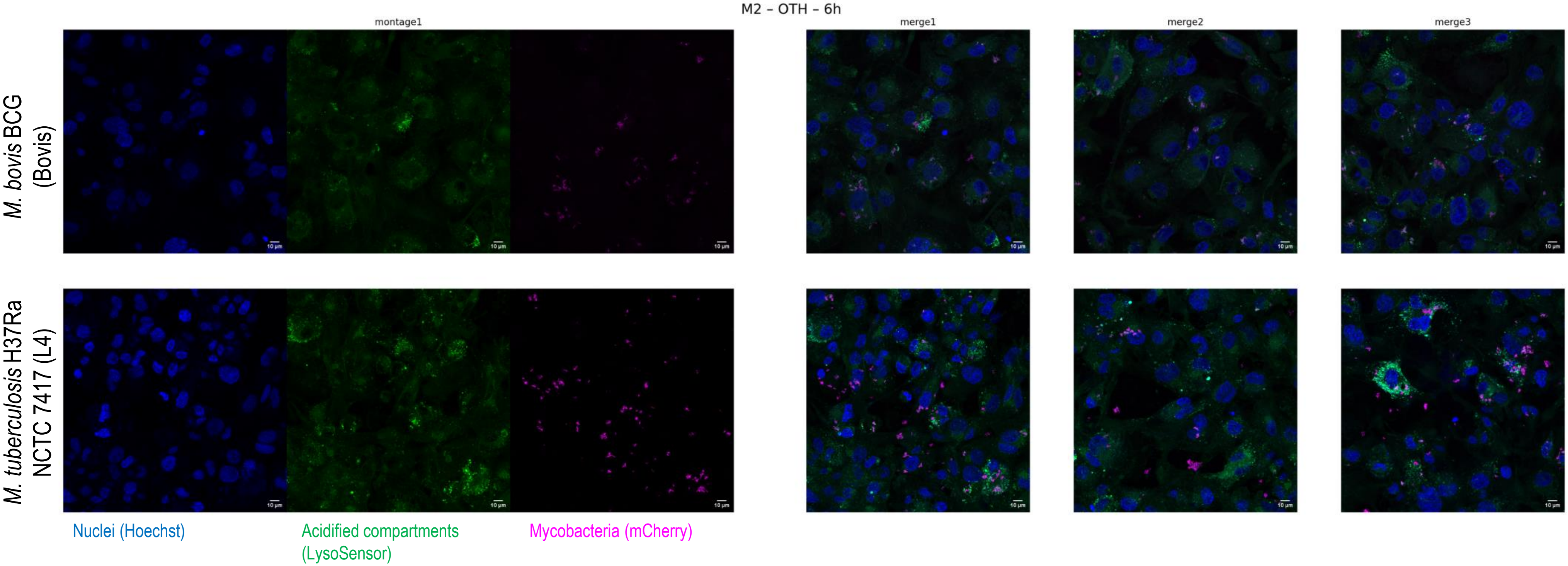

Representative confocal microscopy images of M2 macrophages infected with SPEC MTBC strains at 6h post-infection

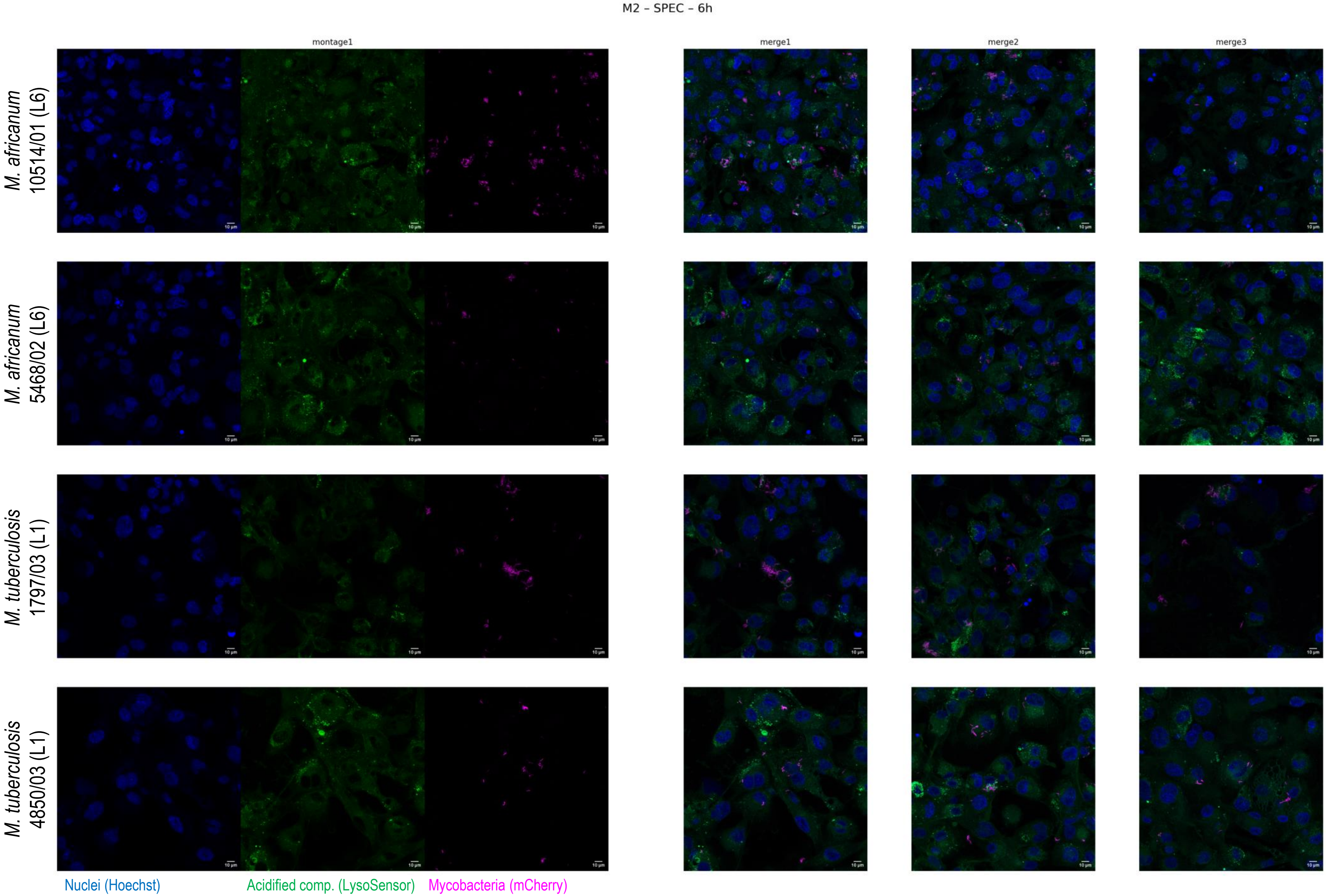

Representative confocal microscopy images of M1 macrophages (uninfected controls) at 24h post-infection

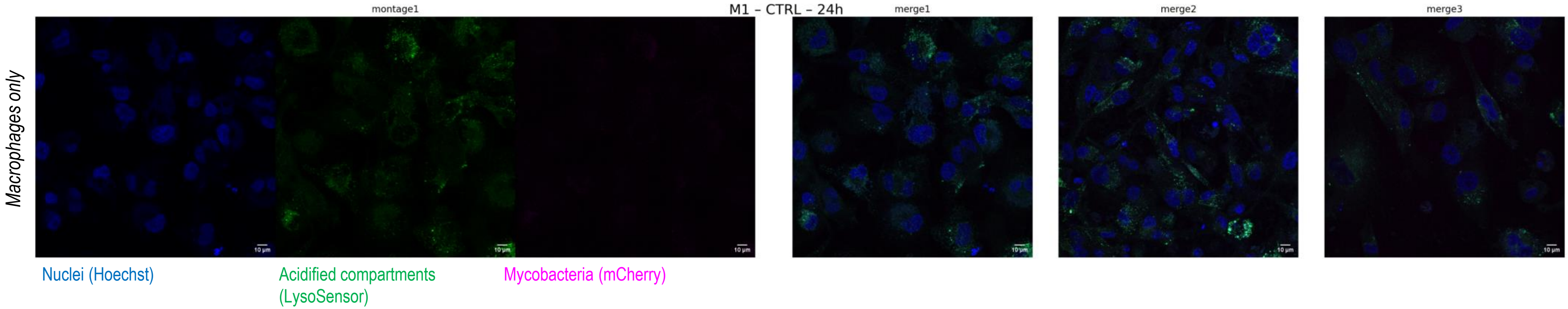

Representative confocal microscopy images of M1 macrophages infected with GEN MTBC strains at 24h post-infection

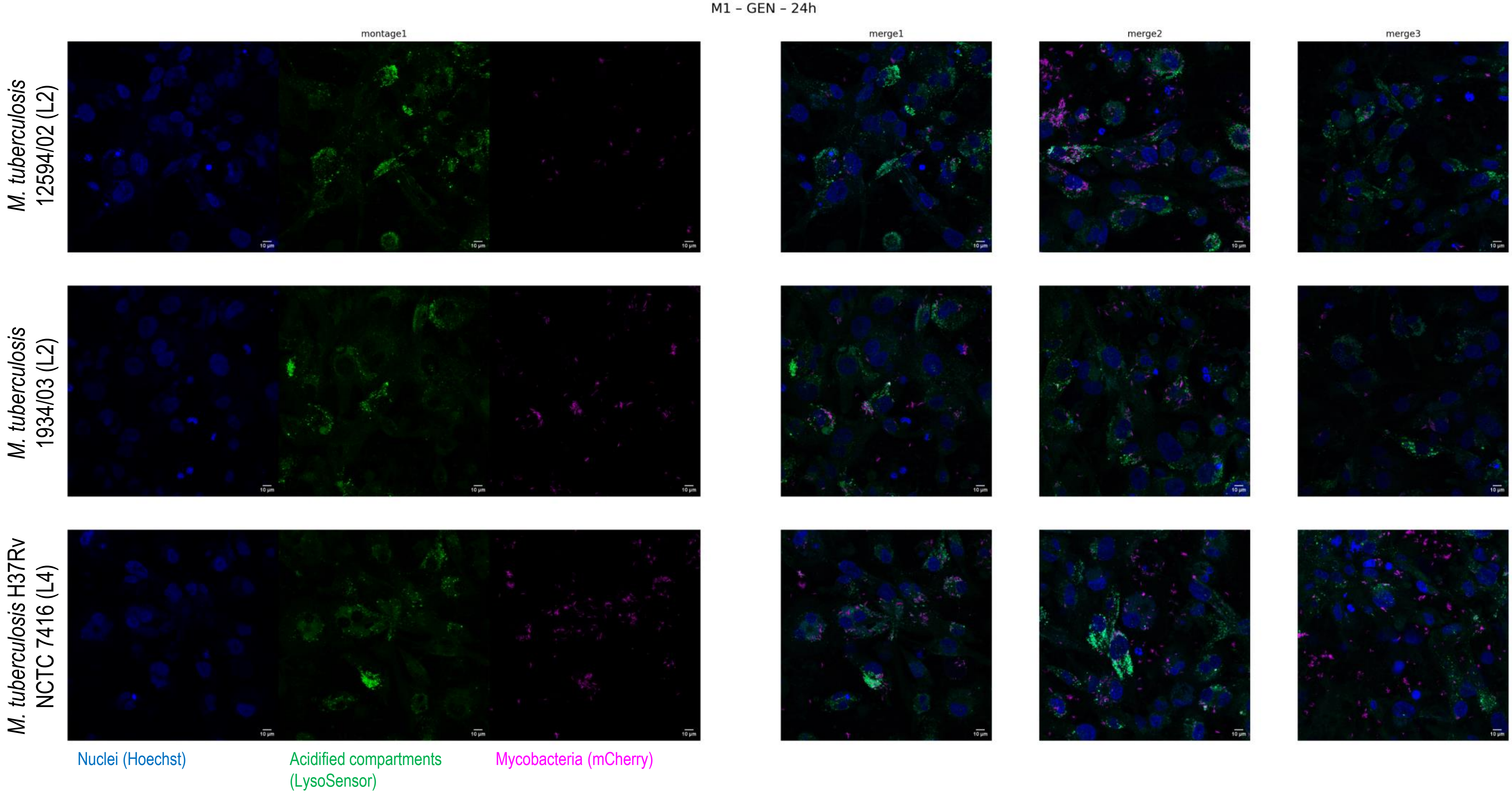

Representative confocal microscopy images of M1 macrophages infected with INT MTBC strains at 24h post-infection

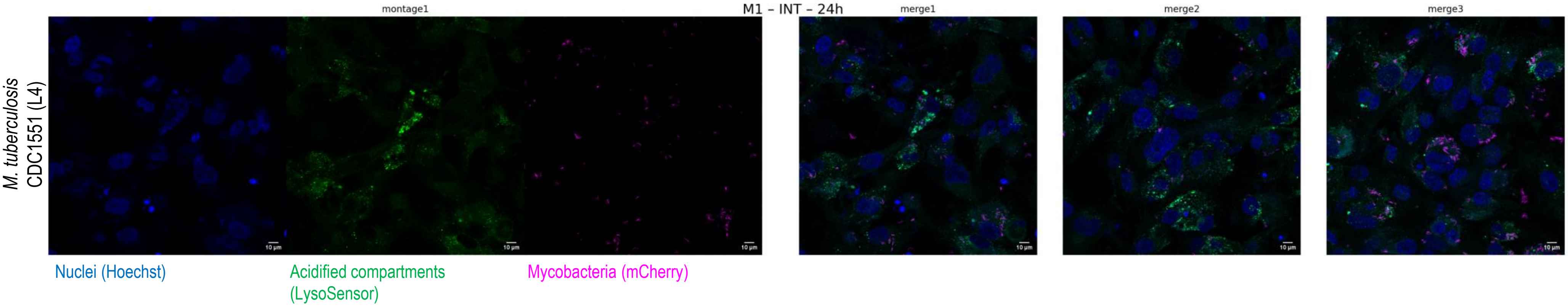

Representative confocal microscopy images of M1 macrophages infected with OTH MTBC strains at 24h post-infection

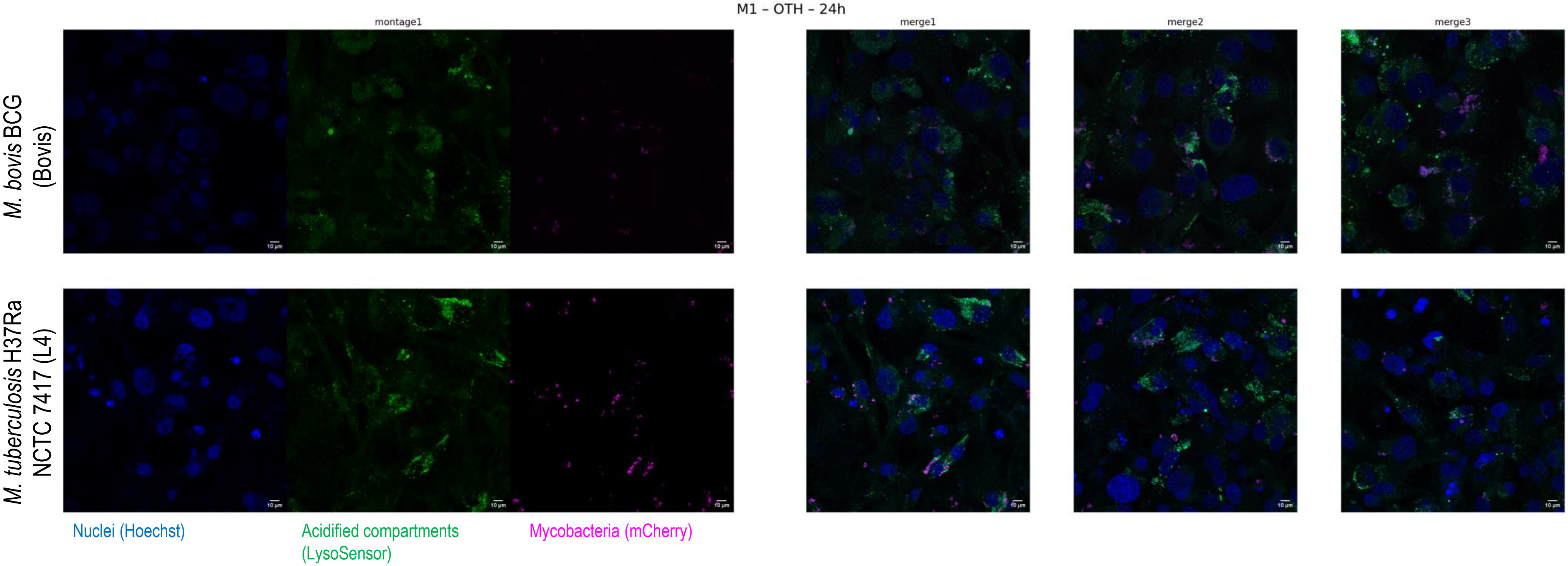

Representative confocal microscopy images of M1 macrophages infected with SPEC MTBC strains at 24h post-infection

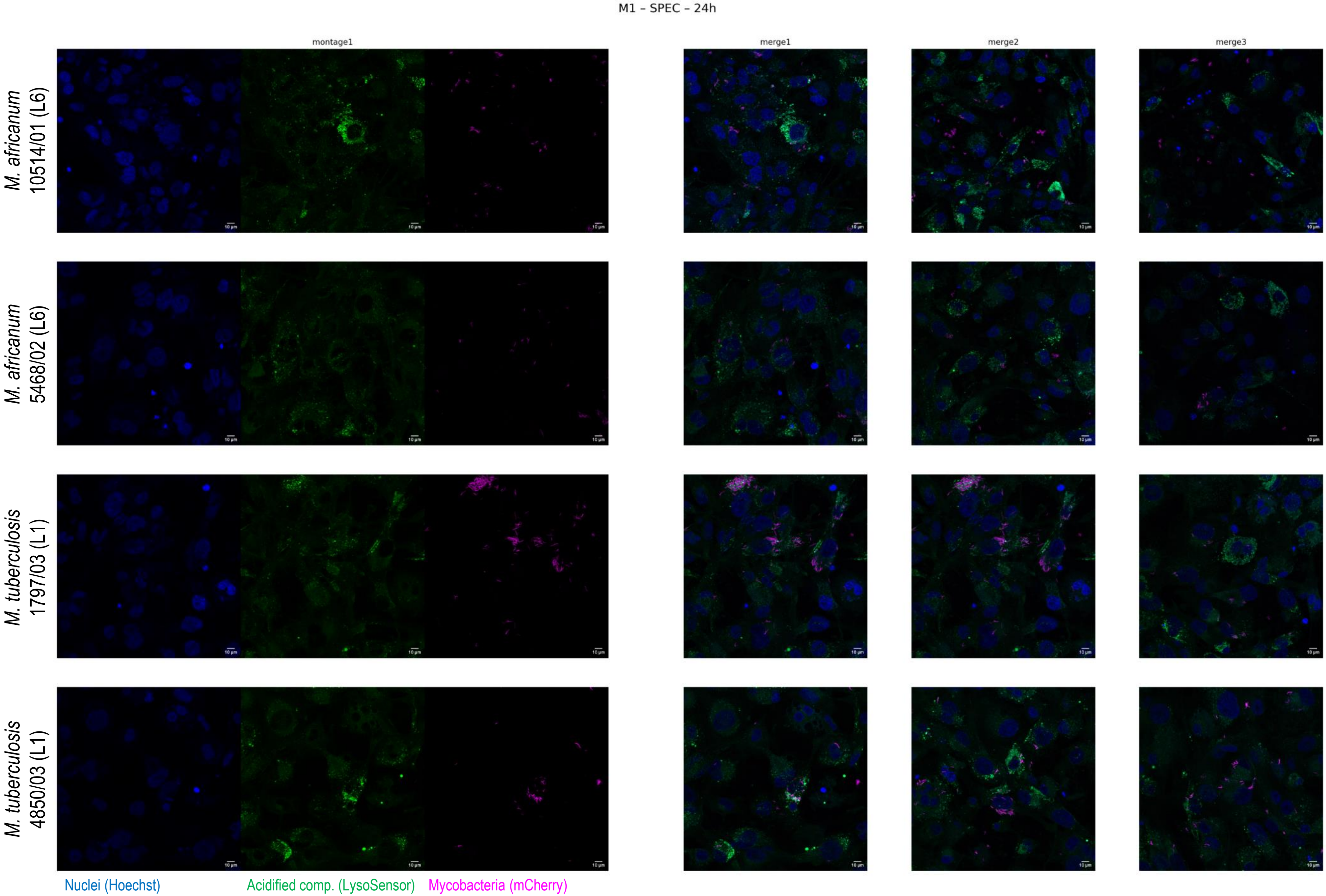

Representative confocal microscopy images of M2 macrophages (uninfected controls) at 24h post-infection

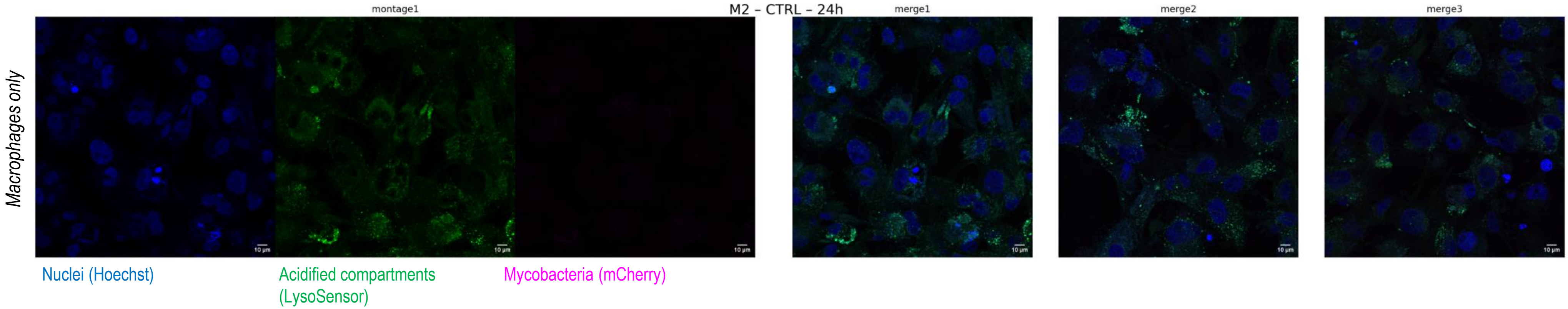

Representative confocal microscopy images of M2 macrophages infected with GEN MTBC strains at 24h post-infection

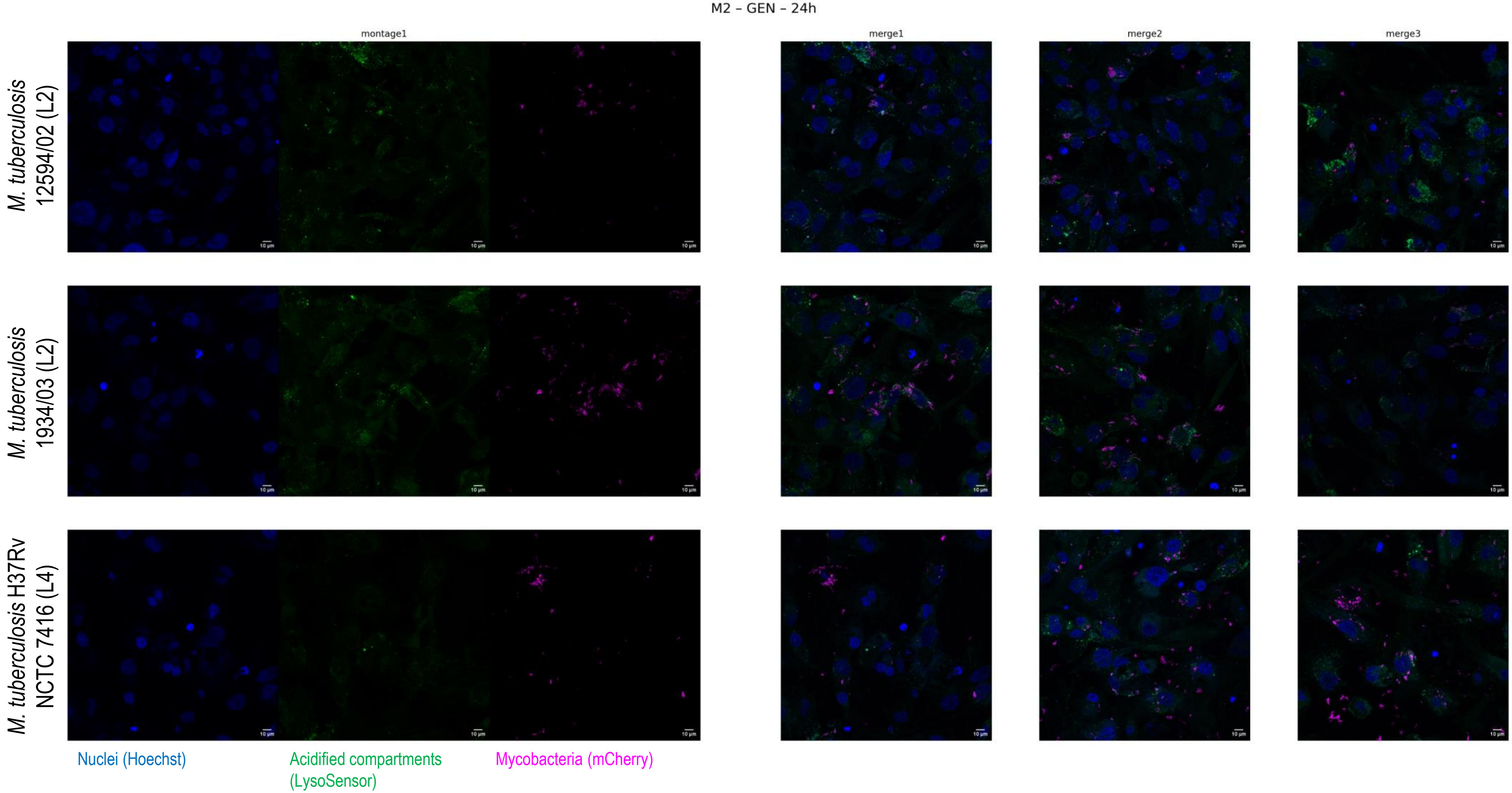

Representative confocal microscopy images of M2 macrophages infected with INT MTBC strains at 24h post-infection

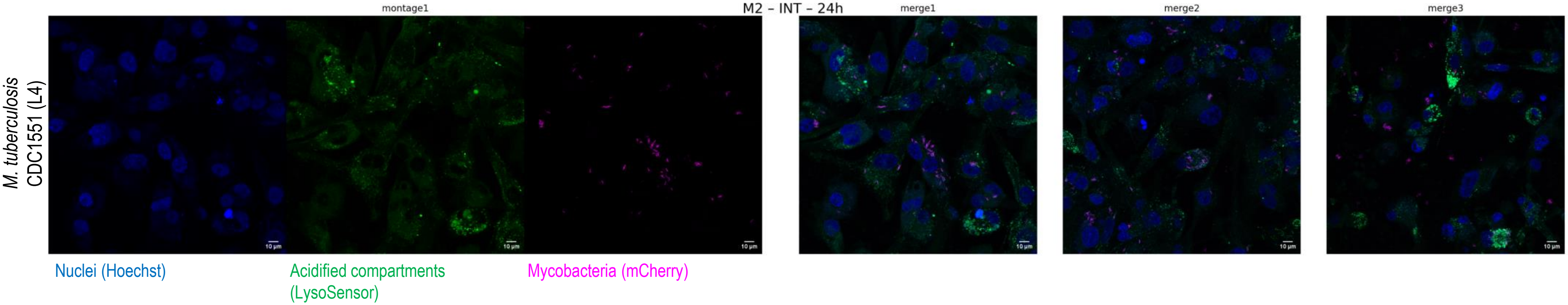

Representative confocal microscopy images of M2 macrophages infected with OTH MTBC strains at 24h post-infection

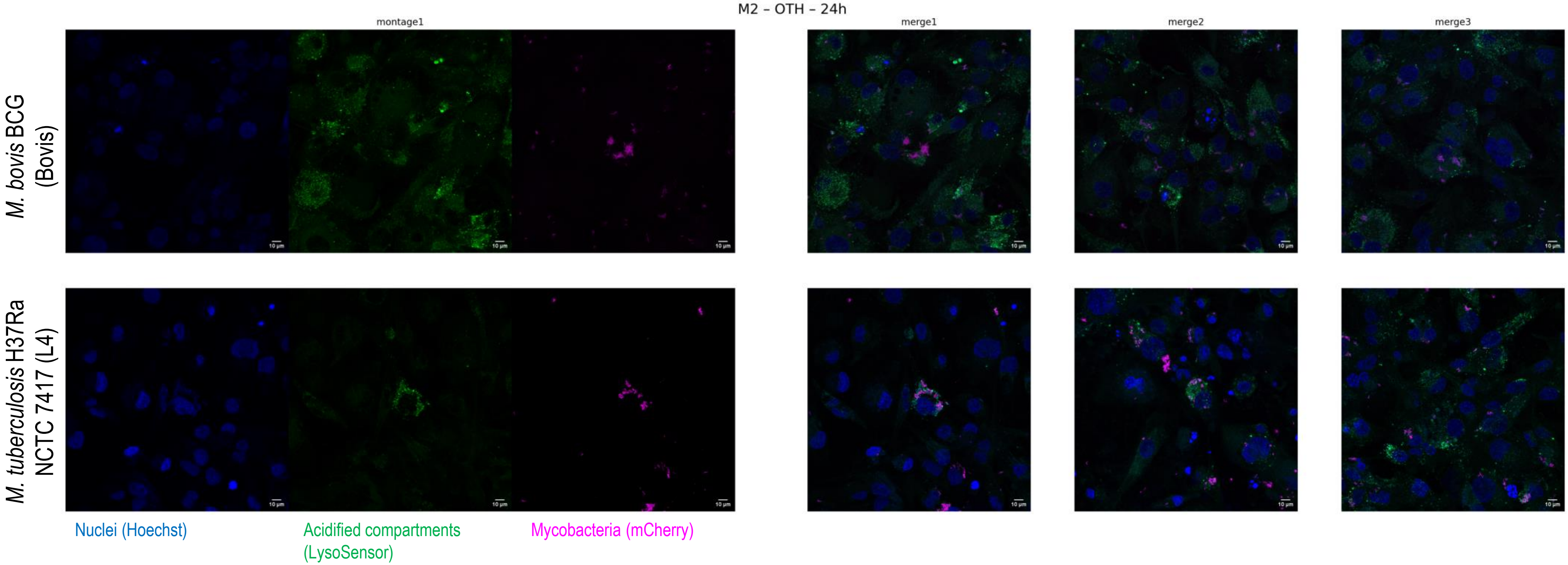

Representative confocal microscopy images of M2 macrophages infected with SPEC MTBC strains at 24h post-infection

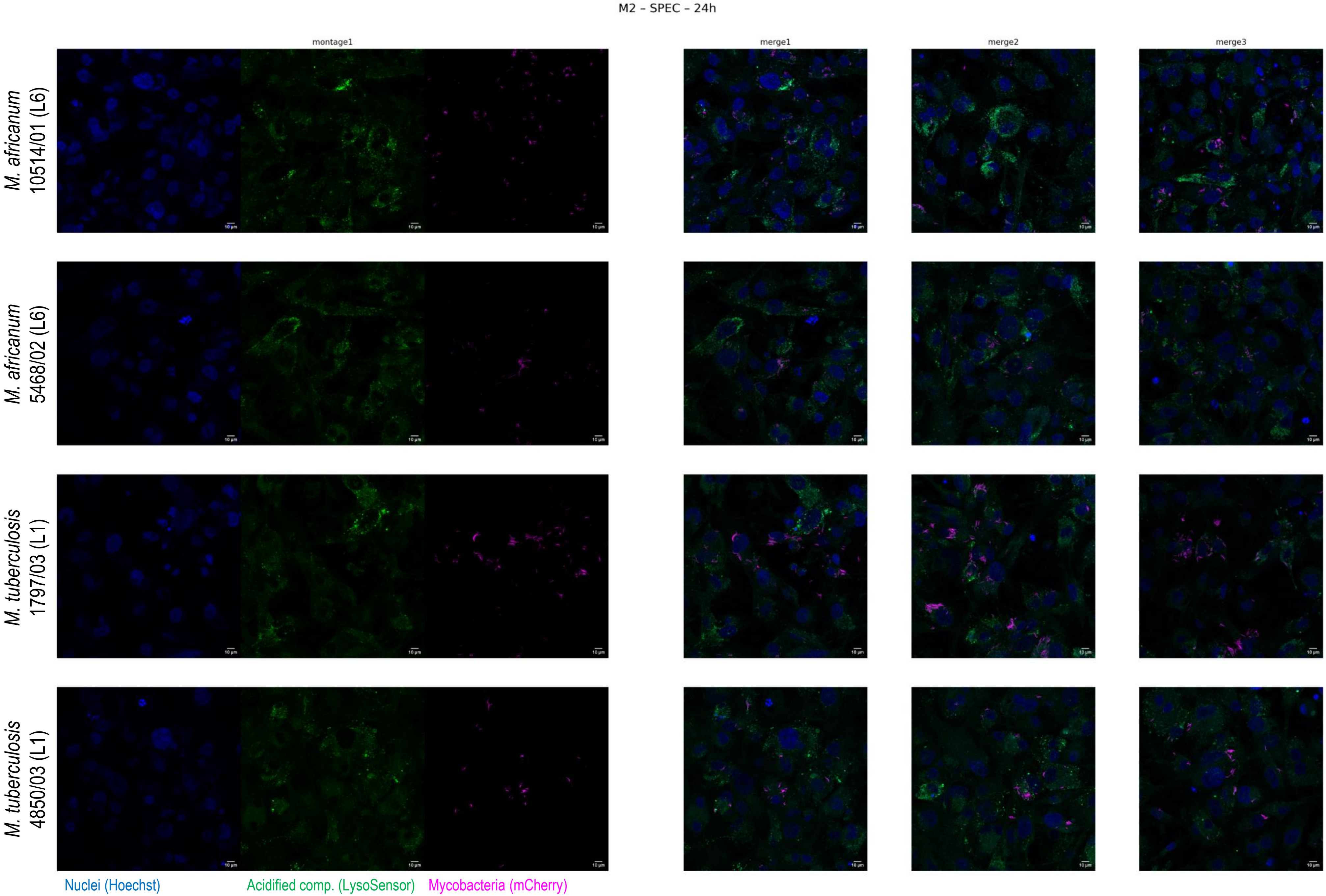

Supplement: Supplementary file 7 [file spectrum.03762-25-s0009.pdf]
