# Supplementary material for: Mycobacterium tuberculosis diversity and macrophage heterogeneity dictate phagosomal acidification
Source: Microbiol Spectr. 2026 Jul 20;14(9):e03762-25. doi: 10.1128/spectrum.03762-25 (PMC13532232; doi:10.1128/spectrum.03762-25)
Supplement: Supplementary file 8 [file spectrum.03762-25-s0001.docx]

Supplementary Figure S1: Expression profiling of specific markers for M1 (IL-1β, IL-6, CD80, TNF-α) and M2 (CD206) after polarization stimuli. Gene expression is provided as fold-change compared to M0 THP-1-derived macrophages (endogenous control: GAPDH).
